# Comparison of Phosphonium and Sulfoxonium Ylides in Ru(II)-Catalyzed Dehydrogenative Annulations: A Density Functional Theory Study

**DOI:** 10.3390/molecules30091883

**Published:** 2025-04-23

**Authors:** Wei Zhou, Lei Zhang, Dan-Yang Liu, Xiaosi Ma, Jie Zhang, Jiajia Kang

**Affiliations:** 1 School of Science, Tianjin Chengjian University, Tianjin 300384, China; veraz19@163.com (W.Z.); mxstcu@163.com (X.M.); zhangjie@tcu.edu.cn (J.Z.); wokangjia@126.com (J.K.); 2 College of Chemistry, Beijing Normal University, Beijing 100875, China; 202331150041@mail.bnu.edu.cn

**Keywords:** ruthenium-catalyzed, C–H activation, annulation, ylide, reaction mechanism, DFT calculation

## Abstract

Density functional theory calculations have been performed to explore the detailed mechanism of a ruthenium-catalyzed dehydrogenative annulation between α-carbonyl phosphonium ylide (**A**) and sulfoxonium ylide (**B**). The proposed catalytic cycles consist of several elementary steps in succession, namely the C–H activation of ylide **A**, the insertion of ylide **B**, reductive elimination, protodemetallation, and an intramolecular Wittig reaction, in which C–H activation is rate-limiting, with a free energy barrier of 31.7 kcal/mol. As **A** and **B** are both capable of being a C–H activation substrate and a carbene precursor, there are potentially four competing pathways including homo-coupling reactions. Further calculations demonstrate that **A** is more reactive in the C–H activation step than **B**, while the opposite conclusion is true for the ylide insertion step, which can successfully explain the fact that the solely observed product originated from the use of **A** as the C–H activation substrate and **B** as the carbene precursor. Molecular electrostatic potential, charge decomposition, and electron density difference analyses were performed to understand the distinct behaviors of the two ylides and the nature of the key ruthenium–carbene intermediate.

## 1. Introduction

Transition-metal-catalyzed C–H activation has emerged as a powerful and straightforward method for the construction of various carbon–carbon or carbon–heteroatom bonds [1,2,3,4,5]. It allows the direct coupling of two separate reactants involving the cleavage of one or two C–H bonds [6,7,8,9]. More excitingly, dehydrogenative annulation reactions via C–H activation can offer a convenient tool for the assembly of complex cyclic skeletons from simple starting materials [10,11,12].

In recent years, α-carbonyl sulfoxonium ylides have received substantial attention and have been widely employed in C–H functionalizations due to their special reactivity modes [13,14]. On the one hand, owing to the presence of a carbonyl directing group, α-carbonyl sulfoxonium ylides are competent C–H activation substrates for cyclometallation. On the other hand, α-carbonyl sulfoxonium ylides are capable of being carbene precursors for the generation of metal–carbene species. A considerable amount of attention has been paid to the transition-metal-catalyzed C–H functionalization reactions with an α-carbonyl sulfoxonium ylide serving as either a C–H activation substrate [15,16,17] or a carbene precursor [18,19,20].

**Scheme 1:**
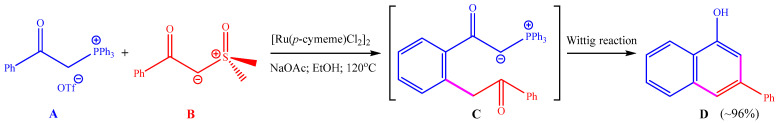
Ruthenium-catalyzed dehydrogenative annulation of α-carbonyl phosphoniums and sulfoxonium ylides that were studied in this work using computations.

Chen and co-workers reported a ruthenium-catalyzed cross-coupling annulation between two different ylide reagents (see Scheme 1) [21]. The use of α-carbonyl phosphoniums **A** and sulfoxonium ylides **B** as the reactants, [Ru(*p*-cymene)Cl_2_]_2_ (*p*-cymene = 4-isopropyltoluene) as the catalyst, and NaOAc as the base in ethanol solvent at 120 °C furnished 1-naphthols **D** in high yields via C–H activation, C–C coupling, and Wittig reaction sequences. The final product **D** was believed to be generated from the precursor **C** through an intramolecular Wittig reaction.

**Scheme 2:**
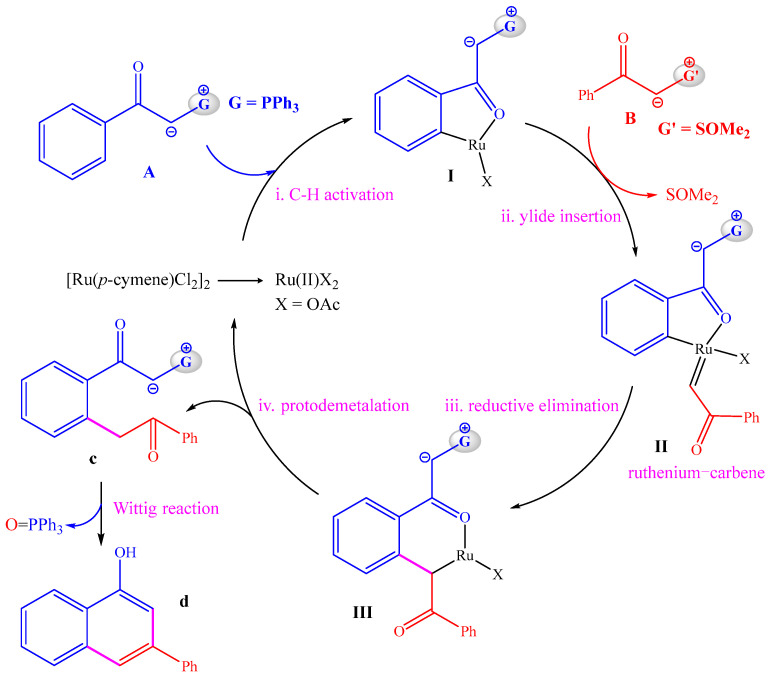
Proposed catalytic cycles.

The proposed reaction pathway on the basis of experimental observations is shown in Scheme 2. Before the reaction, anion exchange between [Ru(*p*-cymene)Cl_2_]_2_ and NaOAc can generate a mononuclear Ru(II) compound as the active catalyst. First, C–H activation of phosphonium ylide **A** with the Ru(II) catalyst delivers the five-member ruthenacycle **I**, which is followed by the insertion of sulfoxonium ylide **B** to afford the ruthenium–carbene species **II** containing a Ru=C double bond. Then, a reductive elimination reaction is required for the construction of a C–C bond between the carbene group and the aryl group, leading to the six-member ruthenacycle **III**. Afterwards, protodemetallation of the Ru–C bond in **III** can release the precursor of the product **C** and regenerate the Ru(II) catalyst. Finally, an intramolecular Wittig reaction can transform **C** into the final product **D** with the release of O=PPh_3_. Kinetic isotope experiments with a *k*_H_/*k*_D_ value of 2.1 showed the C–H activation step to be rate-limiting [21].

Despite the synthetic advances achieved before, some key mechanistic issues, especially the comparison of the reactivities of α-carbonyl phosphonium and sulfoxonium ylides in different steps, are still unexplored. As density functional theory (DFT) calculations have been widely used in the study of transition-metal-catalyzed organic reaction mechanisms [22,23,24,25], the present work presents a series of DFT characterizations on the title reaction in Scheme 1 to address the key mechanistic issues in detail.

## 2. Results and Discussion

**Scheme 3:**
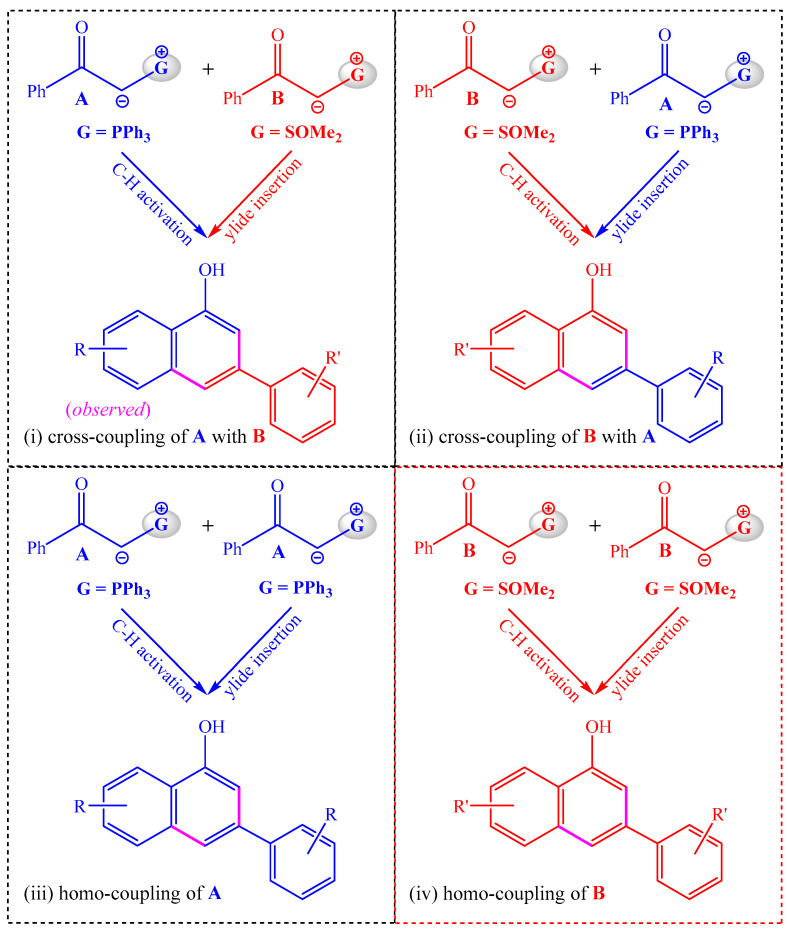
Possible modes of the annulation reaction.

As phosphonium ylide **A** and sulfoxonium ylide **B** are structurally similar, they can serve as both the substrate of the C–H activation step and the reagent of the ylide insertion step. Hence, there are four possible annulation modes in the reaction system of **A** and **B**, as shown in Scheme 3. In mode i, **A** serves as the C–H activation substrate and **B** serves as the reagent of the ylide insertion, and this annulation model is the same as that shown in Scheme 2, leading to the cross-coupling of **A** with **B**. In mode ii, the C–H activation substrate is **B** and the carbene precursor is **A**, which would lead to the cross-coupling of **B** with **A**. In modes iii and iv, two molecules of the same ylide are used in both the C–H activation step and the insertion step, which would generate homo-coupling products.

If there are no substituents on the aryl rings of **A** and **B**, the four annulation modes will give the identical 1-naphthol product. By employing the diversely substituted α-carbonyl phosphonium and sulfoxonium ylides, the solely observed product originated from annulation model i. In order to probe the competition of different annulation modes, the following two questions need to be answered: (1) Which is the better C–H activation substrate, phosphonium ylide **A** or sulfoxonium ylide **B**, and (2) Which is the better precursor for the formation of ruthenium–carbene species in the insertion step, phosphonium ylide **A** or sulfoxonium ylide **B**? The favorable reaction pathways and the distinct reactivities of phosphonium and sulfoxonium ylides in the C–H activation and insertion steps will be disclosed in the following subsections.

### 2.1. Reaction Mechanism and Free Energy Profiles

#### 2.1.1. C–H Activation Process

According to previous reports in the literature [26,27,28], the binuclear ruthenium compound [Ru(*p*-cymene)Cl_2_]_2_ could be transformed to the mononuclear ruthenium catalyst Ru(OAc)_2_(*p*-cymene) in the presence of a suitable acetate additive, and the latter species serves as the active catalyst for many ruthenium-catalyzed systems. In this regard, we have evaluated the transformation of the catalyst from [Ru(*p*-cymene)Cl_2_]_2_ to Ru(OAc)_2_(*p*-cymene) with NaOAc as the acetate source, and the calculated Gibbs free energy change is −10.3 kcal/mol, confirming the thermodynamic spontaneity for this transformation (see Supplementary Materials for details). Therefore, the use of Ru(OAc)_2_(*p*-cymene) as the active catalyst (**CAT**) is reasonable. At the same time, reactant **A** in the original form of phosphonium salt can be converted to the ylide form through deprotonation by NaOAc, with a free energy change of −10.8 kcal/mol.

**Figure 1:**
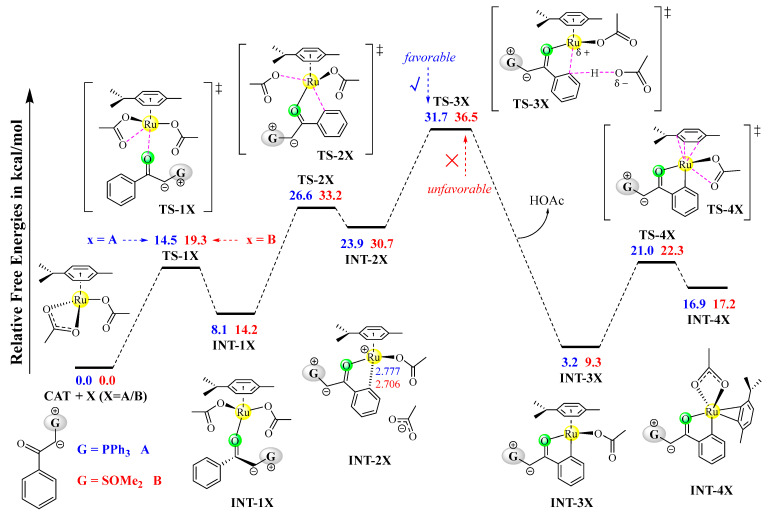
Mechanism and free energy profiles of the C–H activation processes of **CAT**+**A** and **CAT**+**B**, determined at the B3LYP+D3BJ-PCM/def2TZVPP//B3LYP+D3BJ/def2SVP level. Each transition state is labeled with a “‡” superscript in its molecular structure.

The detailed mechanism and free energy profiles of the C–H activation processes catalyzed by **CAT** are depicted in Figure 1. In order to compare the reactivity of phosphonium ylide **A** and sulfoxonium ylide **B** as the C–H activation substrates, the C–H activation pathways of **CAT**+**A** and **CAT**+**B** are both shown in Figure 1. The carbonyl oxygen of **X** (**X**=**A**/**B**) serves as a coordination center rendering the substrate–catalyst combination, which is realized by a ligand-exchange process via transition state **TS-1X**, leading to intermediate **INT-1X**. Then, one of the κ^1^-OAc ligands is removed by the formation of a weak Ru–C interaction via transition state **TS-2X**, which affords the ion pair intermediate **INT-2X** as the precursor of the C–H activation step. The OAc anion in the outer sphere is used as a base to abstract the hydrogen atom from the central carbon, passing through the proton-transfer transition state **TS-3X** to yield the ruthenacycle intermediate **INT-3X** with the release of acetic acid. In order for the entry of the second molecule of ylide (**A** or **B**) into the ligand field of **INT-3X**, the sterically demanding *p*-cymene ligand needs to be removed from the ruthenium center. The change in the coordination mode of the *p*-cymene ligand from η^6^ to η^2^ via the transition state **TS-4X** should be necessary for forming the final structure **INT-4X** in the C–H activation pathway.

It can be observed from the free energy profiles in Figure 1 that all the stationary points in the **A**+**CAT** pathway are lower than their counterparts in the **B+CAT** pathway, especially for the stationary points before **TS-4X**, in which the former stationary points are 4.8~6.8 kcal/mol lower in free energy than the latter ones. These results indicate that C–H activation of **A**+**CAT** is kinetically more favorable than that of **B**+**CAT**, and hence, phosphonium ylide **A** should be a better C–H activation substrate than sulfoxonium ylide **B**. The proton-transfer transition state **TS-3X** is the highest stationary point and is thus rate-determining, which is consistent with the primary kinetic isotope effect (KIE = 2.1) observed experimentally. According to the energetic span model [29,30], the rate-determining free energy barriers of the C–H activation reactions of **A** and **B** are 31.7 and 36.5 kcal/mol, respectively, and the 4.8 kcal/mol difference in the activation barrier indicates that sulfoxonium ylide **B** is unable to compete with phosphonium ylide **A** towards C–H activation with **CAT**.

#### 2.1.2. Coupling and Annulation Processes

**Figure 2:**
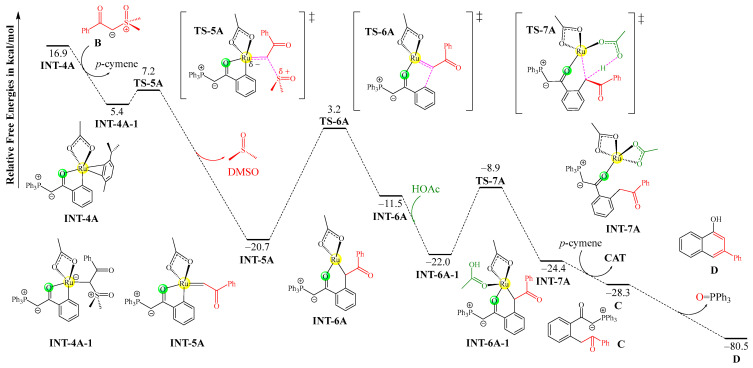
Mechanism and free energy profiles of the remaining steps after C–H activation, determined at the B3LYP+D3BJ-PCM/def2TZVPP//B3LYP+D3BJ/def2SVP level. Each transition state is labeled with a “‡” superscript in its molecular structure.

As the C–H activation process of **A**+**CAT** is kinetically more favorable than that of **B**+**CAT**, the ruthenacycle species **INT-4A** should be preferentially formed as an intermediate product for the following insertion reaction with sulfoxonium ylide **B**. The corresponding mechanism and free energy profiles for the reaction of **INT-4A** with **B** forming product **D** are depicted in Figure 2. First, the η^2^-ligated *p*-cymene is removed by the addition of the carbanion center of **B** onto the ruthenium center, generating the precursor complex **INT-4A-1**. Then, the departure of the sulfoxide (-SOMe_2_) group via transition state **TS-5A** yields the ruthenium–carbene species **INT-5A**, featuring a Ru=C double bond, and releases a molecule of DMSO as a co-product. The following reductive elimination step via **TS-6A** is responsible for the construction of a C–C bond between the carbene moiety and the adjacent aryl group, resulting in the ring expansion of the ruthenacycle. The resulting intermediate **INT-6A** is coordinated by the acetic acid molecule that was previously released to form intermediate **INT-6A-1**. Afterwards, **INT-6A-1** undergoes a protodemetallation reaction to break the Ru–C bond via the proton-transfer transition state **TS-7A**, in which the coordinated acetic acid delivers its hydrogen atom to the carbon center, leading to intermediate **INT-7A**. The precursor of the product (**C**) can be separated from the ruthenium center by the re-coordination of the *p*-cymene ligand, with the regeneration of the catalyst **CAT**. Finally, **C** can undergo a typical Wittig reaction in an intramolecular fashion to furnish 1-naphthol **D** from two different ylide reagents.

The ylide insertion process is favorable both kinetically and thermodynamically because the ligand exchange transforming **INT-4A** to **INT-4A-1** lowers the free energy by 11.5 kcal/mol, and the subsequent departure of the -SOMe_2_ group involves a relatively low free energy barrier of 1.8 kcal/mol, with a free energy release of 26.1 kcal/mol. The reductive elimination and protodemetallation steps involve moderate barriers of 23.9 and 13.1 kcal/mol, respectively. The final product transformation through an intramolecular Wittig reaction is strongly spontaneous.

**Figure 3:**
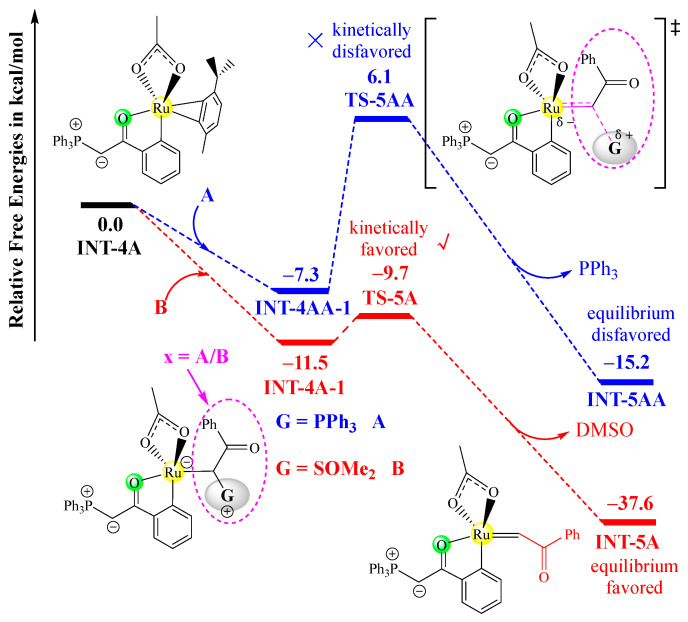
Free energy profiles of the insertion step of **INT-4** with **A** (in blue) or **B** (in red), determined at the B3LYP+D3BJ-PCM/def2TZVPP//B3LYP+D3BJ/def2SVP level. The transition state is labeled with a “‡” superscript in its molecular structure.

In fact, phosphonium ylide **A** can also undergo a similar insertion process with **INT-4A** to yield the corresponding ruthenium–carbene intermediate, which would result in the homo-coupling of two molecules of **A**. In Figure 3, the ylide insertion processes of both **A** and **B** with **INT-4A** and the corresponding free energy profiles are comparatively shown. Obviously, the insertion process of a sulfoxonium ylide proceeds more favorably than that of a phosphonium ylide. Firstly, the ylide-binding complex **INT-4A-1** is 4.2 kcal/mol more stable than **INT-4AA-1**. Secondly, the free energy barrier for the removal of DMSO is much lower than that for the removal of PPh_3_ (1.8 kcal/mol vs. 13.4 kcal/mol), and the large difference in the activation barrier indicates that the phosphonium ylide insertion is not kinetically competitive at all. Lastly, the release of DMSO would release more free energy that of PPh_3_. These results demonstrate that sulfoxonium ylide **B** is more active in the insertion process than phosphonium ylide **A**, and hence, the homo-coupling of two molecules of **A** can be ruled out.

#### 2.1.3. Overview of the Free Energy Variations

**Figure 4:**
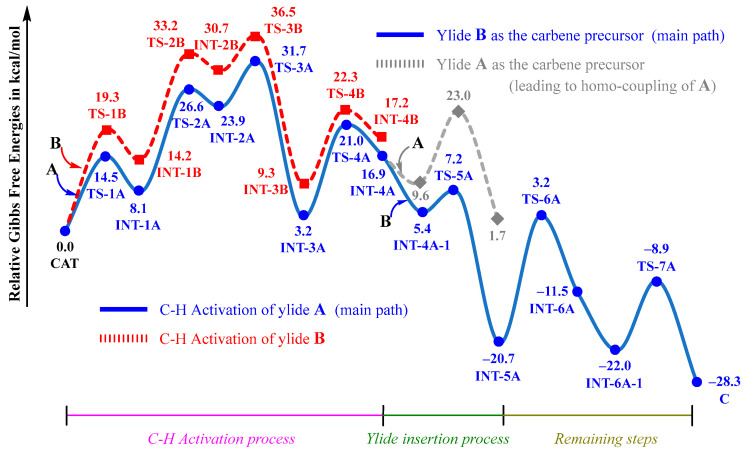
Entire free energy profiles, determined at the B3LYP+D3BJ-PCM/def2TZVPP//B3LYP+D3BJ/def2SVP level.

The entire free energy profiles for the observed cross-coupling of **A** with **B** are given in Figure 4. For comparison, the potentially competing pathways are also given in Figure 4. Obviously, the C–H activation process with a free energy barrier of 31.7 kcal/mol is the rate-limiting process, which is consistent with the kinetic isotope effect (KIE = 2.1) and the experimental temperature of 120 °C. Although sulfoxonium ylide **B** is also able to undergo a C–H activation reaction with **CAT**, the proton-transfer transition state **TS-3B** is 4.8 kcal/mol higher than **TS-3A**, which could negate its competition and further rule out the possibilities of both the homo-coupling of **B** and the cross-coupling of **B** with **A** (see modes ii and iv in Scheme 3).

The ruthenacycle intermediate **INT-4A** could be inserted by either sulfoxonium ylide **B** or phosphonium ylide **A**. The insertion of **B**, which forms the ruthenium–carbene species **INT-4A**, involves a relatively low barrier of 1.8 kcal/mol, which is much smaller than that of the insertion of **A**. It is understandable that the homo-coupling of **A** (see model iii in Scheme 3) can be effectively suppressed.

As the rate-determining activation barrier is approximate to the free energy difference between **TS-3A** and **CAT**+**A**, the overall reaction should follow a second-order kinetic law and is first order with respect to both **CAT** and **A**. Therefore, **A** is the reactivity-determining reagent, which is consistent with the primary kinetic isotope effect on **A** observed experimentally [21]. Some previous mechanistic studies on ruthenium-catalyzed C–H functionalizations also revealed that C–H activation of the ruthenium catalyst with a substrate is the rate-determining process [26,28].

#### 2.1.4. Explicit Solvation Effect

**Figure 5:**
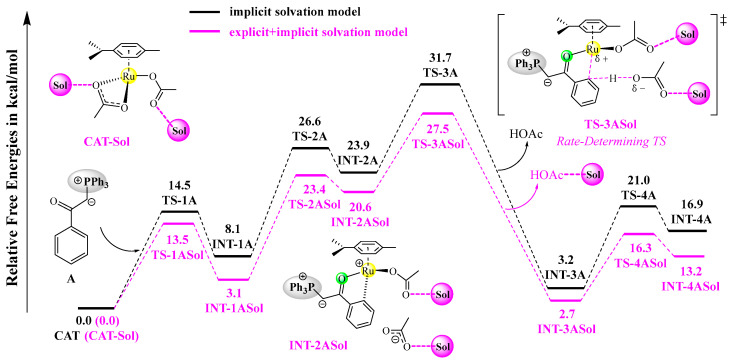
Free energy profiles of the C-H activation reaction of **A** with **CAT** based on the explicit solvation model at the B3LYP+D3BJ-PCM/def2TZVPP//B3LYP+D3BJ/def2SVP level. Each transition state is labeled with a “‡” superscript in its molecular structure.

According to experimental observations, ethanol was the best solvent, generating the desired products in high yields (~96%), while some common aprotic solvents (e.g., 1,2-dichloroethane) delivered moderate yields (~35%) [21]. The observed solvent effects indicate that the explicit participation of ethanol may lower the activation barriers, possibly through hydrogen-bonding interactions. As the C–H activation process is rate-determining, we have tested the explicit solvation effect on the C–H activation reaction of **A** with **CAT** by adding two molecules of ethanol around the two acetate groups of the catalyst, and the resulting free energy profiles are shown in Figure 5. All of the stationary points become lower in free energy by incorporating two solvent molecules, as compared to those calculated by an implicit solvation method. Importantly, the rate-determining free energy barrier is lowered from 31.7 to 27.5 kcal/mol, facilitating the C–H activation reaction under the heating conditions. For comparison, the free energy barrier of the C–H activation reaction of **B** with **CAT** is lowered from 36.5 to 33.7 kcal/mol when the same explicit solvation model is used.

### 2.2. Structural, Energetic, and Wavefunction Analyses

#### 2.2.1. Understanding the Distinctions Between **A** and **B**

**Figure 6:**
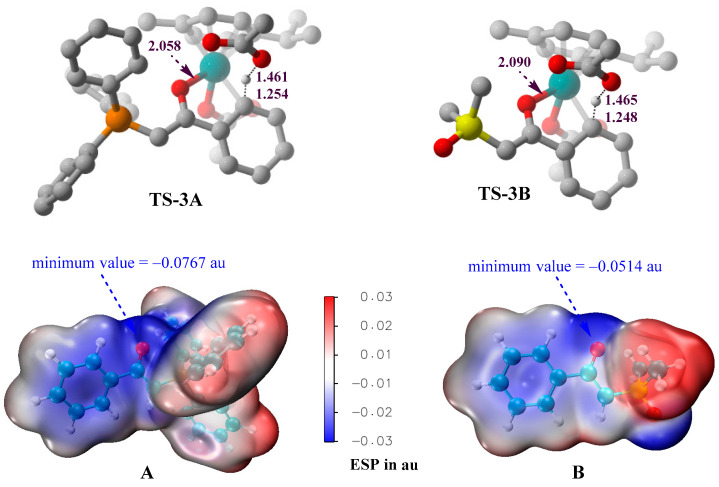
Three-dimensional geometries of **TS-3A** and **TS-3B** (hydrogen atoms, except the transferred one, are omitted for simplicity), and the ESP surfaces of **A** and **B**, generated at the B3LYP+D3BJ/def2SVP level.

In order to explain why phosphonium ylide **A** is a better C–H activation substrate than sulfoxonium ylide **B**, the three-dimensional geometries of the proton-transfer transition states (**TS-3A** and **TS-3B**) and the molecular electrostatic potential (ESP) surfaces of the ylide reagents are provided in Figure 6. Although the C–H and O–H distances in **TS-3A** and **TS-3B** are similar, the Ru–O coordination bond between the ylide and the catalyst in **TS-3A** is shorter than that in **TS-3B**, indicating that the carbonyl oxygen atom of **A** is a better coordination center than that of **B**. ESP surfaces provide deeper insights into the different electronic properties of the carbonyl groups of **A** and **B**. The presence of the electron-withdrawing -SOMe_2_ group in **B** would diminish the negative electrostatic region around the carbonyl oxygen, as compared to the situation in **A**. The minimum value of ESP around the carbonyl oxygen of **A** is −0.0767 au, which is more negative than the corresponding value (−0.0514 au) of **B**. The ESP results support that the carbonyl group in phosphonium ylide **A** is a better directing group of C–H activation than that in sulfoxonium ylide **B**.

**Figure 7:**
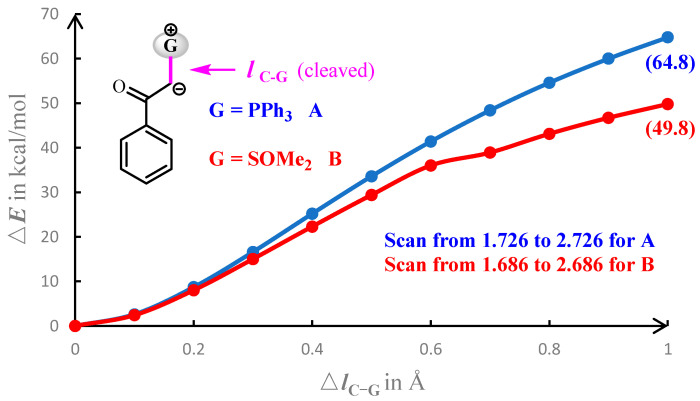
Curves of the relaxed potential energy surface scan from the equilibrium bond length for reactant **A** (in blue) and **B** (in red).

The fact that sulfoxonium ylide **B** has a higher reactivity towards the insertion reaction with **INT-4A** may reflect that -SOMe_2_ is a better leaving group than -PPh_3_. In this regard, we have performed the relaxed potential energy surface scan on the reactant by gradually lengthening the C–G bond of **A** or **B** by 1.0 Å, as shown in Figure 7. During the lengthening of the C–G bond, the energetic increase in the **A** system is larger than that in the **B** system. The lengthening of the C–G bond by 1.0 Å results in an energetic increase of 64.8 or 49.6 kcal/mol for the **A** or **B** systems, respectively. The results of the potential energy scan demonstrate that the -SOMe_2_ group of **B** is more labile than the -PPh_3_ group of **A** in the insertion reaction with ruthenium.

#### 2.2.2. Electronic Properties of Ruthenium–Carbene Intermediate

**Figure 8:**
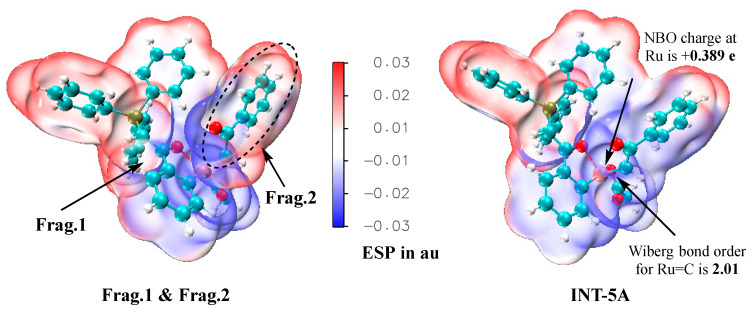
ESP surfaces of the overlay of two fragments (**left**) and **INT-5A** as a whole (**right**), generated at the B3LYP+D3BJ/def2SVP level.

The ruthenium–carbene species **INT-5A** is a key intermediate in the catalytic cycles. We have confirmed that the closed-shell state is the most stable for **INT-5A** because the located triplet state of **INT-5A** has a free energy that is 12.0 kcal/mol greater than that of the closed-shell one, and the possible open-shell singlet state has not been successfully located. Then, a series of wavefunction analyses based on the Molecular Electron Density Theory [31,32] were performed to probe the electronic properties of this special species. In these analyses, **INT-5A** is considered to be composed of the ruthenium complex fragment and the carbene fragment, which are named Frag. 1 and Frag. 2, respectively. Firstly, the ESP surfaces are illustrated in Figure 8, in which the left graph corresponds to the overlay of the ESP surfaces of two separate fragments, and the right one is the graph of the ESP surfaces of **INT-5A** as a whole. Electrostatically, **INT-5A** can be well described by the combination of these two fragments because the two ESP graphs in Figure 8 are generally similar. The Wiberg bond order between the ruthenium center and the carbene carbon is 2.01, supporting the double-bond character of Ru=C, as shown in the Lewis structure. The analytical results based on the Atoms in Molecules theory [33] show that the electron density value at the bond critical point of the Ru=C bond is 0.192 au, which is substantially larger than the corresponding value (0.130 au) of the Ru–C bond in **INT-5A**, demonstrating that the Ru=C interaction is much stronger than the Ru–C one. The natural bond orbital (NBO) charge at the ruthenium center of **INT-5A** is only +0.389 *e*, indicating that the ruthenium–carbene species is a Ru(II) complex instead of a Ru(IV) one. Another piece of evidence that supports that **INT-5A** is a Ru(II) complex is the observation that the Ru–O and Ru–C single bond lengths in **INT-5A** are close to those in **INT-4A** (i.e., 2.001 and 1.982 Å vs. 2.081 and 1.965 Å).

**Figure 9:**
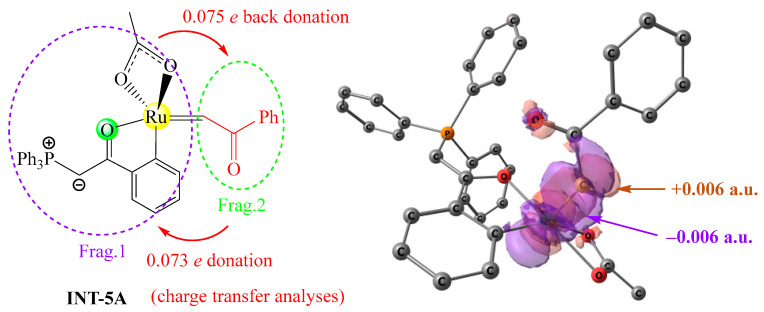
Charge decomposition analyses of **INT-5A** (**left**) and maps of electron density difference between **INT-5A** and two fragments (**right**, Δ*ρ* = *ρ*_INT-5A_ − *ρ*_Frag.1_ − *ρ*_Frag.2_), generated at the B3LYP+D3BJ/def2SVP level.

Secondly, charge decomposition analyses were performed on **INT-5A** to probe the nature of the charge transfer interaction between two fragments, as shown in Figure 9. The degree of electron donation from the carbene fragment to the ruthenium fragment is 0.073 e, and the degree of back donation from the ruthenium fragment to the carbene fragment is 0.075 e, indicating that the nature of the charge transfer interaction in **INT-5A** is bidirectional and the net electron transfer is trivial. To deeply understand the bidirectional charge transfers in **INT-5A**, maps of the electron density difference between **INT-5A** and the two fragments are plotted, in which tangerine surfaces represent the areas that gain electron density upon the formation of **INT-5A** from the two fragments, and purple surfaces represent the areas that lose electron density as a result of this process. There are a large number of tangerine and purple surfaces around the ruthenium center and the carbene carbon, indicating that the carbene fragment is both an electron donor and acceptor.

Lastly, orbital interaction diagrams were obtained from the charge decomposition analysis results to further elucidate the nature of the Ru=C bonding interaction [34]. The main orbital interaction diagrams of the two fragments are provided in the Supplementary Materials. The electron donation from Frag. 2 to Frag. 1 is mainly contributed by the interaction between the lone pair orbital at the carbene center and a ruthenium center empty orbital, and the back donation from Frag. 1 to Frag. 2 is mainly the result of the interaction between a ruthenium center occupied orbital and the empty 2*p* orbital at the carbene center. Therefore, the Ru=C bond can be described by the combination of a *σ*-type coordination bond and a *π*-type backbonding interaction.

## 3. Materials and Methods

All DFT calculations were completed using the Gaussian 09 computational program [35]. Molecular geometries were fully optimized by the use of the B3LYP functional method [36,37,38] with Grimme’s D3BJ dispersion [39,40] and the def2SVP basis sets [41,42] in the gas phase, denoted as B3LYP+D3BJ/def2SVP in this paper. Vibrational analyses under the conditions of 393.15 K and 1 atm were carried out at the same level of theory. The contributions of small vibrational frequencies to Gibbs free energies were corrected by using Grimme’s quasi-RRHO approach [43]. For changing the standard state of the reactant from 24.5 L/mol (in gas) to 1 mol/L (in solution), corrections of ±2.5 kcal/mol (±*RT*ln24.5) were made for reaction steps involving two species to one species or one species to two species in Gibbs free energy determinations. For some suspected transition states, intrinsic reaction coordinate computations [44,45] were performed to confirm their connectivity. In order to further refine the electronic energies obtained, single-point calculations were performed using the B3LYP+D3BJ functional method and the def2TZVPP basis sets [41,42] in ethanol solvent, in which the default IEF-PCM solvation method [46] was used. Wavefunction analyses, including molecular EPS analyses [47], charge decomposition analyses [48], and maps of electron density difference, were performed using the Multiwfn_3.7 program [49,50]. The three-dimensional molecular geometries were made using the CYLView [51] software (Version 1.0b).

## 4. Conclusions

Density functional theory calculations were performed to address the mechanistic issues of a ruthenium-catalyzed dehydrogenative annulation between phosphonium ylides **A** and sulfoxonium ylides **B**. The following main conclusions were reached:

1. The plausible catalytic cycles consist of several elementary steps, including C–H activation, ylide insertion, reductive elimination, protodemetallation, and an intramolecular Wittig reaction in succession, among which C–H activation is rate-determining, with a free energy barrier of 31.7 kcal/mol.
2. In the proposed catalytic cycles, **A** serves as the substrate in the C–H activation step, and **B** is used as the carbene precursor in the insertion step. A comparison of the reactivities of **A** and **B** during the different steps resulted in the conclusion that **A** is kinetically more favorable in the C–H activation step than **B**, while the opposite conclusion is true for the insertion step. Therefore, it is understandable that the other competing pathways, including the homo-coupling reactions, can be effectively suppressed.
3. Structural, energetic, and electronic analyses were carried out to deeply understand the observed results. The carbonyl group of **A** is a better directing group of C–H activation than that of **B**, while the -SOMe_2_ group of **B** is more labile than the -PPh_3_ group of **A** in the insertion step.

## Data Availability

Data are contained within the article and Supplementary Materials.

## Supplementary Materials

The following supporting information can be downloaded at: https://www.mdpi.com/article/10.3390/molecules30091883/s1. Table S1: Cartesian coordinates of the optimized stationary points in the C-H activation pathways; Table S2: Cartesian coordinates of the optimized stationary points in the remaining steps after C-H activation; Table S3: Imaginary frequency of all the transition states; Table S4: Electronic energies (*E*) and Gibbs free energies (*G*) calculated at the B3LYP+D3BJ/def2SVP level and single-point energies (*E*′) calculated at the B3LYP+D3BJ-PCM/def2TZVPP level; Figure S1: Energetics for the generation of the active catalyst; Figure S2: Orbital interaction diagrams between two fragments in INT-5A.
