# Supplementary material for: Comparison of Phosphonium and Sulfoxonium Ylides in Ru(II)-Catalyzed Dehydrogenative Annulations: A Density Functional Theory Study
Source: Molecules. 2025 Apr 23;30(9):1883. doi: 10.3390/molecules30091883 (PMC12074449; doi:10.3390/molecules30091883)
Supplement: Supplementary file 1 [file molecules-30-01883-s001.zip › molecules-3535158-supplementary.pdf]

Table S1. Cartesian Coordinates of the optimized stationary points in the C-H activation pathways, calculated at the B3LYP+D3BJ/def2SVP level

Names	Cartesian Coordinates			
CAT	C	1.646611000	0.044038000	0.997378000
	C	1.909255000	-0.424150000	-0.311297000
	C	1.339477000	-1.689176000	-0.693410000
	C	0.520257000	-2.426988000	0.186858000
	C	0.196946000	-1.900856000	1.483319000
	C	0.793858000	-0.677154000	1.887551000
	H	1.953907000	1.042333000	1.299396000
	H	1.456596000	-2.030232000	-1.724030000
	H	0.021752000	-3.330853000	-0.167040000
	H	0.502220000	-0.203339000	2.823423000
	C	-0.812484000	-2.596216000	2.350219000
	H	-1.255663000	-1.895936000	3.071395000
	H	-1.622350000	-3.020943000	1.740064000
	H	-0.333621000	-3.416402000	2.910996000
	C	2.706359000	0.393742000	-1.309252000
	H	2.493607000	-0.035499000	-2.302730000
	C	4.209268000	0.236457000	-1.031674000
	H	4.513162000	-0.822180000	-1.033551000
	H	4.803153000	0.763850000	-1.794523000
	H	4.468842000	0.660055000	-0.047778000
	C	2.279245000	1.865820000	-1.342613000
	H	2.827698000	2.400271000	-2.133602000
	H	1.200910000	1.957444000	-1.538241000
	H	2.493225000	2.375531000	-0.390184000
	Ru	-0.253419000	-0.380572000	-0.000451000
	C	-2.263828000	-0.398682000	-1.509854000
	O	-2.321574000	-0.872286000	-0.333385000
	O	-1.121096000	-0.062409000	-1.947376000
	C	-3.502152000	-0.195430000	-2.329952000
	C	-0.766292000	2.337563000	1.173534000
	O	0.081484000	2.202888000	2.047769000
	O	-1.080950000	1.459823000	0.261159000
	C	-1.590039000	3.612535000	1.052640000
	H	-3.273700000	-0.279970000	-3.400803000
	H	-4.281127000	-0.912072000	-2.038037000
	H	-3.875378000	0.823134000	-2.135703000
	H	-2.658721000	3.375225000	1.170586000
	H	-1.464427000	4.044190000	0.047724000
	H	-1.281434000	4.337383000	1.816456000
A	C	2.033360000	-0.200985000	-0.240560000

	O	1.788683000	-1.428879000	-0.218061000
	C	3.473805000	0.247333000	-0.258249000
	C	3.870914000	1.589282000	-0.365447000
	C	4.462740000	-0.743702000	-0.161426000
	C	5.224519000	1.931633000	-0.371284000
	H	3.121506000	2.377471000	-0.452426000
	C	5.815569000	-0.404083000	-0.164422000
	H	4.129277000	-1.779791000	-0.084460000
	C	6.201195000	0.936208000	-0.268814000
	H	5.519423000	2.980573000	-0.457525000
	H	6.574848000	-1.186607000	-0.086159000
	H	7.260545000	1.205419000	-0.272471000
	C	0.995086000	0.761887000	-0.216625000
	H	1.133815000	1.839588000	-0.190120000
	P	-0.609781000	0.149709000	-0.046038000
	C	-1.697643000	1.590390000	0.198078000
	C	-2.638493000	1.634292000	1.236034000
	C	-1.595364000	2.672794000	-0.692721000
	C	-3.464088000	2.752457000	1.384921000
	H	-2.724198000	0.797116000	1.930722000
	C	-2.421588000	3.786366000	-0.539264000
	H	-0.870844000	2.637092000	-1.509143000
	C	-3.357246000	3.827803000	0.500019000
	H	-4.192510000	2.782045000	2.198518000
	H	-2.336502000	4.624779000	-1.234463000
	H	-4.003771000	4.700511000	0.618805000
	C	-0.835790000	-0.929303000	1.406123000
	C	-1.846972000	-1.898742000	1.445450000
	C	-0.002158000	-0.739124000	2.515991000
	C	-2.028344000	-2.667855000	2.597694000
	H	-2.486004000	-2.058458000	0.574833000
	C	-0.185566000	-1.511331000	3.663167000
	H	0.793710000	0.007048000	2.462447000
	C	-1.199254000	-2.474415000	3.706178000
	H	-2.815202000	-3.425273000	2.626455000
	H	0.470772000	-1.368122000	4.524700000
	H	-1.338170000	-3.081340000	4.604134000
	C	-1.337196000	-0.775954000	-1.448315000
	C	-0.639229000	-1.874425000	-1.983900000
	C	-2.582483000	-0.411474000	-1.984652000
	C	-1.189724000	-2.587524000	-3.049675000
	H	0.325623000	-2.146147000	-1.550551000
	C	-3.125390000	-1.135849000	-3.049496000
	H	-3.133461000	0.436238000	-1.574710000

	C	-2.430159000	-2.223040000	-3.583738000
	H	-0.643796000	-3.437827000	-3.465428000
	H	-4.095411000	-0.847101000	-3.461316000
	H	-2.855400000	-2.787650000	-4.417191000
B	C	2.113710000	1.215933000	0.002594000
	C	1.584097000	-0.083665000	0.000980000
	C	2.464530000	-1.176136000	-0.001641000
	C	3.844707000	-0.975833000	-0.003156000
	C	4.364069000	0.322626000	-0.001726000
	C	3.494777000	1.417926000	0.001284000
	H	1.449387000	2.081355000	0.005502000
	H	2.029911000	-2.176907000	-0.002400000
	H	4.520477000	-1.834804000	-0.005393000
	H	5.445255000	0.481956000	-0.002806000
	H	3.894949000	2.434722000	0.002763000
	C	0.108550000	-0.381540000	0.002327000
	O	-0.273960000	-1.573001000	0.006435000
	C	-0.808277000	0.701846000	-0.001310000
	H	-0.616341000	1.770867000	-0.008533000
	S	-2.454927000	0.341909000	-0.001512000
	O	-3.329071000	1.531011000	-0.006437000
	C	-2.847237000	-0.722220000	1.402864000
	H	-2.128434000	-1.552024000	1.383165000
	H	-2.714259000	-0.084683000	2.286110000
	H	-3.889975000	-1.052238000	1.304562000
	C	-2.845713000	-0.732106000	-1.398926000
	H	-3.888389000	-1.061934000	-1.299345000
	H	-2.712293000	-0.100545000	-2.286397000
	H	-2.126492000	-1.561386000	-1.372760000
TS-1A	C	-3.177022000	-2.275921000	0.857489000
	C	-1.769626000	-2.213139000	0.713045000
	C	-1.220603000	-2.161627000	-0.614308000
	C	-2.047538000	-2.099740000	-1.749332000
	C	-3.468130000	-2.009478000	-1.599375000
	C	-4.014176000	-2.124122000	-0.280328000
	H	-3.640957000	-2.223591000	1.838993000
	H	-0.144715000	-2.055072000	-0.728387000
	H	-1.609730000	-1.920216000	-2.727137000
	H	-5.081063000	-1.970298000	-0.118597000
	C	-4.344691000	-1.764817000	-2.792540000
	H	-5.287957000	-1.285071000	-2.494872000
	H	-3.822903000	-1.119198000	-3.511871000
	H	-4.587735000	-2.723399000	-3.282706000
	C	-0.833098000	-2.154735000	1.900895000

	H	0.085527000	-1.682634000	1.527323000
	C	-0.491253000	-3.575965000	2.370547000
	H	-0.091173000	-4.186321000	1.545250000
	H	0.270542000	-3.541624000	3.165235000
	H	-1.382202000	-4.088476000	2.769150000
	C	-1.362144000	-1.275283000	3.035892000
	H	-0.623967000	-1.229490000	3.850347000
	H	-1.539085000	-0.253372000	2.672605000
	H	-2.297141000	-1.665357000	3.467340000
	Ru	-2.623945000	-0.503226000	-0.332105000
	C	-1.789022000	1.198192000	-2.675357000
	O	-2.261413000	1.119347000	-1.464960000
	O	-1.812984000	0.305816000	-3.519268000
	C	-1.170583000	2.556888000	-2.964789000
	C	-4.504202000	0.707919000	1.672958000
	O	-5.108063000	-0.334631000	1.880077000
	O	-3.572970000	0.888617000	0.769168000
	C	-4.787871000	1.975645000	2.463972000
	H	-0.823017000	2.611334000	-4.004436000
	H	-1.906221000	3.350403000	-2.765860000
	H	-0.334559000	2.733495000	-2.269995000
	H	-5.168604000	2.753333000	1.783464000
	H	-3.855264000	2.361784000	2.902956000
	H	-5.526765000	1.776929000	3.250472000
	C	0.651954000	1.733281000	0.506460000
	O	-0.022550000	0.683657000	0.677040000
	C	-0.005658000	3.084269000	0.626232000
	C	0.719254000	4.289570000	0.594328000
	C	-1.398261000	3.143295000	0.781602000
	C	0.068854000	5.518725000	0.705212000
	H	1.804842000	4.278861000	0.492443000
	C	-2.049050000	4.373348000	0.888667000
	H	-1.976930000	2.224765000	0.790543000
	C	-1.321604000	5.565319000	0.850757000
	H	0.649753000	6.444197000	0.680126000
	H	-3.136021000	4.393194000	0.997214000
	H	-1.832495000	6.528134000	0.934417000
	C	2.035964000	1.687107000	0.206866000
	H	2.649500000	2.564336000	0.021301000
	P	2.842210000	0.176056000	0.042055000
	C	4.545606000	0.540694000	-0.491060000
	C	5.024110000	0.160451000	-1.751482000
	C	5.378722000	1.254350000	0.386831000
	C	6.327661000	0.494007000	-2.131308000

	H	4.379475000	-0.391257000	-2.437428000
	C	6.677193000	1.586914000	0.001122000
	H	5.008307000	1.546193000	1.372281000
	C	7.153092000	1.206311000	-1.258510000
	H	6.696619000	0.197068000	-3.115654000
	H	7.321340000	2.143212000	0.685890000
	H	8.170863000	1.466221000	-1.558878000
	C	2.147787000	-0.928452000	-1.229999000
	C	2.413830000	-2.306266000	-1.249058000
	C	1.396798000	-0.346347000	-2.260275000
	C	1.947275000	-3.088258000	-2.307944000
	H	2.972598000	-2.772657000	-0.436888000
	C	0.929032000	-1.131536000	-3.315915000
	H	1.169858000	0.720011000	-2.219121000
	C	1.211640000	-2.500483000	-3.342750000
	H	2.152169000	-4.161217000	-2.320379000
	H	0.304268000	-0.670782000	-4.082381000
	H	0.840439000	-3.116828000	-4.165043000
	C	3.042271000	-0.801741000	1.572987000
	C	2.168684000	-0.587958000	2.649306000
	C	4.091992000	-1.726729000	1.697492000
	C	2.339554000	-1.309301000	3.833003000
	H	1.354672000	0.129597000	2.545993000
	C	4.251449000	-2.449914000	2.881193000
	H	4.800119000	-1.867473000	0.877843000
	C	3.373285000	-2.242995000	3.949249000
	H	1.660654000	-1.138372000	4.671150000
	H	5.070357000	-3.167047000	2.973786000
	H	3.502000000	-2.803521000	4.878201000
INT-1A	C	2.637242000	-2.747481000	0.018644000
	C	1.325981000	-2.663594000	0.525248000
	C	1.120693000	-1.798964000	1.655048000
	C	2.179722000	-1.153925000	2.321647000
	C	3.510892000	-1.258301000	1.798096000
	C	3.724907000	-2.053016000	0.641466000
	H	2.819134000	-3.276022000	-0.915308000
	H	0.103895000	-1.615934000	1.988816000
	H	1.965655000	-0.455422000	3.127572000
	H	4.710083000	-2.065606000	0.173728000
	C	4.634877000	-0.471581000	2.406560000
	H	5.325083000	-0.113413000	1.630850000
	H	4.251028000	0.398823000	2.954489000
	H	5.197149000	-1.105147000	3.112999000
	C	0.138955000	-3.406699000	-0.057794000

	H	-0.612382000	-2.642514000	-0.306939000
	C	-0.466939000	-4.333847000	1.010062000
	H	-0.750115000	-3.787983000	1.921851000
	H	-1.371719000	-4.823414000	0.616318000
	H	0.248725000	-5.122781000	1.294859000
	C	0.459959000	-4.186355000	-1.330891000
	H	-0.458388000	-4.646553000	-1.725009000
	H	0.871104000	-3.520518000	-2.099899000
	H	1.176865000	-5.000048000	-1.126538000
	Ru	2.202387000	-0.582679000	0.200794000
	C	2.548404000	2.087141000	1.463969000
	O	2.986816000	1.298265000	0.533404000
	O	1.606602000	1.862054000	2.225763000
	C	3.329666000	3.390488000	1.544485000
	C	2.325944000	-0.756917000	-2.790470000
	O	1.369718000	-1.533319000	-2.799862000
	O	2.879489000	-0.239634000	-1.742677000
	C	2.935638000	-0.267482000	-4.099475000
	H	2.973827000	3.994168000	2.389119000
	H	4.404679000	3.179655000	1.651739000
	H	3.200517000	3.950033000	0.607281000
	H	4.014493000	-0.086988000	-3.996161000
	H	2.459821000	0.691425000	-4.365527000
	H	2.733896000	-0.986319000	-4.904930000
	C	-0.069236000	1.563911000	-0.619760000
	O	0.415311000	0.400441000	-0.399017000
	C	0.746900000	2.772681000	-0.959988000
	C	0.333016000	4.041566000	-0.517215000
	C	1.892605000	2.677389000	-1.762521000
	C	1.027466000	5.191373000	-0.894870000
	H	-0.526744000	4.122388000	0.149735000
	C	2.576925000	3.829527000	-2.152029000
	H	2.263751000	1.696315000	-2.046035000
	C	2.145587000	5.089643000	-1.727817000
	H	0.698841000	6.168179000	-0.531723000
	H	3.465364000	3.736907000	-2.781238000
	H	2.689003000	5.988610000	-2.029776000
	C	-1.457429000	1.737339000	-0.588182000
	H	-1.942077000	2.665127000	-0.882475000
	P	-2.490199000	0.450628000	-0.055586000
	C	-4.166546000	1.146238000	0.087040000
	C	-4.725631000	1.452338000	1.334965000
	C	-4.879065000	1.433564000	-1.088752000
	C	-5.991938000	2.039607000	1.405224000

	H	-4.172816000	1.232196000	2.249733000
	C	-6.141411000	2.022685000	-1.012113000
	H	-4.447140000	1.189741000	-2.061988000
	C	-6.699326000	2.324621000	0.234788000
	H	-6.425684000	2.275223000	2.379658000
	H	-6.693068000	2.244670000	-1.928447000
	H	-7.689493000	2.782728000	0.292829000
	C	-2.043177000	-0.159163000	1.599608000
	C	-2.576344000	-1.357433000	2.100323000
	C	-1.202177000	0.625857000	2.400216000
	C	-2.279981000	-1.756965000	3.404520000
	H	-3.207340000	-1.987220000	1.471354000
	C	-0.903871000	0.215199000	3.701443000
	H	-0.732124000	1.526760000	2.008822000
	C	-1.446585000	-0.968369000	4.207585000
	H	-2.692037000	-2.691560000	3.791603000
	H	-0.220396000	0.823242000	4.296894000
	H	-1.208532000	-1.290167000	5.224275000
	C	-2.649415000	-0.953928000	-1.201099000
	C	-1.628722000	-1.190802000	-2.132949000
	C	-3.784742000	-1.782713000	-1.173675000
	C	-1.742453000	-2.254002000	-3.030956000
	H	-0.723387000	-0.587154000	-2.141092000
	C	-3.886687000	-2.848337000	-2.069228000
	H	-4.597393000	-1.584167000	-0.471130000
	C	-2.866298000	-3.082275000	-2.998958000
	H	-0.921022000	-2.426865000	-3.727546000
	H	-4.769518000	-3.491722000	-2.048441000
	H	-2.951499000	-3.916764000	-3.699500000
TS-2A	Ru	1.848523000	-0.224529000	-0.643335000
	C	1.794789000	-3.139032000	-0.125624000
	O	1.229718000	-3.327842000	-1.200348000
	O	2.147577000	-1.977991000	0.339969000
	C	2.135117000	-4.279009000	0.819526000
	H	3.217444000	-4.286614000	1.020157000
	H	1.634990000	-4.111265000	1.785378000
	H	1.824558000	-5.239484000	0.389335000
	C	-0.459122000	-0.568115000	1.364526000
	O	-0.008212000	-0.171450000	0.217173000
	C	0.455154000	-0.986477000	2.458257000
	C	0.154694000	-2.104009000	3.251414000
	C	1.634189000	-0.262717000	2.693754000
	C	1.038591000	-2.510911000	4.252538000
	H	-0.756536000	-2.674769000	3.058461000

	C	2.508574000	-0.668079000	3.701022000
	C	2.219089000	-1.795067000	4.476626000
	H	0.809881000	-3.393930000	4.854432000
	H	3.423824000	-0.098081000	3.876781000
	H	2.911729000	-2.115955000	5.258810000
	C	-1.832091000	-0.561793000	1.554107000
	H	-2.304018000	-0.794457000	2.506428000
	C	3.163495000	-0.816330000	-2.255346000
	C	1.829295000	-0.609158000	-2.722730000
	C	1.173842000	0.657058000	-2.539262000
	C	1.856498000	1.664557000	-1.816393000
	C	3.134824000	1.406742000	-1.256598000
	C	3.829759000	0.177781000	-1.498546000
	H	3.596734000	-1.814252000	-2.339745000
	H	1.287782000	-1.443252000	-3.169246000
	H	1.330487000	2.571339000	-1.522332000
	H	3.528796000	2.118838000	-0.512895000
	C	-0.232918000	0.858368000	-3.013977000
	H	-0.782453000	1.532361000	-2.344982000
	H	-0.220512000	1.306637000	-4.021859000
	H	-0.768753000	-0.095399000	-3.073032000
	C	5.169621000	-0.082459000	-0.835117000
	C	5.078879000	0.009364000	0.696675000
	C	6.223530000	0.883788000	-1.395881000
	H	5.463544000	-1.111390000	-1.104544000
	H	4.376393000	-0.740153000	1.090386000
	H	6.069512000	-0.178975000	1.140696000
	H	4.740264000	1.008515000	1.015274000
	H	6.285372000	0.826417000	-2.494468000
	H	5.983890000	1.922088000	-1.116878000
	H	7.217348000	0.647685000	-0.984593000
	H	1.831860000	0.637187000	2.101327000
	C	2.422027000	3.153461000	1.451619000
	O	3.628149000	3.108323000	1.127396000
	O	1.518954000	2.335347000	1.091876000
	C	1.964924000	4.326914000	2.332733000
	H	1.657533000	5.163493000	1.680870000
	H	1.101325000	4.044329000	2.952262000
	H	2.793128000	4.684684000	2.960869000
	P	-2.843028000	-0.003895000	0.242654000
	C	-2.671916000	-1.005374000	-1.269603000
	C	-3.430503000	-0.702794000	-2.413112000
	C	-1.763398000	-2.070647000	-1.299966000
	C	-3.273757000	-1.461048000	-3.573097000

	C	-1.592767000	-2.814566000	-2.470174000
	C	-2.349565000	-2.512921000	-3.604583000
	H	-4.138494000	0.126714000	-2.406853000
	H	-1.157965000	-2.311009000	-0.429813000
	H	-3.867172000	-1.223708000	-4.459034000
	H	-0.840423000	-3.604671000	-2.471183000
	H	-2.217500000	-3.093534000	-4.520853000
	C	-2.607064000	1.754574000	-0.179465000
	C	-3.595093000	2.431892000	-0.917376000
	C	-1.443709000	2.432052000	0.217421000
	C	-3.406257000	3.765899000	-1.279162000
	C	-1.265708000	3.768709000	-0.149229000
	C	-2.236393000	4.434676000	-0.900179000
	H	-4.525045000	1.929451000	-1.191309000
	H	-0.640643000	1.960055000	0.782162000
	H	-4.177999000	4.286738000	-1.850709000
	H	-0.346194000	4.265060000	0.166571000
	H	-2.090025000	5.479451000	-1.185244000
	C	-4.568142000	-0.160505000	0.794254000
	C	-5.093880000	0.806485000	1.666368000
	C	-5.343224000	-1.270622000	0.430762000
	C	-6.389121000	0.662799000	2.165177000
	C	-6.640285000	-1.407025000	0.931696000
	C	-7.163258000	-0.442444000	1.796975000
	H	-4.491413000	1.672593000	1.948816000
	H	-4.934674000	-2.025282000	-0.243626000
	H	-6.796135000	1.417265000	2.842081000
	H	-7.243449000	-2.271182000	0.644401000
	H	-8.178389000	-0.551036000	2.185929000
INT-2A	Ru	1.937828000	0.043164000	-0.549839000
	C	2.410110000	-2.877522000	-0.325874000
	O	3.608664000	-2.660985000	-0.199977000
	O	1.488105000	-1.970126000	-0.492372000
	C	1.845619000	-4.290184000	-0.263807000
	H	1.320374000	-4.418121000	0.695350000
	H	1.111244000	-4.456072000	-1.066349000
	H	2.657124000	-5.025930000	-0.329982000
	C	-0.371178000	-0.459412000	1.220773000
	O	0.034222000	0.251727000	0.219487000
	C	0.671768000	-1.060676000	2.088429000
	C	0.491962000	-2.314914000	2.687553000
	C	1.917774000	-0.408312000	2.190035000
	C	1.551313000	-2.930429000	3.353862000
	H	-0.463306000	-2.832395000	2.581308000

	C	2.973942000	-1.039882000	2.856951000
	C	2.798556000	-2.299602000	3.429329000
	H	1.412060000	-3.919307000	3.797600000
	H	3.935990000	-0.532638000	2.935504000
	H	3.631097000	-2.792880000	3.935733000
	C	-1.725371000	-0.674245000	1.414442000
	H	-2.117559000	-1.180593000	2.294290000
	P	-2.873380000	-0.186081000	0.188848000
	C	-4.447057000	-0.988819000	0.615777000
	C	-5.003580000	-1.990582000	-0.190084000
	C	-5.089606000	-0.608685000	1.806433000
	C	-6.197921000	-2.607502000	0.193100000
	H	-4.505952000	-2.288823000	-1.114266000
	C	-6.279730000	-1.230292000	2.183996000
	H	-4.662249000	0.178773000	2.431831000
	C	-6.835189000	-2.229476000	1.377055000
	H	-6.630066000	-3.387669000	-0.437528000
	H	-6.777346000	-0.932149000	3.109481000
	H	-7.768760000	-2.713350000	1.673387000
	C	-2.400084000	-0.755101000	-1.473902000
	C	-3.045328000	-0.218560000	-2.599525000
	C	-1.412438000	-1.735629000	-1.628986000
	C	-2.716185000	-0.680461000	-3.874614000
	H	-3.793488000	0.567830000	-2.481439000
	C	-1.073071000	-2.176707000	-2.909803000
	H	-0.870740000	-2.126409000	-0.768796000
	C	-1.728381000	-1.659690000	-4.030748000
	H	-3.221193000	-0.264657000	-4.749309000
	H	-0.281621000	-2.920494000	-3.022390000
	H	-1.462293000	-2.010177000	-5.030929000
	C	-3.215090000	1.592096000	0.072196000
	C	-2.159243000	2.497778000	0.253806000
	C	-4.505458000	2.062099000	-0.231821000
	C	-2.391438000	3.869840000	0.136781000
	H	-1.146411000	2.166221000	0.477624000
	C	-4.726875000	3.435113000	-0.354703000
	H	-5.334345000	1.363039000	-0.361341000
	C	-3.672062000	4.337284000	-0.169675000
	H	-1.534741000	4.526827000	0.304215000
	H	-5.729451000	3.801844000	-0.587946000
	H	-3.855609000	5.410953000	-0.260275000
	C	3.559307000	-0.150559000	-1.994385000
	C	2.314054000	0.147216000	-2.628065000
	C	1.544233000	1.296535000	-2.249056000

	C	2.059514000	2.151573000	-1.226778000
	C	3.254175000	1.811012000	-0.566456000
	C	4.030145000	0.657639000	-0.930383000
	H	4.060587000	-1.093200000	-2.210933000
	H	1.893677000	-0.562069000	-3.343688000
	H	1.451600000	2.949110000	-0.768349000
	H	3.454671000	2.371271000	0.349564000
	C	0.188421000	1.549089000	-2.837236000
	H	-0.485964000	1.969931000	-2.079788000
	H	0.271036000	2.277182000	-3.661726000
	H	-0.255321000	0.626796000	-3.232089000
	C	5.268825000	0.236078000	-0.158747000
	C	5.506476000	1.065355000	1.106007000
	C	6.500600000	0.288634000	-1.078582000
	H	5.096154000	-0.815257000	0.122465000
	H	4.614318000	1.134112000	1.742998000
	H	6.320587000	0.618457000	1.697057000
	H	5.804177000	2.097166000	0.858951000
	H	6.379119000	-0.351979000	-1.965083000
	H	6.690856000	1.318056000	-1.424910000
	H	7.394974000	-0.055840000	-0.536365000
	H	2.024678000	0.653733000	1.895198000
	C	1.052975000	3.157841000	1.858966000
	O	2.183557000	2.616267000	1.987627000
	O	0.606969000	3.723428000	0.826707000
	C	0.100289000	3.045638000	3.061739000
	H	-0.621402000	3.875216000	3.085388000
	H	-0.473607000	2.106648000	2.964883000
	H	0.662592000	2.994871000	4.005078000
TS-3A	Ru	1.840214000	-0.137527000	-0.486628000
	C	2.248087000	-3.009686000	0.248074000
	O	3.464429000	-2.859578000	0.197240000
	O	1.351451000	-2.122279000	-0.068009000
	C	1.635603000	-4.310586000	0.753294000
	H	1.279844000	-4.146272000	1.783168000
	H	0.765542000	-4.603281000	0.146801000
	H	2.387760000	-5.110008000	0.758056000
	C	-0.470262000	-0.237807000	1.273780000
	O	-0.108120000	0.167908000	0.102722000
	C	0.627997000	-0.592955000	2.193378000
	C	0.455697000	-1.416736000	3.309207000
	C	1.923911000	-0.172543000	1.789736000
	C	1.571064000	-1.857401000	4.024723000
	H	-0.542754000	-1.755995000	3.592668000

	C	3.026675000	-0.644015000	2.521111000
	C	2.858199000	-1.481477000	3.623032000
	H	1.437984000	-2.512978000	4.888988000
	H	4.029644000	-0.337560000	2.227804000
	H	3.729753000	-1.845480000	4.172056000
	C	-1.824244000	-0.371820000	1.552726000
	H	-2.216564000	-0.647581000	2.528862000
	P	-2.955182000	-0.084155000	0.258855000
	C	-4.583951000	-0.655358000	0.828935000
	C	-5.143483000	-1.848341000	0.352117000
	C	-5.254634000	0.089941000	1.812686000
	C	-6.369698000	-2.291206000	0.855311000
	H	-4.621811000	-2.428230000	-0.411184000
	C	-6.477239000	-0.359194000	2.312895000
	H	-4.822070000	1.023514000	2.179733000
	C	-7.035745000	-1.549068000	1.833841000
	H	-6.804795000	-3.220501000	0.480768000
	H	-6.997416000	0.222575000	3.077170000
	H	-7.994677000	-1.897357000	2.224732000
	C	-2.554837000	-1.016790000	-1.256833000
	C	-3.222358000	-0.725285000	-2.456565000
	C	-1.580812000	-2.023583000	-1.222758000
	C	-2.926117000	-1.448740000	-3.612927000
	H	-3.963058000	0.075048000	-2.496195000
	C	-1.275823000	-2.729526000	-2.388229000
	H	-1.019524000	-2.232910000	-0.313314000
	C	-1.949659000	-2.450395000	-3.580397000
	H	-3.449550000	-1.220656000	-4.544171000
	H	-0.493590000	-3.490770000	-2.355806000
	H	-1.708671000	-3.006422000	-4.489758000
	C	-3.159645000	1.663490000	-0.202670000
	C	-2.091340000	2.549417000	0.002054000
	C	-4.362890000	2.125480000	-0.765486000
	C	-2.225799000	3.890044000	-0.368582000
	H	-1.141805000	2.235727000	0.436976000
	C	-4.485604000	3.465836000	-1.134879000
	H	-5.209129000	1.447316000	-0.897586000
	C	-3.415766000	4.347792000	-0.939458000
	H	-1.374178000	4.549130000	-0.189475000
	H	-5.422150000	3.824698000	-1.568510000
	H	-3.518680000	5.397291000	-1.226895000
	C	3.306478000	-0.745130000	-1.981357000
	C	2.053312000	-0.490009000	-2.626529000
	C	1.379449000	0.746054000	-2.442699000

	C	2.001457000	1.741322000	-1.618661000
	C	3.240561000	1.476911000	-0.996781000
	C	3.930937000	0.230344000	-1.167268000
	H	3.733794000	-1.745930000	-2.022357000
	H	1.552482000	-1.294848000	-3.165860000
	H	1.453745000	2.643314000	-1.343076000
	H	3.612127000	2.198985000	-0.275094000
	C	0.004113000	0.982735000	-2.991971000
	H	-0.640419000	1.428559000	-2.221932000
	H	0.053160000	1.682092000	-3.843006000
	H	-0.459108000	0.048252000	-3.332476000
	C	5.236042000	-0.090229000	-0.458607000
	C	5.677279000	0.999793000	0.523381000
	C	6.345787000	-0.367277000	-1.485919000
	H	5.044595000	-1.024704000	0.093297000
	H	4.885216000	1.287873000	1.229075000
	H	6.544449000	0.650665000	1.104692000
	H	5.987520000	1.913709000	-0.010539000
	H	6.080291000	-1.194481000	-2.160988000
	H	6.545229000	0.525017000	-2.102889000
	H	7.282069000	-0.640545000	-0.974353000
	H	2.080684000	1.020136000	1.434406000
	C	1.747486000	3.372470000	1.452030000
	O	2.504913000	2.382527000	1.746673000
	O	0.685873000	3.316119000	0.812895000
	C	2.276806000	4.736309000	1.902450000
	H	2.982720000	5.112239000	1.142340000
	H	1.454011000	5.459018000	1.992230000
	H	2.825071000	4.650627000	2.851137000
INT-3A	C	-2.339584000	1.712152000	-1.461331000
	C	-3.254212000	1.866412000	-0.381999000
	C	-4.205846000	0.809284000	-0.204146000
	C	-4.245157000	-0.315287000	-1.062751000
	C	-3.342341000	-0.411531000	-2.178473000
	C	-2.422930000	0.632183000	-2.400186000
	H	-1.522186000	2.426275000	-1.572674000
	H	-4.868996000	0.837518000	0.662567000
	H	-4.883607000	-1.161999000	-0.820036000
	H	-1.682475000	0.550520000	-3.196659000
	C	-3.340661000	-1.652238000	-3.024553000
	H	-2.365630000	-1.797003000	-3.511483000
	H	-3.564228000	-2.527654000	-2.399923000
	H	-4.113657000	-1.580650000	-3.808690000
	C	-3.214297000	3.041289000	0.575155000

	H	-3.704773000	2.697727000	1.501607000
	C	-4.029473000	4.215487000	0.012117000
	H	-5.063189000	3.912871000	-0.217204000
	H	-4.068948000	5.049945000	0.730737000
	H	-3.577442000	4.592129000	-0.920689000
	C	-1.791405000	3.465963000	0.949982000
	H	-1.816990000	4.256924000	1.715552000
	H	-1.223096000	2.612693000	1.345087000
	H	-1.245839000	3.871851000	0.082341000
	Ru	-2.229572000	-0.064462000	-0.218935000
	C	-2.365788000	-3.066462000	0.072725000
	O	-3.590922000	-3.067978000	0.008758000
	O	-1.583279000	-2.048849000	-0.121161000
	C	-1.594715000	-4.339683000	0.412567000
	H	-1.122891000	-4.217607000	1.400573000
	H	-2.271077000	-5.203890000	0.432888000
	H	-0.784597000	-4.510325000	-0.313732000
	C	0.214075000	-0.083556000	1.326300000
	O	-0.213775000	0.303289000	0.169060000
	C	-0.830118000	-0.319575000	2.334637000
	C	-0.582881000	-0.562804000	3.694323000
	C	-2.150255000	-0.283498000	1.813760000
	C	-1.649504000	-0.773763000	4.565660000
	H	0.442388000	-0.581012000	4.073170000
	C	-3.202910000	-0.524734000	2.708804000
	C	-2.958759000	-0.757616000	4.066052000
	H	-1.466354000	-0.957318000	5.627068000
	H	-4.232239000	-0.561229000	2.345313000
	H	-3.798502000	-0.939924000	4.743043000
	C	1.582818000	-0.261300000	1.523626000
	H	2.007411000	-0.609458000	2.462648000
	P	2.670402000	-0.010388000	0.191963000
	C	4.292762000	-0.647333000	0.707581000
	C	4.942871000	-1.662023000	-0.006862000
	C	4.886092000	-0.117679000	1.866451000
	C	6.178441000	-2.143586000	0.435746000
	H	4.482657000	-2.077476000	-0.904788000
	C	6.118600000	-0.602835000	2.303140000
	H	4.383912000	0.677555000	2.422320000
	C	6.766200000	-1.616113000	1.587633000
	H	6.680734000	-2.936705000	-0.122638000
	H	6.576364000	-0.189129000	3.204552000
	H	7.731719000	-1.994834000	1.931046000
	C	2.940773000	1.741819000	-0.260480000

	C	4.226960000	2.250381000	-0.493905000
	C	1.821647000	2.585312000	-0.377854000
	C	4.394566000	3.594909000	-0.839224000
	H	5.100621000	1.602761000	-0.400904000
	C	1.999789000	3.925911000	-0.718982000
	H	0.825534000	2.175408000	-0.205068000
	C	3.283167000	4.433388000	-0.950366000
	H	5.398612000	3.986863000	-1.017821000
	H	1.128723000	4.580226000	-0.802368000
	H	3.416372000	5.484824000	-1.216059000
	C	2.193345000	-0.839505000	-1.353151000
	C	1.203417000	-1.829665000	-1.336843000
	C	2.801062000	-0.463679000	-2.562472000
	C	0.820885000	-2.438068000	-2.534137000
	H	0.679219000	-2.089578000	-0.418004000
	C	2.428688000	-1.093414000	-3.750723000
	H	3.553795000	0.327508000	-2.578968000
	C	1.434998000	-2.079263000	-3.736764000
	H	0.020437000	-3.180195000	-2.516949000
	H	2.903986000	-0.804281000	-4.690947000
	H	1.131284000	-2.558543000	-4.670820000
TS-4A	C	2.058916000	-1.305158000	-1.537079000
	C	3.204050000	-1.559444000	-0.724723000
	C	4.392272000	-0.781767000	-1.001415000
	C	4.335293000	0.317863000	-1.818842000
	C	3.099497000	0.699311000	-2.466773000
	C	2.026811000	-0.186265000	-2.432829000
	H	1.204291000	-1.979603000	-1.515478000
	H	5.320714000	-1.027825000	-0.480203000
	H	5.201333000	0.973798000	-1.918515000
	H	1.115533000	0.022650000	-2.996673000
	C	3.034425000	2.006336000	-3.204606000
	H	2.027478000	2.194377000	-3.603366000
	H	3.299551000	2.829748000	-2.522550000
	H	3.754800000	2.028477000	-4.040035000
	C	3.328895000	-2.813829000	0.128254000
	H	3.872484000	-2.513400000	1.040749000
	C	4.168303000	-3.877037000	-0.599596000
	H	5.156982000	-3.489463000	-0.889590000
	H	4.324914000	-4.761382000	0.039164000
	H	3.658442000	-4.208277000	-1.519760000
	C	1.985409000	-3.396357000	0.572056000
	H	2.145731000	-4.229791000	1.273211000
	H	1.370869000	-2.638332000	1.074310000

	H	1.416395000	-3.796426000	-0.283885000
	Ru	2.066115000	0.248579000	-0.052440000
	C	2.727782000	2.750005000	0.710136000
	O	3.765800000	2.163922000	0.357143000
	O	1.576767000	2.186854000	0.616761000
	C	2.767436000	4.139877000	1.308456000
	H	3.648399000	4.687964000	0.948106000
	H	1.845241000	4.695106000	1.085557000
	H	2.845093000	4.042844000	2.403769000
	C	-0.324411000	-0.265140000	1.404920000
	O	0.112898000	-0.272107000	0.182047000
	C	0.719697000	-0.330097000	2.428418000
	C	0.510807000	-0.546298000	3.798934000
	C	2.027665000	-0.185627000	1.888037000
	C	1.605200000	-0.633840000	4.656809000
	H	-0.503975000	-0.654906000	4.191904000
	C	3.113427000	-0.260650000	2.777115000
	C	2.902951000	-0.489783000	4.139589000
	H	1.455608000	-0.809982000	5.724644000
	H	4.129274000	-0.117811000	2.399725000
	H	3.760977000	-0.549844000	4.816001000
	C	-1.702629000	-0.183568000	1.612792000
	H	-2.159434000	-0.158233000	2.599703000
	P	-2.725773000	-0.023994000	0.219121000
	C	-4.371612000	0.488293000	0.791595000
	C	-4.934242000	1.706892000	0.389783000
	C	-5.072750000	-0.349366000	1.675557000
	C	-6.190208000	2.086197000	0.872049000
	H	-4.389607000	2.358327000	-0.296007000
	C	-6.324659000	0.036015000	2.154883000
	H	-4.639303000	-1.304050000	1.982976000
	C	-6.884499000	1.253916000	1.753129000
	H	-6.625375000	3.037658000	0.558194000
	H	-6.866894000	-0.616389000	2.843081000
	H	-7.865764000	1.553212000	2.128806000
	C	-2.986733000	-1.578554000	-0.707709000
	C	-4.223954000	-1.882237000	-1.294879000
	C	-1.913226000	-2.476106000	-0.832720000
	C	-4.385366000	-3.074908000	-2.005950000
	H	-5.065359000	-1.193922000	-1.191669000
	C	-2.082857000	-3.664722000	-1.543303000
	H	-0.951787000	-2.230320000	-0.381463000
	C	-3.316269000	-3.965630000	-2.131271000
	H	-5.351588000	-3.309612000	-2.458673000

	H	-1.245527000	-4.360316000	-1.636460000
	H	-3.445224000	-4.898671000	-2.684974000
	C	-2.135694000	1.188603000	-1.003657000
	C	-1.241996000	2.190196000	-0.603125000
	C	-2.525803000	1.088107000	-2.348124000
	C	-0.732761000	3.080297000	-1.548680000
	H	-0.893011000	2.246312000	0.427145000
	C	-2.027352000	1.995331000	-3.285186000
	H	-3.200278000	0.292519000	-2.669504000
	C	-1.126068000	2.988272000	-2.886458000
	H	-0.000071000	3.824133000	-1.232210000
	H	-2.331505000	1.915790000	-4.331490000
	H	-0.720066000	3.683956000	-3.625095000
INT-4A	C	2.168294000	1.623091000	0.911040000
	C	3.193928000	1.618265000	-0.085797000
	C	4.515099000	1.237992000	0.342952000
	C	4.775575000	0.805165000	1.615668000
	C	3.725358000	0.717384000	2.585536000
	C	2.463140000	1.169486000	2.242936000
	H	1.232947000	2.155222000	0.742575000
	H	5.327135000	1.278774000	-0.387566000
	H	5.781038000	0.479177000	1.890844000
	H	1.665853000	1.179424000	2.990679000
	C	4.022849000	0.144707000	3.943330000
	H	3.161275000	0.229894000	4.621457000
	H	4.285764000	-0.923344000	3.856375000
	H	4.885294000	0.649935000	4.409266000
	C	3.079144000	2.418498000	-1.384250000
	H	3.461462000	1.771464000	-2.192086000
	C	3.965824000	3.675575000	-1.321222000
	H	5.022037000	3.433972000	-1.132195000
	H	3.913333000	4.235397000	-2.269136000
	H	3.628432000	4.345544000	-0.512504000
	C	1.651412000	2.822271000	-1.754141000
	H	1.640205000	3.305555000	-2.743185000
	H	0.981668000	1.957878000	-1.792377000
	H	1.242006000	3.547426000	-1.031040000
	Ru	2.018040000	-0.363241000	0.082765000
	C	2.950879000	-2.692892000	0.319664000
	O	3.715209000	-1.685109000	0.388305000
	O	1.701586000	-2.493747000	0.179606000
	C	3.504231000	-4.090133000	0.353322000
	H	4.441042000	-4.119547000	0.925778000
	H	2.767094000	-4.787905000	0.773436000

	H	3.721093000	-4.403964000	-0.680909000
	C	-0.433712000	-0.184275000	-1.388748000
	O	0.041374000	0.197364000	-0.245370000
	C	0.574690000	-0.536244000	-2.393354000
	C	0.326322000	-0.770367000	-3.754602000
	C	1.895249000	-0.589633000	-1.865052000
	C	1.386578000	-1.047853000	-4.615121000
	H	-0.694909000	-0.722554000	-4.142709000
	C	2.948862000	-0.878753000	-2.751174000
	C	2.694946000	-1.099830000	-4.106917000
	H	1.202303000	-1.223099000	-5.677626000
	H	3.969186000	-0.941494000	-2.364757000
	H	3.526224000	-1.321925000	-4.783094000
	C	-1.820107000	-0.263440000	-1.553670000
	H	-2.297250000	-0.592154000	-2.474330000
	P	-2.810692000	-0.025809000	-0.151123000
	C	-4.483813000	-0.607234000	-0.558348000
	C	-5.066980000	-1.688145000	0.115818000
	C	-5.185111000	0.031984000	-1.594682000
	C	-6.343700000	-2.127444000	-0.246384000
	H	-4.522844000	-2.187004000	0.919803000
	C	-6.457589000	-0.412788000	-1.953078000
	H	-4.735155000	0.880340000	-2.115811000
	C	-7.038275000	-1.492568000	-1.278673000
	H	-6.794859000	-2.971142000	0.280755000
	H	-6.999677000	0.085564000	-2.760004000
	H	-8.035815000	-1.838510000	-1.559531000
	C	-3.014517000	1.709159000	0.384976000
	C	-4.222511000	2.160854000	0.938025000
	C	-1.935656000	2.596259000	0.239362000
	C	-4.347921000	3.490504000	1.349482000
	H	-5.069510000	1.478967000	1.039244000
	C	-2.069624000	3.922601000	0.651479000
	H	-0.998666000	2.236289000	-0.184909000
	C	-3.272255000	4.371217000	1.207617000
	H	-5.290849000	3.839338000	1.777137000
	H	-1.227795000	4.609315000	0.536001000
	H	-3.372775000	5.411253000	1.527147000
	C	-2.228470000	-0.947693000	1.306720000
	C	-1.384422000	-2.051276000	1.123559000
	C	-2.596316000	-0.550668000	2.600859000
	C	-0.907644000	-2.751265000	2.231534000
	H	-1.066218000	-2.340454000	0.122098000
	C	-2.130541000	-1.266958000	3.705240000

	H	-3.230191000	0.325323000	2.750093000
	C	-1.283210000	-2.364385000	3.521084000
	H	-0.216057000	-3.580489000	2.075229000
	H	-2.417743000	-0.956897000	4.712721000
	H	-0.903846000	-2.910901000	4.388032000
TS-1B	C	-2.927114000	-0.702535000	1.403212000
	C	-1.617450000	-1.188771000	1.650109000
	C	-1.062812000	-2.148032000	0.735248000
	C	-1.755259000	-2.546881000	-0.421497000
	C	-3.003136000	-1.933312000	-0.754672000
	C	-3.589200000	-1.029270000	0.191953000
	H	-3.353668000	0.083774000	2.021761000
	H	-0.033576000	-2.474516000	0.874769000
	H	-1.238640000	-3.146085000	-1.167971000
	H	-4.495965000	-0.482066000	-0.066288000
	C	-3.657663000	-2.207652000	-2.077238000
	H	-4.275219000	-1.356012000	-2.395681000
	H	-2.900598000	-2.403681000	-2.849296000
	H	-4.310386000	-3.093936000	-1.999390000
	C	-0.814950000	-0.690335000	2.838011000
	H	0.198707000	-1.103335000	2.724270000
	C	-1.416396000	-1.243321000	4.138937000
	H	-1.494007000	-2.341884000	4.115848000
	H	-0.792476000	-0.962086000	5.001699000
	H	-2.426624000	-0.837688000	4.309057000
	C	-0.685472000	0.837521000	2.862971000
	H	-0.090695000	1.151116000	3.735016000
	H	-0.182641000	1.193956000	1.954024000
	H	-1.668288000	1.329027000	2.934596000
	Ru	-1.642447000	-0.331947000	-0.348000000
	C	0.233703000	-0.803259000	-2.625325000
	O	-0.570057000	0.029769000	-2.026888000
	O	0.246365000	-2.023961000	-2.483481000
	C	1.250521000	-0.104865000	-3.511040000
	C	-2.807154000	2.378529000	0.081206000
	O	-3.810157000	1.945632000	0.629545000
	O	-1.881525000	1.650590000	-0.500269000
	C	-2.508420000	3.865396000	0.004776000
	H	1.709495000	-0.819010000	-4.207190000
	H	0.791520000	0.732581000	-4.054308000
	H	2.033853000	0.320102000	-2.861057000
	H	-2.399243000	4.169292000	-1.047521000
	H	-1.548042000	4.073088000	0.500919000
	H	-3.313147000	4.439177000	0.480950000

	C	2.367554000	0.469143000	0.203722000
	O	1.316032000	-0.205025000	0.347127000
	C	2.298046000	1.957365000	0.032978000
	C	3.304266000	2.803867000	0.527165000
	C	1.199006000	2.512562000	-0.640155000
	C	3.213715000	4.185670000	0.353410000
	H	4.150312000	2.379401000	1.071720000
	C	1.123026000	3.894079000	-0.828390000
	H	0.415199000	1.863331000	-1.027244000
	C	2.124255000	4.733648000	-0.331263000
	H	3.993973000	4.837088000	0.754706000
	H	0.271438000	4.313667000	-1.367665000
	H	2.056286000	5.815026000	-0.474802000
	C	3.653991000	-0.119646000	0.180394000
	H	4.602952000	0.370475000	-0.023087000
	S	3.813508000	-1.794628000	0.343435000
	C	2.894919000	-2.652971000	-0.940768000
	H	2.933267000	-3.728534000	-0.721274000
	H	1.868151000	-2.266880000	-0.994205000
	H	3.434417000	-2.427403000	-1.869394000
	C	2.966203000	-2.310513000	1.848879000
	H	1.980916000	-1.828613000	1.836286000
	H	2.910036000	-3.406805000	1.861352000
	H	3.592231000	-1.934832000	2.667889000
	O	5.215208000	-2.256571000	0.354318000
INT-1B	C	2.612937000	-1.660519000	0.701457000
	C	2.720120000	-0.734004000	-0.349300000
	C	1.981880000	-1.018001000	-1.556852000
	C	1.274482000	-2.220706000	-1.738831000
	C	1.173027000	-3.160570000	-0.656929000
	C	1.830963000	-2.853966000	0.560204000
	H	3.046520000	-1.427360000	1.671823000
	H	1.945617000	-0.272249000	-2.352350000
	H	0.678406000	-2.361416000	-2.638916000
	H	1.678857000	-3.498826000	1.426669000
	C	0.315781000	-4.384315000	-0.788126000
	H	-0.073684000	-4.698809000	0.189682000
	H	-0.538773000	-4.194041000	-1.450380000
	H	0.906185000	-5.213200000	-1.213551000
	C	3.556152000	0.529111000	-0.265631000
	H	2.884719000	1.359322000	-0.527271000
	C	4.670136000	0.480381000	-1.326093000
	H	4.273381000	0.338726000	-2.343186000
	H	5.251559000	1.415966000	-1.316765000

	H	5.364951000	-0.349645000	-1.119677000
	C	4.133661000	0.812769000	1.121187000
	H	4.653870000	1.783821000	1.117317000
	H	3.344648000	0.839092000	1.884303000
	H	4.878527000	0.049585000	1.401271000
	Ru	0.526611000	-1.209114000	0.056210000
	C	-1.944882000	-1.642396000	-1.462665000
	O	-1.395499000	-1.836549000	-0.297011000
	O	-1.439044000	-1.036142000	-2.402649000
	C	-3.341678000	-2.235181000	-1.565091000
	C	0.361615000	-0.109376000	2.806882000
	O	1.272292000	0.674520000	2.525952000
	O	-0.141286000	-1.001630000	2.018633000
	C	-0.317345000	-0.046979000	4.168139000
	H	-3.617590000	-2.357852000	-2.620726000
	H	-3.405993000	-3.192498000	-1.028857000
	H	-4.053430000	-1.543024000	-1.091936000
	H	-0.618873000	-1.046819000	4.509676000
	H	-1.232189000	0.561529000	4.071400000
	H	0.343511000	0.434853000	4.900712000
	C	-1.240502000	1.487223000	-0.183884000
	O	-0.174839000	0.788470000	-0.163110000
	C	-2.623899000	0.977236000	0.066869000
	C	-3.680525000	1.434233000	-0.738489000
	C	-2.902105000	0.122171000	1.143525000
	C	-4.996857000	1.063953000	-0.459170000
	H	-3.463035000	2.066731000	-1.600843000
	C	-4.221744000	-0.229603000	1.430366000
	H	-2.081938000	-0.279963000	1.734458000
	C	-5.272074000	0.241694000	0.637291000
	H	-5.807871000	1.415779000	-1.101105000
	H	-4.427587000	-0.890026000	2.275908000
	H	-6.302537000	-0.043327000	0.863610000
	C	-1.149252000	2.866641000	-0.415286000
	H	-1.960484000	3.588730000	-0.374757000
	S	0.371864000	3.591453000	-0.630754000
	O	0.294676000	5.045458000	-0.871033000
	C	1.186269000	2.783288000	-2.018659000
	H	2.230489000	3.119218000	-2.057077000
	H	1.086005000	1.701537000	-1.879130000
	H	0.624766000	3.130027000	-2.895545000
	C	1.406531000	3.256604000	0.799799000
	H	1.325084000	2.199817000	1.103506000
	H	2.433657000	3.558661000	0.556245000

	H	0.985990000	3.912294000	1.573799000
TS-2B	Ru	-0.546461000	-0.773800000	-0.147470000
	C	0.802353000	-0.008608000	-2.633965000
	O	1.477407000	-1.034367000	-2.534766000
	O	-0.097252000	0.374617000	-1.780754000
	C	0.975656000	0.957327000	-3.792052000
	H	0.008773000	1.121383000	-4.291331000
	H	1.301474000	1.933890000	-3.402232000
	H	1.712179000	0.571241000	-4.507687000
	C	1.860562000	1.015803000	0.373821000
	O	1.280151000	-0.114839000	0.615965000
	C	1.061486000	2.216212000	0.039142000
	C	1.559588000	3.219810000	-0.807092000
	C	-0.246839000	2.309368000	0.535657000
	C	0.743727000	4.290421000	-1.171635000
	H	2.568959000	3.140043000	-1.216098000
	C	-1.060336000	3.380810000	0.164945000
	C	-0.569726000	4.369299000	-0.692115000
	H	1.127118000	5.060177000	-1.845653000
	H	-2.081156000	3.431811000	0.550295000
	H	-1.208224000	5.204921000	-0.989266000
	C	3.247105000	1.095882000	0.438866000
	H	3.854507000	1.995880000	0.383178000
	C	-1.398084000	-2.266894000	-1.433207000
	C	-0.345281000	-2.819198000	-0.642643000
	C	-0.399780000	-2.763886000	0.793133000
	C	-1.483789000	-2.088664000	1.392979000
	C	-2.472250000	-1.451612000	0.589376000
	C	-2.476831000	-1.565879000	-0.833082000
	H	-1.281190000	-2.252344000	-2.518187000
	H	0.528425000	-3.233785000	-1.146526000
	H	-1.478677000	-1.874686000	2.460444000
	H	-3.157141000	-0.742416000	1.087959000
	C	0.725495000	-3.321435000	1.613480000
	H	0.814024000	-2.790292000	2.571020000
	H	0.540364000	-4.386866000	1.829928000
	H	1.680257000	-3.252809000	1.073214000
	C	-3.517778000	-0.839625000	-1.664889000
	C	-3.528748000	0.672550000	-1.391643000
	C	-4.898634000	-1.467930000	-1.424204000
	H	-3.244361000	-0.997816000	-2.722206000
	H	-2.554593000	1.118265000	-1.639847000
	H	-4.298766000	1.155435000	-2.014169000
	H	-3.751896000	0.888442000	-0.334694000

	H	-4.894435000	-2.551721000	-1.622644000
	H	-5.218883000	-1.309530000	-0.382279000
	H	-5.650395000	-1.002750000	-2.080544000
	H	-0.620882000	1.567479000	1.255376000
	C	-2.633010000	1.078367000	2.677901000
	O	-3.661413000	0.847217000	2.003528000
	O	-1.502804000	0.514836000	2.536601000
	C	-2.704017000	2.175549000	3.748246000
	H	-2.169891000	1.860150000	4.657204000
	H	-2.187785000	3.073602000	3.367503000
	H	-3.745045000	2.437828000	3.981817000
	S	4.169739000	-0.317256000	0.694234000
	C	3.768982000	-1.521465000	-0.575310000
	H	4.155853000	-2.495690000	-0.247947000
	H	2.688485000	-1.514910000	-0.778489000
	H	4.306941000	-1.163042000	-1.462182000
	C	3.649277000	-1.085582000	2.236740000
	H	4.164436000	-2.051415000	2.323546000
	H	3.981692000	-0.390009000	3.018069000
	H	2.557236000	-1.178296000	2.216341000
	O	5.619697000	-0.054038000	0.728470000
INT-2B	Ru	-0.798228000	-0.399202000	-0.645885000
	C	-1.843590000	2.226671000	-1.480867000
	O	-2.914001000	1.942447000	-0.955230000
	O	-0.788496000	1.458650000	-1.512961000
	C	-1.615023000	3.573678000	-2.148985000
	H	-0.904863000	4.156111000	-1.542121000
	H	-1.163937000	3.441211000	-3.143538000
	H	-2.562031000	4.122327000	-2.227590000
	C	1.677324000	0.970185000	0.242809000
	O	1.248669000	-0.050097000	-0.408111000
	C	0.668653000	1.803804000	0.944813000
	C	0.713728000	3.201528000	0.912309000
	C	-0.424176000	1.144281000	1.545550000
	C	-0.344995000	3.941696000	1.442121000
	H	1.550080000	3.705178000	0.423902000
	C	-1.480557000	1.899863000	2.066999000
	C	-1.448582000	3.293126000	2.006808000
	H	-0.320748000	5.032970000	1.393044000
	H	-2.326714000	1.392925000	2.530525000
	H	-2.283324000	3.876862000	2.400158000
	C	3.026043000	1.291806000	0.300690000
	H	3.463174000	2.086387000	0.901205000
	C	-2.695190000	-1.036806000	-1.510215000

	C	-1.588716000	-1.425882000	-2.324202000
	C	-0.508504000	-2.195730000	-1.783284000
	C	-0.569024000	-2.589259000	-0.409302000
	C	-1.636590000	-2.155480000	0.394862000
	C	-2.716149000	-1.365499000	-0.131812000
	H	-3.436240000	-0.344398000	-1.906547000
	H	-1.516195000	-1.045975000	-3.345366000
	H	0.316480000	-2.998188000	0.100122000
	H	-1.503494000	-2.297428000	1.468018000
	C	0.706473000	-2.509851000	-2.605255000
	H	1.590252000	-2.584369000	-1.957377000
	H	0.577102000	-3.479749000	-3.114216000
	H	0.879006000	-1.741260000	-3.372679000
	C	-3.812407000	-0.798945000	0.755105000
	C	-3.598707000	-1.083351000	2.244261000
	C	-5.183108000	-1.326220000	0.297655000
	H	-3.791415000	0.289561000	0.581506000
	H	-2.604366000	-0.782157000	2.601923000
	H	-4.353893000	-0.545620000	2.837705000
	H	-3.708182000	-2.157234000	2.465651000
	H	-5.392091000	-1.071625000	-0.751978000
	H	-5.239555000	-2.422438000	0.401376000
	H	-5.983306000	-0.887176000	0.913253000
	H	-0.361594000	0.059410000	1.748504000
	C	1.376259000	-1.914353000	2.336660000
	O	0.165644000	-1.720053000	2.604346000
	O	1.834043000	-2.561309000	1.351352000
	C	2.403023000	-1.243989000	3.266233000
	H	3.331898000	-1.830713000	3.328121000
	H	2.654718000	-0.249127000	2.859434000
	H	1.978943000	-1.096221000	4.268873000
	S	4.209401000	0.321629000	-0.466436000
	O	5.535161000	0.969788000	-0.467911000
	C	4.297536000	-1.291945000	0.299260000
	H	4.823400000	-1.098509000	1.243997000
	H	3.290115000	-1.728145000	0.519215000
	H	4.913750000	-1.924412000	-0.355335000
	C	3.671426000	-0.011359000	-2.149839000
	H	3.667237000	0.966390000	-2.647979000
	H	4.420170000	-0.679876000	-2.594364000
	H	2.668182000	-0.449126000	-2.111666000
	Ru	-0.673937000	-0.168582000	-0.680042000
TS-3B	C	-1.587739000	2.639510000	-1.036852000
	O	-2.732291000	2.311497000	-0.738727000

O	-0.573620000	1.836629000	-1.177685000
C	-1.214157000	4.101669000	-1.237318000
H	-0.709088000	4.454011000	-0.323423000
H	-0.509350000	4.221466000	-2.072470000
H	-2.116407000	4.705188000	-1.400651000
C	1.817226000	0.879204000	0.446152000
O	1.390114000	0.085195000	-0.468317000
C	0.791184000	1.502458000	1.302439000
C	0.954546000	2.741456000	1.924132000
C	-0.440133000	0.799932000	1.368572000
C	-0.121864000	3.315633000	2.605674000
H	1.900239000	3.280848000	1.838690000
C	-1.502342000	1.403124000	2.061362000
C	-1.351250000	2.650491000	2.667580000
H	-0.005452000	4.293734000	3.078844000
H	-2.457742000	0.886311000	2.133412000
H	-2.194271000	3.109595000	3.188904000
C	3.178729000	1.123240000	0.610765000
H	3.640683000	1.714113000	1.397523000
C	-2.462470000	-0.460826000	-1.893890000
C	-1.307295000	-0.785809000	-2.672761000
C	-0.354775000	-1.723011000	-2.193624000
C	-0.599417000	-2.365612000	-0.935845000
C	-1.741692000	-2.033846000	-0.181133000
C	-2.706956000	-1.076915000	-0.642944000
H	-3.104530000	0.360287000	-2.208990000
H	-1.100825000	-0.225342000	-3.585486000
H	0.171053000	-2.992393000	-0.486808000
H	-1.819398000	-2.434461000	0.825893000
C	0.927102000	-1.985487000	-2.928066000
H	1.757127000	-2.081637000	-2.214430000
H	0.856042000	-2.928438000	-3.495209000
H	1.156118000	-1.175345000	-3.634775000
C	-3.918406000	-0.670815000	0.178325000
C	-3.939438000	-1.291285000	1.578965000
C	-5.211743000	-1.015243000	-0.578504000
H	-3.856131000	0.426302000	0.260670000
H	-3.002519000	-1.136488000	2.132882000
H	-4.760005000	-0.854982000	2.168684000
H	-4.114189000	-2.379012000	1.529617000
H	-5.252035000	-0.525744000	-1.562869000
H	-5.300651000	-2.103397000	-0.734597000
H	-6.090646000	-0.683270000	-0.004112000
H	-0.358767000	-0.434671000	1.534366000

	C	0.698734000	-2.427359000	2.243413000
	O	-0.286245000	-1.623753000	2.386490000
	O	1.562411000	-2.378215000	1.351253000
	C	0.741902000	-3.571512000	3.258189000
	H	0.012256000	-4.342751000	2.958723000
	H	1.740390000	-4.028704000	3.284211000
	H	0.450120000	-3.215925000	4.256725000
	S	4.300207000	0.252605000	-0.326911000
	O	5.697613000	0.655309000	-0.079700000
	C	4.083804000	-1.507249000	-0.033371000
	H	4.650534000	-1.683017000	0.890659000
	H	3.018149000	-1.735640000	0.144513000
	H	4.529006000	-2.057044000	-0.873589000
	C	3.893570000	0.470122000	-2.068019000
	H	4.086404000	1.531456000	-2.269518000
	H	4.571595000	-0.172366000	-2.645012000
	H	2.835628000	0.218739000	-2.205913000
INT-3B	Ru	0.612560000	-0.518428000	-0.261631000
	C	0.530884000	0.587991000	2.541518000
	O	1.752398000	0.677826000	2.572905000
	O	-0.175703000	0.086870000	1.572992000
	C	-0.329655000	1.093885000	3.696012000
	H	-0.938030000	1.944223000	3.348803000
	H	-1.025273000	0.308913000	4.031933000
	H	0.304345000	1.414051000	4.532478000
	C	-1.916129000	0.784784000	-0.737390000
	O	-1.435988000	-0.409572000	-0.669803000
	C	-0.946660000	1.859824000	-0.964575000
	C	-1.288263000	3.184121000	-1.282324000
	C	0.399660000	1.454544000	-0.772072000
	C	-0.285267000	4.138113000	-1.427797000
	H	-2.335283000	3.464964000	-1.423996000
	C	1.385396000	2.445980000	-0.901674000
	C	1.051294000	3.762618000	-1.230279000
	H	-0.536885000	5.168950000	-1.687254000
	H	2.435403000	2.201344000	-0.733268000
	H	1.842992000	4.510858000	-1.329521000
	C	-3.283467000	0.987060000	-0.519103000
	H	-3.806894000	1.939819000	-0.495225000
	C	2.184972000	-1.749904000	0.674562000
	C	1.222656000	-2.683449000	0.188779000
	C	0.763294000	-2.579883000	-1.144126000
	C	1.364452000	-1.608106000	-2.009797000
	C	2.361218000	-0.729179000	-1.512316000

	C	2.828574000	-0.791915000	-0.158958000
	H	2.412831000	-1.745422000	1.737922000
	H	0.751757000	-3.385553000	0.877617000
	H	1.007293000	-1.497882000	-3.034786000
	H	2.746378000	0.040673000	-2.179515000
	C	-0.368503000	-3.428742000	-1.649682000
	H	-1.037829000	-2.834708000	-2.288977000
	H	0.014852000	-4.269884000	-2.251093000
	H	-0.956122000	-3.841916000	-0.817552000
	C	3.909330000	0.148181000	0.371854000
	C	4.929726000	0.515165000	-0.713936000
	C	4.612767000	-0.415953000	1.610923000
	H	3.395735000	1.063761000	0.702468000
	H	4.478850000	1.069477000	-1.550734000
	H	5.716360000	1.157738000	-0.289579000
	H	5.415957000	-0.383863000	-1.128520000
	H	3.916064000	-0.477374000	2.456470000
	H	5.044970000	-1.412014000	1.414814000
	H	5.432728000	0.253960000	1.912946000
	S	-4.153616000	-0.256188000	0.241420000
	O	-5.556878000	0.101725000	0.523973000
	C	-4.095413000	-1.760953000	-0.748034000
	H	-4.608782000	-1.507601000	-1.684398000
	H	-3.042402000	-2.012716000	-0.918738000
	H	-4.645566000	-2.532346000	-0.192559000
	C	-3.305260000	-0.721544000	1.766639000
	H	-3.613120000	0.058261000	2.475733000
	H	-3.675600000	-1.705122000	2.085365000
	H	-2.213805000	-0.691360000	1.608338000
TS-4B	C	-1.282449000	-0.300492000	-2.001342000
	C	-2.123944000	0.649197000	-1.346620000
	C	-3.275340000	0.133216000	-0.647428000
	C	-3.451629000	-1.215678000	-0.463555000
	C	-2.522282000	-2.168386000	-1.017733000
	C	-1.524248000	-1.709383000	-1.869249000
	H	-0.507251000	0.037503000	-2.689068000
	H	-3.974312000	0.839621000	-0.192961000
	H	-4.260345000	-1.581447000	0.170484000
	H	-0.867653000	-2.418514000	-2.378284000
	C	-2.692632000	-3.622216000	-0.682308000
	H	-1.922012000	-4.245702000	-1.157460000
	H	-2.629974000	-3.756041000	0.409446000
	H	-3.683648000	-3.988063000	-0.999198000
	C	-2.056586000	2.141920000	-1.636606000

	H	-2.225928000	2.652409000	-0.673036000
	C	-3.186356000	2.549388000	-2.597244000
	H	-4.177403000	2.269511000	-2.208301000
	H	-3.184406000	3.638441000	-2.764994000
	H	-3.060643000	2.054815000	-3.574820000
	C	-0.702398000	2.610642000	-2.171031000
	H	-0.681497000	3.709167000	-2.237462000
	H	0.118490000	2.293935000	-1.515457000
	H	-0.511445000	2.219434000	-3.183920000
	Ru	-0.713343000	-0.378686000	0.076571000
	C	-0.896250000	-1.620795000	2.457266000
	O	-2.046324000	-1.314684000	2.099848000
	O	0.121818000	-1.386833000	1.704075000
	C	-0.621895000	-2.243360000	3.808054000
	H	-1.510724000	-2.775912000	4.171779000
	H	0.244777000	-2.917667000	3.762574000
	H	-0.384894000	-1.437093000	4.521433000
	C	1.902716000	0.761784000	0.066352000
	O	1.218832000	-0.145725000	-0.554953000
	C	1.130722000	1.684644000	0.889059000
	C	1.631138000	2.832829000	1.525079000
	C	-0.230699000	1.293862000	1.014090000
	C	0.777590000	3.618763000	2.292872000
	H	2.682099000	3.113122000	1.413263000
	C	-1.065338000	2.091945000	1.817599000
	C	-0.568499000	3.239687000	2.437770000
	H	1.150990000	4.519242000	2.785506000
	H	-2.107935000	1.799741000	1.965085000
	H	-1.234775000	3.851230000	3.053448000
	C	3.299118000	0.761421000	-0.056915000
	H	3.999673000	1.428148000	0.439398000
	S	4.041763000	-0.594034000	-0.746654000
	O	5.515247000	-0.543593000	-0.707946000
	C	3.441713000	-2.089608000	0.072423000
	H	2.349432000	-2.024155000	0.171059000
	H	3.770459000	-2.957066000	-0.515300000
	H	3.930793000	-2.071123000	1.054779000
	C	3.477046000	-0.809463000	-2.445771000
	H	2.380345000	-0.821287000	-2.433365000
	H	3.867450000	0.062545000	-2.985892000
	H	3.915660000	-1.740893000	-2.828148000
	C	1.057598000	-1.272633000	1.421203000
INT-4B	C	1.809885000	-0.073340000	1.614132000
	C	3.195610000	-0.103327000	1.222074000

	C	3.755598000	-1.186008000	0.599437000
	C	2.972447000	-2.351373000	0.316909000
	C	1.666562000	-2.401433000	0.772688000
	H	0.111313000	-1.416424000	1.942625000
	H	3.805394000	0.783744000	1.409357000
	H	4.797642000	-1.155130000	0.274146000
	H	1.080095000	-3.314243000	0.638228000
	C	3.592277000	-3.487209000	-0.448266000
	H	2.919798000	-4.355025000	-0.511392000
	H	3.835965000	-3.167299000	-1.475510000
	H	4.537129000	-3.811389000	0.018535000
	C	1.377810000	1.015258000	2.597870000
	H	1.523640000	1.982700000	2.088302000
	C	2.272537000	0.997883000	3.850538000
	H	3.336027000	1.139842000	3.609405000
	H	1.977181000	1.798949000	4.547271000
	H	2.176289000	0.035308000	4.380082000
	C	-0.088861000	0.930292000	3.023165000
	H	-0.357921000	1.810049000	3.627477000
	H	-0.763082000	0.895775000	2.161591000
	H	-0.271717000	0.037584000	3.644167000
	Ru	0.708840000	-0.199667000	-0.427565000
	C	1.696023000	0.233386000	-2.692586000
	O	2.433867000	0.223253000	-1.660319000
	O	0.459819000	-0.029256000	-2.543899000
	C	2.250561000	0.589891000	-4.041681000
	H	3.305347000	0.292258000	-4.114119000
	H	1.655710000	0.122914000	-4.837963000
	H	2.192399000	1.683837000	-4.164724000
	C	-2.057352000	0.510632000	-0.051627000
	O	-1.308670000	-0.539262000	0.028563000
	C	-1.358686000	1.786745000	-0.177318000
	C	-1.963566000	3.054238000	-0.138827000
	C	0.051292000	1.643147000	-0.313540000
	C	-1.177724000	4.198934000	-0.228051000
	H	-3.047611000	3.144763000	-0.029685000
	C	0.823545000	2.816961000	-0.410622000
	C	0.215731000	4.072260000	-0.364511000
	H	-1.639220000	5.188235000	-0.193089000
	H	1.906537000	2.734690000	-0.531496000
	H	0.831996000	4.973315000	-0.439515000
	C	-3.454755000	0.374658000	-0.014285000
	H	-4.194930000	1.164772000	-0.108253000
	S	-4.131146000	-1.172133000	0.014499000

	O	-5.605710000	-1.175648000	-0.006755000
	C	-3.480880000	-2.148538000	-1.358629000
	H	-2.387372000	-2.050180000	-1.353196000
	H	-3.820659000	-3.185025000	-1.231833000
	H	-3.921184000	-1.694100000	-2.255350000
	C	-3.526627000	-2.076664000	1.453965000
	H	-2.431687000	-2.007228000	1.452135000
	H	-3.964625000	-1.558427000	2.316444000
	H	-3.891689000	-3.110053000	1.384306000
CAT-Sol	C	-1.313433000	1.772649000	-0.286879000
	C	-2.441758000	1.002051000	-0.671626000
	C	-2.299055000	0.093284000	-1.774450000
	C	-1.042765000	-0.088530000	-2.390767000
	C	0.108246000	0.679621000	-1.994534000
	C	-0.058638000	1.619259000	-0.952042000
	H	-1.350544000	2.404781000	0.596742000
	H	-3.132367000	-0.558265000	-2.042909000
	H	-0.917615000	-0.886397000	-3.125826000
	H	0.809063000	2.162485000	-0.569055000
	C	1.449929000	0.416384000	-2.616417000
	H	2.214375000	1.067819000	-2.172797000
	H	1.759846000	-0.629232000	-2.456108000
	H	1.411635000	0.613493000	-3.699999000
	C	-3.754862000	1.073547000	0.082207000
	H	-4.274732000	0.120268000	-0.110019000
	C	-4.610754000	2.212731000	-0.494358000
	H	-4.766758000	2.094994000	-1.578155000
	H	-5.598314000	2.237501000	-0.007949000
	H	-4.124927000	3.187677000	-0.327428000
	C	-3.573057000	1.203215000	1.597653000
	H	-4.551357000	1.139781000	2.098103000
	H	-2.930347000	0.400591000	1.987025000
	H	-3.124441000	2.169624000	1.875461000
	Ru	-0.804568000	-0.342516000	-0.245652000
	C	-1.087706000	-2.755652000	0.487939000
	O	-0.186922000	-2.425652000	-0.350058000
	O	-1.925049000	-1.864406000	0.807773000
	C	-1.116280000	-4.124918000	1.093961000
	C	0.770147000	0.461696000	2.201310000
	O	0.575727000	1.665977000	2.018834000
	O	0.295188000	-0.496104000	1.473349000
	C	1.663396000	-0.024078000	3.329974000
	H	-2.106804000	-4.343796000	1.512277000
	H	-0.831940000	-4.879781000	0.347857000

	H	-0.372573000	-4.154434000	1.906690000
	H	2.649395000	-0.279093000	2.907653000
	H	1.255900000	-0.935088000	3.789555000
	H	1.793824000	0.765855000	4.080539000
	C	4.438111000	2.538126000	-0.523228000
	H	5.289102000	1.840824000	-0.563464000
	H	4.819121000	3.549732000	-0.313854000
	H	3.960321000	2.559487000	-1.516088000
	C	3.440805000	2.121641000	0.542040000
	H	3.952802000	2.057447000	1.524521000
	H	3.062563000	1.102501000	0.321324000
	O	2.387874000	3.058895000	0.580232000
	H	1.743380000	2.743758000	1.246882000
	C	3.100348000	-1.897603000	-0.152358000
	H	3.248000000	-2.678511000	0.623108000
	H	2.526956000	-1.085881000	0.333554000
	C	4.451325000	-1.385270000	-0.616454000
	H	5.021645000	-2.191664000	-1.103626000
	H	4.330785000	-0.568143000	-1.344783000
	H	5.040329000	-1.003552000	0.232696000
	O	2.397473000	-2.417409000	-1.261882000
	H	1.496354000	-2.633393000	-0.961040000
TS-1ASol	C	-3.065685000	-0.642874000	-1.708564000
	C	-1.650212000	-0.603427000	-1.672974000
	C	-1.000869000	0.676825000	-1.633262000
	C	-1.738007000	1.877023000	-1.596156000
	C	-3.162572000	1.831734000	-1.486141000
	C	-3.808623000	0.554393000	-1.556704000
	H	-3.586939000	-1.600471000	-1.688433000
	H	0.079994000	0.705442000	-1.525609000
	H	-1.227477000	2.833458000	-1.488670000
	H	-4.884007000	0.483569000	-1.391810000
	C	-3.951949000	3.087841000	-1.263791000
	H	-4.920643000	2.863423000	-0.795410000
	H	-3.389582000	3.770252000	-0.614472000
	H	-4.142628000	3.585789000	-2.229857000
	C	-0.837672000	-1.881445000	-1.651132000
	H	0.190646000	-1.592301000	-1.398154000
	C	-0.845317000	-2.515781000	-3.050058000
	H	-0.511061000	-1.801487000	-3.819387000
	H	-0.166800000	-3.382755000	-3.075735000
	H	-1.855925000	-2.861443000	-3.321661000
	C	-1.325292000	-2.858932000	-0.575724000
	H	-0.777659000	-3.810339000	-0.652145000

	H	-1.149597000	-2.435106000	0.421945000
	H	-2.395041000	-3.080862000	-0.691215000
	Ru	-2.377517000	0.535756000	0.042415000
	C	-1.290381000	2.811107000	1.678573000
	O	-1.802532000	1.623379000	1.635479000
	O	-1.525328000	3.707843000	0.863986000
	C	-0.344389000	3.022493000	2.843037000
	C	-4.186066000	-1.421738000	1.412660000
	O	-5.007821000	-1.446318000	0.495100000
	O	-3.215391000	-0.569178000	1.518445000
	C	-4.223789000	-2.435243000	2.541998000
	H	-0.098120000	4.085728000	2.957198000
	H	-0.777744000	2.615064000	3.767177000
	H	0.573467000	2.443930000	2.655129000
	H	-4.131890000	-1.927755000	3.513055000
	H	-3.354440000	-3.105443000	2.444698000
	H	-5.148361000	-3.024855000	2.503419000
	C	1.318361000	-0.554402000	1.865152000
	O	0.537449000	-0.653578000	0.883503000
	C	0.749202000	-0.384050000	3.247672000
	C	1.525365000	0.018387000	4.348232000
	C	-0.625341000	-0.588117000	3.430074000
	C	0.938887000	0.212483000	5.599441000
	H	2.592556000	0.209684000	4.225930000
	C	-1.212873000	-0.394611000	4.679895000
	H	-1.240125000	-0.858367000	2.576258000
	C	-0.434682000	0.005912000	5.769300000
	H	1.553492000	0.534720000	6.443769000
	H	-2.289530000	-0.540308000	4.793501000
	H	-0.895243000	0.164749000	6.747809000
	C	2.723974000	-0.576969000	1.697155000
	H	3.446150000	-0.552211000	2.509332000
	P	3.346398000	-0.560806000	0.090130000
	C	5.141988000	-0.279411000	0.195049000
	C	5.737797000	0.879684000	-0.318774000
	C	5.934620000	-1.251033000	0.828787000
	C	7.117629000	1.067484000	-0.194439000
	H	5.124916000	1.634869000	-0.813693000
	C	7.310119000	-1.056264000	0.953275000
	H	5.472862000	-2.160751000	1.219836000
	C	7.903019000	0.103295000	0.441340000
	H	7.578463000	1.972719000	-0.596235000
	H	7.923016000	-1.812871000	1.448507000
	H	8.980993000	0.252960000	0.537215000

	C	2.661426000	0.784963000	-0.927251000
	C	2.583028000	0.712994000	-2.325039000
	C	2.237193000	1.945881000	-0.261596000
	C	2.084055000	1.800303000	-3.049651000
	H	2.887277000	-0.193945000	-2.849264000
	C	1.732509000	3.022720000	-0.988156000
	H	2.286021000	1.982403000	0.828351000
	C	1.653428000	2.950469000	-2.383016000
	H	2.013282000	1.736670000	-4.137987000
	H	1.367636000	3.918247000	-0.485817000
	H	1.225040000	3.792376000	-2.929145000
	C	3.186466000	-2.113265000	-0.858463000
	C	2.224856000	-3.057657000	-0.473222000
	C	4.041331000	-2.383958000	-1.939292000
	C	2.115288000	-4.259842000	-1.175780000
	H	1.564294000	-2.838652000	0.365640000
	C	3.919633000	-3.583617000	-2.643140000
	H	4.814815000	-1.665780000	-2.221028000
	C	2.955281000	-4.521816000	-2.261922000
	H	1.367688000	-4.996031000	-0.872870000
	H	4.585953000	-3.791201000	-3.483604000
	H	2.864261000	-5.463489000	-2.808471000
	C	-1.369966000	6.133206000	-1.604875000
	C	-0.937510000	6.629623000	-2.973093000
	H	-1.292956000	6.959581000	-0.868767000
	H	-2.438847000	5.839327000	-1.636326000
	H	-1.541328000	7.494193000	-3.291105000
	H	0.121392000	6.933303000	-2.953023000
	H	-1.048304000	5.832731000	-3.726225000
	O	-0.554929000	5.046125000	-1.232488000
	H	-0.871836000	4.678362000	-0.378790000
	C	-4.698297000	-4.622649000	-0.856000000
	C	-4.083578000	-5.530850000	-1.906277000
	H	-5.671055000	-5.042204000	-0.525113000
	H	-4.045534000	-4.599896000	0.041748000
	H	-3.974977000	-6.559537000	-1.527410000
	H	-4.718019000	-5.554297000	-2.806256000
	H	-3.089048000	-5.161229000	-2.203369000
	O	-4.863628000	-3.335624000	-1.402615000
	H	-5.121565000	-2.717547000	-0.685841000
INT-1ASol	C	-2.931905000	-0.821866000	-1.633309000
	C	-1.565717000	-1.037927000	-1.933947000
	C	-0.762065000	0.121103000	-2.176484000
	C	-1.287508000	1.429571000	-2.163721000

	C	-2.662425000	1.628572000	-1.826626000
	C	-3.480348000	0.495865000	-1.573806000
	H	-3.573496000	-1.653415000	-1.341447000
	H	0.307550000	-0.013498000	-2.303043000
	H	-0.638480000	2.293278000	-2.294367000
	H	-4.499926000	0.622150000	-1.215074000
	C	-3.201690000	3.019838000	-1.664655000
	H	-3.891967000	3.073026000	-0.811958000
	H	-2.380677000	3.731408000	-1.511991000
	H	-3.748527000	3.316024000	-2.575248000
	C	-0.921540000	-2.407827000	-1.968944000
	H	0.064172000	-2.297505000	-1.489402000
	C	-0.681884000	-2.838140000	-3.424615000
	H	-0.075651000	-2.095265000	-3.966657000
	H	-0.150608000	-3.802790000	-3.461263000
	H	-1.637225000	-2.953544000	-3.961939000
	C	-1.709483000	-3.460799000	-1.192291000
	H	-1.160176000	-4.413514000	-1.180602000
	H	-1.873852000	-3.140475000	-0.152770000
	H	-2.694874000	-3.644721000	-1.645554000
	Ru	-1.752968000	0.402440000	-0.254698000
	C	-1.139562000	3.051011000	0.987545000
	O	-1.938637000	2.050896000	1.003999000
	O	-0.104691000	3.136084000	0.302758000
	C	-1.557875000	4.188938000	1.901639000
	C	-3.747235000	-0.889712000	1.631144000
	O	-4.700795000	-0.693885000	0.867942000
	O	-2.511094000	-0.627703000	1.389648000
	C	-3.980223000	-1.478434000	3.017519000
	H	-0.774728000	4.955805000	1.945091000
	H	-2.492929000	4.632051000	1.523144000
	H	-1.761577000	3.798046000	2.907876000
	H	-3.877582000	-0.673244000	3.763253000
	H	-3.221872000	-2.239865000	3.250219000
	H	-4.989320000	-1.903382000	3.092047000
	C	0.867351000	0.417447000	1.643741000
	O	0.110448000	-0.050238000	0.724966000
	C	0.363986000	1.183357000	2.825958000
	C	1.101402000	2.263387000	3.338449000
	C	-0.813452000	0.788209000	3.475341000
	C	0.677487000	2.927027000	4.490119000
	H	1.993517000	2.601602000	2.808907000
	C	-1.227402000	1.444757000	4.635302000
	H	-1.403949000	-0.015972000	3.039334000

	C	-0.484040000	2.511687000	5.148922000
	H	1.250963000	3.775645000	4.870826000
	H	-2.144029000	1.125295000	5.137602000
	H	-0.815532000	3.027661000	6.053604000
	C	2.239129000	0.145683000	1.617406000
	H	2.905816000	0.428010000	2.429346000
	P	2.942894000	-0.733061000	0.299509000
	C	4.743440000	-0.724604000	0.564264000
	C	5.608645000	-0.033395000	-0.293712000
	C	5.258948000	-1.400577000	1.683089000
	C	6.982191000	-0.019924000	-0.033699000
	H	5.211365000	0.495019000	-1.161802000
	C	6.629844000	-1.380432000	1.938680000
	H	4.586757000	-1.945494000	2.350222000
	C	7.492846000	-0.691071000	1.079520000
	H	7.653927000	0.519736000	-0.704946000
	H	7.027140000	-1.906046000	2.809938000
	H	8.566688000	-0.678586000	1.280012000
	C	2.651949000	0.021110000	-1.325001000
	C	2.823404000	-0.717464000	-2.506775000
	C	2.313471000	1.381306000	-1.383913000
	C	2.674281000	-0.087676000	-3.743595000
	H	3.053353000	-1.783154000	-2.465353000
	C	2.165832000	2.006048000	-2.625457000
	H	2.116725000	1.939052000	-0.466617000
	C	2.351908000	1.273081000	-3.801399000
	H	2.799490000	-0.663594000	-4.663266000
	H	1.854709000	3.052845000	-2.659006000
	H	2.224188000	1.761358000	-4.770357000
	C	2.492223000	-2.498819000	0.220784000
	C	1.284886000	-2.934691000	0.789735000
	C	3.366372000	-3.428161000	-0.367188000
	C	0.964159000	-4.293506000	0.769881000
	H	0.596279000	-2.209870000	1.224392000
	C	3.028355000	-4.782424000	-0.398215000
	H	4.321796000	-3.098614000	-0.781479000
	C	1.828265000	-5.215831000	0.173391000
	H	0.026674000	-4.629120000	1.216733000
	H	3.710383000	-5.502007000	-0.856808000
	H	1.569173000	-6.277106000	0.158431000
	C	-0.157794000	5.755709000	-1.856725000
	C	-0.039347000	6.434939000	-3.209426000
	H	0.458349000	6.297509000	-1.110251000
	H	-1.208069000	5.820988000	-1.501620000

	H	-0.363077000	7.486439000	-3.154913000
	H	1.003768000	6.409542000	-3.563335000
	H	-0.662564000	5.916474000	-3.955429000
	O	0.253332000	4.414211000	-1.972416000
	H	0.185902000	3.984903000	-1.084093000
	C	-5.083025000	-3.906601000	0.297455000
	C	-5.164619000	-5.233469000	-0.436750000
	H	-5.835299000	-3.890437000	1.113110000
	H	-4.091242000	-3.814750000	0.789074000
	H	-5.019239000	-6.079107000	0.254368000
	H	-6.148069000	-5.340306000	-0.920781000
	H	-4.394975000	-5.291748000	-1.222780000
	O	-5.304310000	-2.858170000	-0.614600000
	H	-5.273418000	-2.019484000	-0.105427000
TS-2ASol	Ru	-1.476739000	0.522131000	-0.138862000
	C	-0.766733000	2.840908000	1.556539000
	O	0.129708000	3.116022000	0.743727000
	O	-1.538990000	1.820625000	1.454207000
	C	-0.993390000	3.698074000	2.784493000
	H	-2.027119000	3.615672000	3.144041000
	H	-0.328394000	3.330597000	3.581678000
	H	-0.733091000	4.743464000	2.568453000
	C	0.996098000	-0.176633000	1.526159000
	O	0.400595000	-0.092096000	0.378715000
	C	0.230763000	-0.058336000	2.794950000
	C	0.754612000	0.636525000	3.895604000
	C	-1.039531000	-0.647802000	2.888161000
	C	0.005174000	0.756367000	5.067646000
	H	1.734384000	1.113032000	3.817741000
	C	-1.781639000	-0.532038000	4.061771000
	C	-1.264322000	0.173810000	5.152196000
	H	0.409291000	1.314384000	5.915987000
	H	-2.768335000	-0.997526000	4.110657000
	H	-1.849051000	0.272372000	6.070262000
	C	2.356329000	-0.433236000	1.553954000
	H	2.895718000	-0.596462000	2.484982000
	C	-2.589026000	1.934153000	-1.331390000
	C	-1.364725000	1.562264000	-1.968722000
	C	-1.084530000	0.187645000	-2.286071000
	C	-2.004086000	-0.789068000	-1.848920000
	C	-3.162119000	-0.410891000	-1.107421000
	C	-3.512830000	0.955208000	-0.886781000
	H	-2.734981000	2.977778000	-1.046625000
	H	-0.616479000	2.327480000	-2.178677000

	H	-1.743912000	-1.843865000	-1.912387000
	H	-3.759569000	-1.186155000	-0.628934000
	C	0.203529000	-0.191199000	-2.948927000
	H	0.567573000	-1.160093000	-2.583654000
	H	0.047972000	-0.267645000	-4.038228000
	H	0.975740000	0.564074000	-2.768889000
	C	-4.746481000	1.318278000	-0.081079000
	C	-4.689272000	0.754782000	1.348168000
	C	-6.008510000	0.848578000	-0.822885000
	H	-4.764525000	2.419800000	-0.016236000
	H	-3.853831000	1.195641000	1.909291000
	H	-5.628967000	0.982924000	1.875115000
	H	-4.552170000	-0.336442000	1.346665000
	H	-6.030219000	1.219012000	-1.860842000
	H	-6.060571000	-0.251621000	-0.833236000
	H	-6.907411000	1.225088000	-0.309900000
	H	-1.430609000	-1.218197000	2.042434000
	C	-2.450778000	-3.138856000	0.799631000
	O	-3.500467000	-2.561352000	1.191275000
	O	-1.398225000	-2.553817000	0.434255000
	C	-2.459632000	-4.670489000	0.728592000
	H	-2.671490000	-4.977279000	-0.309631000
	H	-1.473958000	-5.075032000	1.001610000
	H	-3.239252000	-5.093339000	1.376912000
	P	3.237156000	-0.711642000	0.069809000
	C	3.135758000	0.649433000	-1.127822000
	C	3.679999000	0.490977000	-2.414063000
	C	2.526442000	1.856949000	-0.765028000
	C	3.617324000	1.543118000	-3.326967000
	C	2.457913000	2.906746000	-1.687614000
	C	3.004936000	2.749134000	-2.963733000
	H	4.137936000	-0.453606000	-2.711560000
	H	2.069401000	1.984232000	0.214905000
	H	4.039722000	1.418734000	-4.326753000
	H	1.970863000	3.841920000	-1.397045000
	H	2.951285000	3.570205000	-3.682918000
	C	2.776584000	-2.259150000	-0.775629000
	C	3.704324000	-2.908412000	-1.609773000
	C	1.490379000	-2.795464000	-0.606573000
	C	3.338441000	-4.073403000	-2.285818000
	C	1.135641000	-3.962944000	-1.286872000
	C	2.051252000	-4.599474000	-2.128306000
	H	4.719363000	-2.519288000	-1.715958000
	H	0.735594000	-2.332515000	0.027664000

	H	4.063499000	-4.576119000	-2.930103000
	H	0.128171000	-4.355264000	-1.135995000
	H	1.768686000	-5.513361000	-2.656727000
	C	4.986452000	-0.892317000	0.530202000
	C	5.389916000	-2.051473000	1.213650000
	C	5.912564000	0.128107000	0.274942000
	C	6.713084000	-2.185672000	1.634577000
	C	7.237115000	-0.014230000	0.697130000
	C	7.637498000	-1.167966000	1.375410000
	H	4.670408000	-2.849742000	1.409931000
	H	5.599947000	1.031379000	-0.251739000
	H	7.024729000	-3.088254000	2.164947000
	H	7.957341000	0.781736000	0.495340000
	H	8.673853000	-1.276365000	1.703649000
	C	-5.359546000	-3.465602000	-1.345670000
	C	-6.337365000	-3.274982000	-2.492470000
	H	-5.433523000	-4.505782000	-0.963868000
	H	-4.319880000	-3.353491000	-1.726180000
	H	-6.237079000	-2.265598000	-2.923132000
	H	-6.165586000	-4.014134000	-3.291428000
	H	-7.371858000	-3.383942000	-2.130322000
	O	-5.638651000	-2.530936000	-0.336433000
	H	-4.944697000	-2.623122000	0.365569000
	C	-0.475516000	5.463895000	-1.304788000
	C	-0.469218000	6.755034000	-2.104391000
	H	-0.510158000	4.599475000	-2.004683000
	H	-1.405177000	5.406824000	-0.699731000
	H	-0.458076000	7.622407000	-1.425979000
	H	0.431541000	6.811667000	-2.736279000
	H	-1.356173000	6.826449000	-2.754099000
	O	0.665780000	5.416691000	-0.487308000
	H	0.567018000	4.635591000	0.102814000
INT-2ASol	Ru	-1.649379000	-0.888250000	0.487127000
	C	-2.744988000	-1.411040000	-2.226039000
	O	-3.877832000	-1.168105000	-1.791969000
	O	-1.671498000	-1.450192000	-1.514009000
	C	-2.521530000	-1.645761000	-3.709406000
	H	-2.350014000	-0.664613000	-4.180122000
	H	-1.636588000	-2.270416000	-3.890830000
	H	-3.416439000	-2.093370000	-4.161972000
	C	0.624444000	0.460821000	-0.780940000
	O	0.306479000	-0.452530000	0.081745000
	C	-0.494282000	1.210663000	-1.396759000
	C	-0.496986000	1.570943000	-2.747067000

	C	-1.653914000	1.416348000	-0.619263000
	C	-1.660098000	2.089266000	-3.326409000
	H	0.392750000	1.395917000	-3.354971000
	C	-2.816366000	1.919673000	-1.209951000
	C	-2.825763000	2.243785000	-2.568469000
	H	-1.663592000	2.348012000	-4.388474000
	H	-3.722081000	2.053309000	-0.621290000
	H	-3.750140000	2.599042000	-3.026562000
	C	1.947622000	0.638361000	-1.121642000
	H	2.295544000	1.466405000	-1.732136000
	P	3.142776000	-0.387486000	-0.374767000
	C	4.735146000	0.035418000	-1.126014000
	C	5.480761000	-0.874225000	-1.884877000
	C	5.192658000	1.350265000	-0.924242000
	C	6.695137000	-0.468695000	-2.446808000
	H	5.115052000	-1.891559000	-2.038600000
	C	6.403304000	1.743396000	-1.495589000
	H	4.590934000	2.054508000	-0.337323000
	C	7.154503000	0.836732000	-2.252923000
	H	7.281114000	-1.174577000	-3.039911000
	H	6.760630000	2.765123000	-1.348160000
	H	8.103205000	1.150600000	-2.695046000
	C	2.768869000	-2.150970000	-0.670213000
	C	3.408312000	-3.159205000	0.067040000
	C	1.793906000	-2.494712000	-1.617522000
	C	3.082968000	-4.499364000	-0.151229000
	H	4.147970000	-2.903313000	0.826603000
	C	1.460880000	-3.834747000	-1.820503000
	H	1.273385000	-1.713136000	-2.170239000
	C	2.105601000	-4.838680000	-1.092029000
	H	3.586261000	-5.279320000	0.424684000
	H	0.683603000	-4.089630000	-2.544039000
	H	1.842337000	-5.887029000	-1.252257000
	C	3.408483000	-0.205986000	1.418732000
	C	2.399795000	0.311693000	2.242442000
	C	4.642067000	-0.594954000	1.970001000
	C	2.625881000	0.412750000	3.617932000
	H	1.467561000	0.692411000	1.820439000
	C	4.850019000	-0.502602000	3.346749000
	H	5.449759000	-0.946766000	1.324109000
	C	3.837850000	-0.003958000	4.173715000
	H	1.846827000	0.843163000	4.251967000
	H	5.810020000	-0.804158000	3.772219000
	H	4.005682000	0.079218000	5.250353000

	C	-3.390028000	-2.086076000	1.075960000
	C	-2.169838000	-2.821546000	1.209801000
	C	-1.079877000	-2.318155000	1.984130000
	C	-1.255876000	-1.052187000	2.635903000
	C	-2.436767000	-0.306628000	2.455091000
	C	-3.534559000	-0.811031000	1.672706000
	H	-4.149560000	-2.437186000	0.379540000
	H	-2.029282000	-3.735011000	0.628920000
	H	-0.408579000	-0.583461000	3.137248000
	H	-2.421734000	0.733827000	2.786374000
	C	0.242811000	-3.024492000	2.024206000
	H	1.063096000	-2.300766000	2.117547000
	H	0.282868000	-3.708217000	2.888710000
	H	0.406946000	-3.608886000	1.108641000
	C	-4.779726000	0.016228000	1.397702000
	C	-4.720978000	1.433842000	1.971926000
	C	-6.028859000	-0.722351000	1.906923000
	H	-4.861686000	0.094829000	0.303625000
	H	-3.810725000	1.981148000	1.690185000
	H	-5.590228000	2.005581000	1.613481000
	H	-4.760035000	1.422293000	3.073850000
	H	-6.137526000	-1.717015000	1.448782000
	H	-5.995017000	-0.852659000	3.001459000
	H	-6.932280000	-0.142611000	1.663559000
	H	-1.579628000	1.379956000	0.487528000
	C	-0.411121000	3.011346000	1.991334000
	O	-1.520430000	2.518625000	2.294053000
	O	0.503585000	2.400501000	1.360017000
	C	-0.145007000	4.475905000	2.349869000
	H	-0.722656000	4.773649000	3.236184000
	H	0.928811000	4.656503000	2.504085000
	H	-0.474315000	5.105356000	1.505685000
	C	3.491301000	4.729263000	-1.413804000
	C	2.320802000	4.170328000	-0.620755000
	H	4.033165000	3.926873000	-1.940776000
	H	3.150306000	5.460991000	-2.163825000
	H	4.203951000	5.229187000	-0.737876000
	H	1.745772000	5.010863000	-0.178935000
	H	1.617310000	3.656698000	-1.310951000
	O	2.789029000	3.299310000	0.375507000
	H	1.977111000	2.945371000	0.835399000
	C	-6.913735000	0.303027000	-1.734605000
	C	-7.897496000	1.332493000	-1.206945000
	H	-6.918372000	-0.589535000	-1.076103000

	H	-7.244025000	-0.046265000	-2.735766000
	H	-7.892861000	2.227981000	-1.848432000
	H	-8.921783000	0.927835000	-1.179238000
	H	-7.618305000	1.648414000	-0.188872000
	O	-5.631635000	0.880585000	-1.793963000
	H	-4.977014000	0.163084000	-1.930581000
TS-3ASol	Ru	-1.689987000	-0.734364000	0.511550000
	C	-2.561782000	-1.198443000	-2.322402000
	O	-3.744408000	-1.049566000	-1.988808000
	O	-1.560481000	-1.254316000	-1.516355000
	C	-2.182939000	-1.282857000	-3.791692000
	H	-1.959361000	-0.259735000	-4.135641000
	H	-1.281426000	-1.892870000	-3.941849000
	H	-3.021792000	-1.672912000	-4.383213000
	C	0.630970000	0.568496000	-0.655118000
	O	0.302735000	-0.331477000	0.217124000
	C	-0.482948000	1.372005000	-1.191759000
	C	-0.425297000	2.065941000	-2.401661000
	C	-1.693701000	1.289860000	-0.452170000
	C	-1.582591000	2.661730000	-2.912914000
	H	0.506688000	2.103084000	-2.969462000
	C	-2.840816000	1.892628000	-0.995114000
	C	-2.794213000	2.560816000	-2.219512000
	H	-1.542859000	3.189242000	-3.869515000
	H	-3.793627000	1.834750000	-0.472901000
	H	-3.711810000	2.983529000	-2.633191000
	C	1.948566000	0.673079000	-1.057804000
	H	2.316615000	1.472865000	-1.693382000
	P	3.112556000	-0.436652000	-0.396210000
	C	4.685120000	-0.118167000	-1.237886000
	C	5.305944000	-1.063398000	-2.063044000
	C	5.257112000	1.151951000	-1.043762000
	C	6.507900000	-0.738457000	-2.699250000
	H	4.853459000	-2.046209000	-2.209737000
	C	6.453733000	1.465626000	-1.689064000
	H	4.753273000	1.883741000	-0.402136000
	C	7.079505000	0.523057000	-2.513370000
	H	6.996007000	-1.472729000	-3.344225000
	H	6.898862000	2.453130000	-1.547096000
	H	8.017878000	0.774200000	-3.013826000
	C	2.616495000	-2.173253000	-0.660333000
	C	3.279565000	-3.217558000	0.002676000
	C	1.534151000	-2.457744000	-1.504668000
	C	2.872193000	-4.538627000	-0.192203000

	H	4.102949000	-3.002772000	0.685734000
	C	1.118779000	-3.778784000	-1.679458000
	H	0.987420000	-1.650934000	-1.990836000
	C	1.788528000	-4.820203000	-1.030911000
	H	3.393964000	-5.348437000	0.322864000
	H	0.255940000	-3.985208000	-2.316112000
	H	1.461336000	-5.853425000	-1.170760000
	C	3.494663000	-0.276162000	1.381346000
	C	2.539056000	0.246490000	2.264296000
	C	4.750364000	-0.681555000	1.862860000
	C	2.841128000	0.344073000	3.624293000
	H	1.584194000	0.607844000	1.886369000
	C	5.038219000	-0.589617000	3.225793000
	H	5.513015000	-1.048772000	1.172715000
	C	4.081423000	-0.080069000	4.108919000
	H	2.101273000	0.770611000	4.305885000
	H	6.017008000	-0.903317000	3.596073000
	H	4.310927000	0.000733000	5.174215000
	C	-3.388118000	-2.067663000	0.878098000
	C	-2.168093000	-2.786286000	1.103307000
	C	-1.176832000	-2.286674000	1.982527000
	C	-1.450328000	-1.048215000	2.659954000
	C	-2.660687000	-0.355129000	2.445372000
	C	-3.666840000	-0.853640000	1.549590000
	H	-4.064193000	-2.403118000	0.092888000
	H	-1.945837000	-3.665024000	0.496131000
	H	-0.671929000	-0.585150000	3.267869000
	H	-2.767216000	0.631869000	2.887993000
	C	0.164929000	-2.944431000	2.116120000
	H	0.956814000	-2.186965000	2.192667000
	H	0.198305000	-3.570451000	3.023522000
	H	0.384691000	-3.576422000	1.245464000
	C	-4.953248000	-0.099361000	1.261301000
	C	-5.007983000	1.288148000	1.906105000
	C	-6.170612000	-0.937896000	1.683378000
	H	-4.998910000	0.033088000	0.170080000
	H	-4.121630000	1.896377000	1.677932000
	H	-5.892033000	1.829502000	1.537376000
	H	-5.091347000	1.219898000	3.003797000
	H	-6.188442000	-1.915561000	1.178673000
	H	-6.172419000	-1.117218000	2.771665000
	H	-7.102462000	-0.411490000	1.424904000
	H	-1.557774000	1.509690000	0.806735000
	C	-0.325094000	2.878745000	2.064321000

	O	-1.460694000	2.299850000	1.956988000
	O	0.691725000	2.551874000	1.426904000
	C	-0.265936000	4.061185000	3.021880000
	H	-1.006753000	3.959286000	3.826852000
	H	0.747051000	4.170785000	3.433562000
	H	-0.504997000	4.977545000	2.456847000
	C	3.821270000	4.628496000	-1.367539000
	C	2.675166000	4.183521000	-0.474694000
	H	4.207287000	3.788021000	-1.966586000
	H	3.496916000	5.421671000	-2.059893000
	H	4.652128000	5.018057000	-0.757137000
	H	2.250457000	5.067956000	0.044468000
	H	1.853928000	3.772176000	-1.096358000
	O	3.136388000	3.229385000	0.452049000
	H	2.342774000	2.935317000	0.951408000
	C	-6.848807000	0.216684000	-1.983116000
	C	-7.943103000	1.137992000	-1.472758000
	H	-6.833834000	-0.714368000	-1.379654000
	H	-7.079813000	-0.091445000	-3.024767000
	H	-7.960358000	2.070194000	-2.059412000
	H	-8.933300000	0.660442000	-1.545260000
	H	-7.761843000	1.408399000	-0.419875000
	O	-5.614332000	0.887077000	-1.911497000
	H	-4.900412000	0.231707000	-2.067736000
INT-3ASol	Ru	1.453385000	-0.725848000	-0.033957000
	C	2.253472000	1.559302000	1.740156000
	O	3.448129000	1.274847000	1.581843000
	O	1.255688000	0.855582000	1.340430000
	C	1.865959000	2.863371000	2.421966000
	H	1.780766000	3.636715000	1.640496000
	H	0.891979000	2.785991000	2.925574000
	H	2.645888000	3.174353000	3.130043000
	C	-0.749917000	0.863284000	-1.041867000
	O	-0.523241000	-0.285276000	-0.493391000
	C	0.430448000	1.534019000	-1.595396000
	C	0.384839000	2.737711000	-2.317663000
	C	1.661741000	0.889080000	-1.290140000
	C	1.567820000	3.337514000	-2.740730000
	H	-0.576961000	3.206912000	-2.541393000
	C	2.836836000	1.538613000	-1.697034000
	C	2.791988000	2.738819000	-2.413111000
	H	1.542024000	4.273311000	-3.304313000
	H	3.814207000	1.152619000	-1.417448000
	H	3.731173000	3.219174000	-2.700761000

	C	-2.053326000	1.364090000	-1.018367000
	H	-2.337443000	2.329308000	-1.431722000
	P	-3.268750000	0.443775000	-0.188054000
	C	-4.728991000	1.509569000	-0.005687000
	C	-5.197643000	1.895067000	1.257050000
	C	-5.371621000	1.977733000	-1.164395000
	C	-6.302659000	2.745041000	1.359427000
	H	-4.698075000	1.533586000	2.157475000
	C	-6.472741000	2.826831000	-1.055398000
	H	-5.011954000	1.672595000	-2.149898000
	C	-6.939365000	3.210716000	0.206735000
	H	-6.664783000	3.044904000	2.345360000
	H	-6.969968000	3.189708000	-1.957782000
	H	-7.802818000	3.874847000	0.290011000
	C	-2.781540000	-0.137842000	1.467230000
	C	-3.483009000	-1.183601000	2.087663000
	C	-1.676261000	0.442001000	2.102187000
	C	-3.085459000	-1.635416000	3.347019000
	H	-4.327645000	-1.657850000	1.584767000
	C	-1.270206000	-0.031225000	3.350749000
	H	-1.091796000	1.216830000	1.608580000
	C	-1.975385000	-1.062129000	3.977207000
	H	-3.633966000	-2.447568000	3.829715000
	H	-0.381186000	0.399095000	3.815017000
	H	-1.653700000	-1.430390000	4.954560000
	C	-3.854210000	-1.044180000	-1.077255000
	C	-2.932642000	-1.803042000	-1.818124000
	C	-5.197660000	-1.444319000	-1.013403000
	C	-3.359045000	-2.955277000	-2.480319000
	H	-1.888644000	-1.490324000	-1.857987000
	C	-5.615670000	-2.599901000	-1.678918000
	H	-5.921866000	-0.850194000	-0.452341000
	C	-4.697173000	-3.356759000	-2.410737000
	H	-2.640694000	-3.543298000	-3.056369000
	H	-6.663236000	-2.905855000	-1.628438000
	H	-5.025501000	-4.258860000	-2.932447000
	C	2.998184000	-1.749244000	1.129182000
	C	1.763633000	-2.368208000	1.524667000
	C	0.870776000	-2.850660000	0.557694000
	C	1.258303000	-2.736063000	-0.827516000
	C	2.521533000	-2.216277000	-1.204569000
	C	3.443299000	-1.730102000	-0.217819000
	H	3.608732000	-1.255794000	1.884071000
	H	1.459312000	-2.338776000	2.571934000

	H	0.548450000	-3.030759000	-1.602952000
	H	2.766039000	-2.145685000	-2.263046000
	C	-0.507432000	-3.332932000	0.909525000
	H	-1.261435000	-2.771866000	0.337834000
	H	-0.625390000	-4.402837000	0.669927000
	H	-0.721900000	-3.185821000	1.976836000
	C	4.823213000	-1.191478000	-0.553018000
	C	5.124152000	-1.153022000	-2.052423000
	C	5.907688000	-1.998939000	0.179860000
	H	4.858787000	-0.160847000	-0.170960000
	H	4.373090000	-0.580004000	-2.613155000
	H	6.097845000	-0.669032000	-2.221342000
	H	5.175430000	-2.168626000	-2.480298000
	H	5.759995000	-1.987424000	1.269740000
	H	5.912051000	-3.051545000	-0.150468000
	H	6.902639000	-1.574392000	-0.026495000
	C	6.648793000	1.745765000	0.889771000
	C	7.888040000	1.666681000	0.014621000
	H	6.509417000	0.784450000	1.426267000
	H	6.799717000	2.518515000	1.673750000
	H	8.027831000	2.612726000	-0.532260000
	H	8.790829000	1.472598000	0.615876000
	H	7.785043000	0.861636000	-0.731031000
	O	5.536702000	2.045618000	0.084740000
	H	4.729242000	1.914846000	0.628121000
TS-4ASol	C	1.355556000	2.172641000	0.884638000
	C	2.522410000	2.098232000	0.064951000
	C	3.756345000	1.715980000	0.708655000
	C	3.767381000	1.264089000	2.004470000
	C	2.555343000	1.202419000	2.782136000
	C	1.394467000	1.754370000	2.256575000
	H	0.443677000	2.634006000	0.508195000
	H	4.677899000	1.688617000	0.123836000
	H	4.690361000	0.867909000	2.431314000
	H	0.482541000	1.797865000	2.855752000
	C	2.598001000	0.580329000	4.149168000
	H	1.606998000	0.574610000	4.625042000
	H	2.955851000	-0.459360000	4.076098000
	H	3.300589000	1.118941000	4.807279000
	C	2.594076000	2.735895000	-1.316068000
	H	3.195155000	2.054278000	-1.941392000
	C	3.331546000	4.083252000	-1.239163000
	H	4.335461000	3.973992000	-0.801630000
	H	3.445640000	4.526637000	-2.241589000

	H	2.770252000	4.797034000	-0.612968000
	C	1.235200000	2.906156000	-1.997326000
	H	1.370405000	3.275280000	-3.025639000
	H	0.690033000	1.955463000	-2.046954000
	H	0.607422000	3.642329000	-1.467657000
	Ru	1.571907000	0.087575000	0.393123000
	C	2.495913000	-2.365850000	1.009954000
	O	3.428258000	-1.532600000	1.106153000
	O	1.305184000	-1.985847000	0.763955000
	C	2.763061000	-3.846095000	1.142700000
	H	3.590015000	-4.025427000	1.843275000
	H	1.859912000	-4.385981000	1.457886000
	H	3.064022000	-4.230330000	0.154222000
	C	-0.676506000	-0.396439000	-1.287239000
	O	-0.373453000	0.224014000	-0.186711000
	C	0.467382000	-0.747784000	-2.128892000
	C	0.401997000	-1.254791000	-3.436215000
	C	1.714652000	-0.488104000	-1.497621000
	C	1.579547000	-1.498314000	-4.139827000
	H	-0.566835000	-1.450245000	-3.904342000
	C	2.890567000	-0.747255000	-2.222825000
	C	2.817959000	-1.241521000	-3.528108000
	H	1.540561000	-1.886427000	-5.160415000
	H	3.861148000	-0.572947000	-1.752468000
	H	3.742051000	-1.436279000	-4.081022000
	C	-2.021735000	-0.670434000	-1.543786000
	H	-2.368865000	-1.190275000	-2.434050000
	P	-3.181552000	-0.255922000	-0.321660000
	C	-4.749402000	-1.071269000	-0.744200000
	C	-5.311124000	-2.047847000	0.088709000
	C	-5.387384000	-0.725459000	-1.947461000
	C	-6.504089000	-2.675345000	-0.281376000
	H	-4.815719000	-2.318046000	1.022903000
	C	-6.576106000	-1.357384000	-2.312316000
	H	-4.955067000	0.042002000	-2.593916000
	C	-7.135808000	-2.332247000	-1.478980000
	H	-6.939345000	-3.436695000	0.369840000
	H	-7.069315000	-1.087279000	-3.248807000
	H	-8.068076000	-2.824608000	-1.765417000
	C	-3.573969000	1.525864000	-0.209052000
	C	-4.856732000	1.965593000	0.151898000
	C	-2.564776000	2.462677000	-0.484335000
	C	-5.124902000	3.333794000	0.247292000
	H	-5.650840000	1.242007000	0.348365000

	C	-2.840955000	3.827095000	-0.388467000
	H	-1.569470000	2.116627000	-0.761410000
	C	-4.117615000	4.264685000	-0.020532000
	H	-6.125549000	3.672285000	0.526065000
	H	-2.052591000	4.551939000	-0.604668000
	H	-4.329532000	5.334112000	0.051581000
	C	-2.702607000	-0.781623000	1.354140000
	C	-1.736704000	-1.784747000	1.506847000
	C	-3.269323000	-0.171386000	2.483552000
	C	-1.337272000	-2.171182000	2.786273000
	H	-1.258297000	-2.229822000	0.635248000
	C	-2.877882000	-0.576052000	3.761122000
	H	-4.001141000	0.630219000	2.369686000
	C	-1.908994000	-1.573617000	3.912714000
	H	-0.553529000	-2.922989000	2.891625000
	H	-3.320007000	-0.100318000	4.639625000
	H	-1.589960000	-1.875600000	4.913338000
	C	6.262899000	-1.732177000	-0.746572000
	C	7.381462000	-1.266109000	-1.661506000
	H	5.552266000	-2.364802000	-1.319045000
	H	6.682131000	-2.377342000	0.054335000
	H	7.932762000	-2.120987000	-2.084678000
	H	6.975937000	-0.666379000	-2.492123000
	H	8.090192000	-0.633126000	-1.104073000
	O	5.615428000	-0.606848000	-0.203875000
	H	4.855625000	-0.925357000	0.326539000
INT-4ASol	C	1.471903000	2.249747000	-0.095431000
	C	2.552262000	1.797216000	-0.914067000
	C	3.873053000	1.852693000	-0.342441000
	C	4.090906000	2.246345000	0.951805000
	C	2.998270000	2.625044000	1.795957000
	C	1.722785000	2.662975000	1.258201000
	H	0.505200000	2.488172000	-0.537278000
	H	4.719875000	1.529961000	-0.951457000
	H	5.102492000	2.224978000	1.363132000
	H	0.885137000	3.014151000	1.866150000
	C	3.264931000	2.971396000	3.234402000
	H	2.357738000	3.323846000	3.746385000
	H	3.643313000	2.087992000	3.776298000
	H	4.038668000	3.752654000	3.318041000
	C	2.455005000	1.722295000	-2.437373000
	H	2.956260000	0.788933000	-2.743575000
	C	3.217362000	2.898165000	-3.074839000
	H	4.273438000	2.922146000	-2.769249000

	H	3.186035000	2.830279000	-4.174440000
	H	2.762619000	3.859243000	-2.781045000
	C	1.026888000	1.683424000	-2.982204000
	H	1.044337000	1.543489000	-4.073979000
	H	0.445797000	0.865151000	-2.545950000
	H	0.496342000	2.629751000	-2.783179000
	Ru	1.554450000	0.134976000	0.342671000
	C	2.660959000	-1.567593000	1.875655000
	O	3.334096000	-0.595598000	1.399044000
	O	1.422104000	-1.611456000	1.624419000
	C	3.333660000	-2.648704000	2.672280000
	H	4.186957000	-2.240791000	3.230824000
	H	2.617540000	-3.132677000	3.349370000
	H	3.715544000	-3.409512000	1.972276000
	C	-0.760634000	-0.848726000	-1.018689000
	O	-0.422371000	0.161287000	-0.278553000
	C	0.358698000	-1.571319000	-1.629918000
	C	0.253281000	-2.553107000	-2.627652000
	C	1.627949000	-1.149129000	-1.146065000
	C	1.406862000	-3.115767000	-3.169778000
	H	-0.730176000	-2.865101000	-2.989938000
	C	2.780617000	-1.725998000	-1.709003000
	C	2.665397000	-2.695024000	-2.709033000
	H	1.333434000	-3.874537000	-3.952423000
	H	3.765748000	-1.408325000	-1.361123000
	H	3.571124000	-3.133396000	-3.139087000
	C	-2.113831000	-1.174847000	-1.143792000
	H	-2.473674000	-2.014646000	-1.734318000
	P	-3.250408000	-0.327992000	-0.144898000
	C	-4.824082000	-1.233537000	-0.227901000
	C	-5.406725000	-1.796281000	0.915067000
	C	-5.447685000	-1.384080000	-1.478230000
	C	-6.605729000	-2.506878000	0.807053000
	H	-4.922365000	-1.681387000	1.886273000
	C	-6.642670000	-2.096065000	-1.579641000
	H	-4.997957000	-0.939813000	-2.369341000
	C	-7.223071000	-2.657536000	-0.436767000
	H	-7.056642000	-2.945697000	1.699967000
	H	-7.124435000	-2.212319000	-2.553141000
	H	-8.159863000	-3.213787000	-0.518034000
	C	-3.643093000	1.380418000	-0.660658000
	C	-4.940518000	1.898500000	-0.526708000
	C	-2.621063000	2.181255000	-1.196629000
	C	-5.211988000	3.211639000	-0.920668000

	H	-5.743070000	1.276607000	-0.124740000
	C	-2.901507000	3.490246000	-1.589664000
	H	-1.614660000	1.773382000	-1.288451000
	C	-4.193751000	4.007892000	-1.451466000
	H	-6.223791000	3.610540000	-0.816893000
	H	-2.103666000	4.108846000	-2.007301000
	H	-4.408423000	5.033216000	-1.762105000
	C	-2.742820000	-0.232765000	1.600829000
	C	-1.789269000	-1.137818000	2.085758000
	C	-3.283943000	0.742522000	2.451633000
	C	-1.377031000	-1.063953000	3.416213000
	H	-1.340308000	-1.873510000	1.418449000
	C	-2.880500000	0.799545000	3.787060000
	H	-4.006984000	1.466406000	2.070946000
	C	-1.924794000	-0.101080000	4.268586000
	H	-0.603906000	-1.747844000	3.770075000
	H	-3.302904000	1.559119000	4.448994000
	H	-1.597094000	-0.042384000	5.309412000
	C	6.117057000	-2.014309000	0.106648000
	C	7.303911000	-2.244660000	-0.811522000
	H	5.324225000	-2.759102000	-0.118989000
	H	6.423122000	-2.189738000	1.160154000
	H	7.715730000	-3.258497000	-0.684155000
	H	7.005000000	-2.118261000	-1.864228000
	H	8.098420000	-1.512495000	-0.596914000
	O	5.646529000	-0.700946000	-0.074839000
	H	4.870979000	-0.573250000	0.507921000

Table S2. Cartesian Coordinates of the optimized stationary points in the remaning steps after C-H activation, calculated at the B3LYP+D3BJ/def2SVP level

Names	Cartesian Coordinates			
	C	-0.025708000	-1.044170000	0.000661000
	C	-0.628384000	0.224981000	-0.000502000
	C	0.214320000	1.342097000	-0.001375000
	C	1.605757000	1.202488000	-0.001121000
	C	2.205881000	-0.062117000	0.000033000
p-cymene	C	1.360534000	-1.184203000	0.000914000
	H	-0.650133000	-1.941443000	0.001370000
	H	-0.225042000	2.343766000	-0.002300000
	H	2.236071000	2.096250000	-0.001841000
	H	1.799555000	-2.186376000	0.001791000
	C	3.704639000	-0.227508000	0.000418000

	H	4.045882000	-0.789335000	-0.885120000
	H	4.218210000	0.745097000	-0.000153000
	H	4.045506000	-0.788112000	0.886865000
	C	-2.139942000	0.381462000	-0.000606000
	H	-2.349855000	1.465044000	-0.002389000
	C	-2.772942000	-0.209589000	1.268220000
	H	-2.340617000	0.240028000	2.175352000
	H	-3.861106000	-0.035007000	1.281942000
	H	-2.609532000	-1.298212000	1.324269000
	C	-2.773441000	-0.213850000	-1.267165000
	H	-3.861591000	-0.039198000	-1.281061000
	H	-2.341415000	0.232626000	-2.175991000
	H	-2.610219000	-1.302680000	-1.319584000
INT-4A-1	Ru	-1.864602000	-0.360602000	0.306703000
	C	-3.129773000	-1.235086000	2.461029000
	O	-3.704504000	-0.780466000	1.408442000
	O	-1.878089000	-1.247520000	2.540313000
	C	-3.990081000	-1.788881000	3.570727000
	H	-3.450899000	-1.757727000	4.526701000
	H	-4.937140000	-1.235932000	3.640789000
	H	-4.226511000	-2.841169000	3.341695000
	C	0.555002000	-1.439299000	-0.848650000
	O	0.090018000	-0.295098000	-0.465281000
	C	-0.443056000	-2.510287000	-0.998292000
	C	-0.179525000	-3.772518000	-1.551244000
	C	-1.722015000	-2.184167000	-0.441946000
	C	-1.170318000	-4.752057000	-1.569774000
	H	0.806962000	-3.988022000	-1.971279000
	C	-2.687523000	-3.217104000	-0.439883000
	C	-2.419948000	-4.469197000	-0.996668000
	H	-0.971506000	-5.732543000	-2.008670000
	H	-3.656336000	-3.038342000	0.032286000
	H	-3.192242000	-5.244835000	-0.978023000
	C	1.938626000	-1.607784000	-0.951280000
	H	2.404223000	-2.551965000	-1.225497000
	P	2.960577000	-0.376492000	-0.275275000
	C	4.624350000	-1.095904000	-0.127095000
	C	5.246948000	-1.256787000	1.117600000
	C	5.277832000	-1.522381000	-1.295746000
	C	6.515032000	-1.840601000	1.192036000
	H	4.740511000	-0.927783000	2.026574000
	C	6.542389000	-2.105396000	-1.214926000
	H	4.796088000	-1.392142000	-2.267687000
	C	7.162583000	-2.264402000	0.029329000

	H	6.996406000	-1.965268000	2.164703000
	H	7.047265000	-2.435618000	-2.125653000
	H	8.153787000	-2.719889000	0.090803000
	C	2.438955000	0.185687000	1.378385000
	C	2.982161000	1.360934000	1.920471000
	C	1.479384000	-0.538411000	2.098717000
	C	2.575542000	1.795496000	3.182662000
	H	3.710940000	1.943367000	1.353291000
	C	1.042282000	-0.074830000	3.340179000
	H	1.045350000	-1.446364000	1.681960000
	C	1.596581000	1.085855000	3.885404000
	H	3.004713000	2.706768000	3.606171000
	H	0.241133000	-0.613589000	3.848118000
	H	1.252829000	1.449707000	4.856744000
	C	3.163941000	1.140225000	-1.269315000
	C	2.035877000	1.671423000	-1.913532000
	C	4.400447000	1.798599000	-1.362885000
	C	2.143694000	2.859786000	-2.637126000
	H	1.073557000	1.168656000	-1.827911000
	C	4.502043000	2.984651000	-2.094794000
	H	5.283755000	1.386664000	-0.870651000
	C	3.374777000	3.516907000	-2.728552000
	H	1.246581000	3.264158000	-3.109590000
	H	5.465688000	3.494537000	-2.168394000
	H	3.457973000	4.447548000	-3.295458000
	C	-0.139774000	3.407864000	1.751436000
	C	-0.391717000	3.037430000	0.438933000
	C	-1.598533000	2.386826000	0.097295000
	C	-2.574795000	2.178041000	1.099935000
	C	-2.298220000	2.548942000	2.425010000
	C	-1.086861000	3.148611000	2.755202000
	H	0.810180000	3.880537000	2.007497000
	H	0.347761000	3.204389000	-0.343454000
	H	-3.561937000	1.798243000	0.848261000
	H	-3.053686000	2.370605000	3.193469000
	H	-0.874376000	3.423744000	3.790898000
	C	-1.734243000	1.882122000	-1.307021000
	O	-1.095395000	2.352738000	-2.237574000
	C	-2.437329000	0.533450000	-1.448503000
	H	-2.087053000	0.066471000	-2.387482000
	S	-4.194581000	0.698451000	-1.743409000
	C	-4.944232000	-0.888385000	-1.417602000
	H	-5.990720000	-0.825346000	-1.744246000
	H	-4.374464000	-1.666381000	-1.941820000

	H	-4.845992000	-1.018924000	-0.327683000
	O	-4.894241000	1.769343000	-1.002485000
	C	-4.428173000	0.975767000	-3.516182000
	H	-4.064663000	0.105658000	-4.078300000
	H	-5.497412000	1.159847000	-3.685472000
	H	-3.829923000	1.866800000	-3.751286000
TS-5A	Ru	1.809145000	0.382564000	0.294147000
	C	3.035236000	1.171645000	2.504947000
	O	3.607004000	0.762164000	1.429423000
	O	1.786751000	1.183262000	2.591024000
	C	3.909754000	1.669264000	3.630097000
	H	3.358027000	1.658354000	4.579004000
	H	4.824147000	1.063977000	3.705922000
	H	4.209884000	2.707393000	3.411967000
	C	-0.617732000	1.516925000	-0.762560000
	O	-0.152389000	0.364343000	-0.395093000
	C	0.385898000	2.579969000	-0.915843000
	C	0.124386000	3.857653000	-1.432403000
	C	1.679397000	2.221575000	-0.419857000
	C	1.135048000	4.816136000	-1.474094000
	H	-0.874050000	4.101410000	-1.806002000
	C	2.669072000	3.226880000	-0.446027000
	C	2.403721000	4.495176000	-0.967473000
	H	0.938372000	5.809543000	-1.883897000
	H	3.652824000	3.016306000	-0.021727000
	H	3.193346000	5.253220000	-0.972923000
	C	-2.000224000	1.672486000	-0.863650000
	H	-2.481613000	2.612518000	-1.124236000
	P	-3.000829000	0.381578000	-0.264477000
	C	-4.689794000	1.041782000	-0.131918000
	C	-5.340417000	1.151627000	1.103902000
	C	-5.334464000	1.475123000	-1.302887000
	C	-6.628391000	1.691625000	1.167232000
	H	-4.840613000	0.816716000	2.014435000
	C	-6.618926000	2.014292000	-1.233029000
	H	-4.830618000	1.383920000	-2.268063000
	C	-7.267352000	2.122311000	0.002314000
	H	-7.132250000	1.776152000	2.132759000
	H	-7.117408000	2.349626000	-2.145386000
	H	-8.274275000	2.543056000	0.054946000
	C	-2.502538000	-0.230000000	1.378707000
	C	-3.012735000	-1.446003000	1.858595000
	C	-1.580949000	0.494074000	2.147125000
	C	-2.610630000	-1.922431000	3.107158000

	H	-3.710244000	-2.028556000	1.253511000
	C	-1.149449000	-0.007775000	3.375337000
	H	-1.170901000	1.432402000	1.775345000
	C	-1.669910000	-1.211085000	3.858328000
	H	-3.012898000	-2.866781000	3.481681000
	H	-0.376768000	0.534336000	3.922216000
	H	-1.329032000	-1.606205000	4.818359000
	C	-3.128001000	-1.094140000	-1.330855000
	C	-1.979661000	-1.546740000	-1.999447000
	C	-4.335223000	-1.800754000	-1.454453000
	C	-2.038015000	-2.704319000	-2.777038000
	H	-1.038355000	-1.010583000	-1.889898000
	C	-4.387263000	-2.955771000	-2.238944000
	H	-5.234851000	-1.449261000	-0.945109000
	C	-3.239641000	-3.409328000	-2.897026000
	H	-1.124539000	-3.048376000	-3.265800000
	H	-5.328194000	-3.502867000	-2.335260000
	H	-3.283778000	-4.316275000	-3.505175000
	C	0.090289000	-3.484016000	1.550462000
	C	0.389092000	-3.057750000	0.264195000
	C	1.592875000	-2.369256000	0.002527000
	C	2.522448000	-2.182226000	1.050486000
	C	2.201582000	-2.610807000	2.347555000
	C	0.989177000	-3.245296000	2.602078000
	H	-0.858580000	-3.986360000	1.748333000
	H	-0.312488000	-3.208646000	-0.555403000
	H	3.511301000	-1.774252000	0.851736000
	H	2.920967000	-2.450634000	3.153677000
	H	0.738891000	-3.564392000	3.616485000
	C	1.786473000	-1.782632000	-1.364569000
	O	1.222360000	-2.228413000	-2.357313000
	C	2.425692000	-0.413666000	-1.371868000
	H	2.298604000	0.056961000	-2.363974000
	S	4.456277000	-0.671839000	-1.594840000
	C	5.220440000	0.901518000	-1.187349000
	H	6.294704000	0.817236000	-1.400148000
	H	4.731654000	1.694715000	-1.767884000
	H	5.013109000	1.035296000	-0.114537000
	O	5.127569000	-1.772718000	-0.848411000
	C	4.808352000	-0.871362000	-3.363695000
	H	4.450819000	0.010636000	-3.911472000
	H	5.891500000	-1.016305000	-3.478891000
	H	4.256866000	-1.768690000	-3.675861000
INT-5A	Ru	2.355207000	0.229573000	-0.288785000

	C	3.927382000	0.508348000	1.810853000
	O	4.276791000	0.318533000	0.589052000
	O	2.714880000	0.529306000	2.117598000
	C	5.015941000	0.736324000	2.829063000
	H	5.872956000	0.076885000	2.632026000
	H	5.368757000	1.776587000	2.740020000
	H	4.629429000	0.579407000	3.844235000
	C	-0.064293000	1.723911000	-0.365583000
	O	0.370142000	0.484539000	-0.286162000
	C	0.975349000	2.729266000	-0.592164000
	C	0.754752000	4.103657000	-0.767690000
	C	2.291567000	2.181948000	-0.624581000
	C	1.837103000	4.953316000	-0.985341000
	H	-0.261579000	4.505801000	-0.738596000
	C	3.365817000	3.057509000	-0.843077000
	C	3.137063000	4.424841000	-1.022176000
	H	1.675462000	6.024262000	-1.127308000
	H	4.383829000	2.661736000	-0.860143000
	H	3.984649000	5.095448000	-1.191785000
	C	-1.421873000	1.917591000	-0.194832000
	H	-1.903558000	2.892976000	-0.206200000
	P	-2.397721000	0.495257000	0.114156000
	C	-4.054547000	1.087627000	0.566952000
	C	-4.591594000	0.859067000	1.840627000
	C	-4.787950000	1.820869000	-0.381510000
	C	-5.856125000	1.360699000	2.162560000
	H	-4.023217000	0.291317000	2.579199000
	C	-6.048403000	2.319332000	-0.053474000
	H	-4.374358000	1.994264000	-1.377839000
	C	-6.583671000	2.089138000	1.218886000
	H	-6.271751000	1.180970000	3.156542000
	H	-6.616457000	2.888024000	-0.793074000
	H	-7.571816000	2.479319000	1.473461000
	C	-1.775058000	-0.541110000	1.473990000
	C	-2.285141000	-1.836406000	1.649808000
	C	-0.759075000	-0.066401000	2.314347000
	C	-1.788966000	-2.643403000	2.674432000
	H	-3.055259000	-2.222622000	0.979115000
	C	-0.241978000	-0.891067000	3.313227000
	H	-0.344132000	0.930141000	2.169361000
	C	-0.761222000	-2.174985000	3.498792000
	H	-2.189363000	-3.650366000	2.812926000
	H	0.590185000	-0.528508000	3.918848000
	H	-0.353018000	-2.820681000	4.279976000

	C	-2.628112000	-0.600548000	-1.327086000
	C	-1.559003000	-0.828360000	-2.209528000
	C	-3.856179000	-1.250284000	-1.536072000
	C	-1.715552000	-1.704402000	-3.284734000
	H	-0.599658000	-0.342681000	-2.042322000
	C	-4.006304000	-2.124151000	-2.615626000
	H	-4.697986000	-1.070130000	-0.864531000
	C	-2.937355000	-2.353545000	-3.487394000
	H	-0.860583000	-1.884239000	-3.940123000
	H	-4.963603000	-2.625746000	-2.775761000
	H	-3.057723000	-3.040796000	-4.328273000
	C	2.716767000	-3.180175000	1.126860000
	C	2.969373000	-2.492814000	-0.068978000
	C	1.986116000	-2.452336000	-1.078585000
	C	0.785041000	-3.164001000	-0.899063000
	C	0.543043000	-3.840525000	0.290712000
	C	1.503337000	-3.838093000	1.313036000
	H	3.479506000	-3.198148000	1.908403000
	H	3.939364000	-2.019189000	-0.223950000
	H	0.042215000	-3.142558000	-1.696014000
	H	-0.406036000	-4.360517000	0.437282000
	H	1.300375000	-4.357242000	2.252354000
	C	2.146653000	-1.586717000	-2.304660000
	O	1.634222000	-1.881823000	-3.378432000
	C	2.704841000	-0.241588000	-2.022547000
	H	3.025321000	0.376557000	-2.876728000
TS-6A	Ru	1.927060000	-1.289471000	-0.182013000
	C	3.194181000	-2.157233000	-2.179876000
	O	3.765379000	-2.083247000	-1.056679000
	O	1.979128000	-1.761459000	-2.266962000
	C	3.897953000	-2.701808000	-3.391256000
	H	3.791483000	-1.997643000	-4.229974000
	H	4.958089000	-2.882832000	-3.175061000
	H	3.416947000	-3.645519000	-3.693759000
	C	-0.419763000	-0.687192000	1.410423000
	O	0.038998000	-0.645469000	0.197977000
	C	0.517029000	-1.195657000	2.419564000
	C	0.166704000	-1.499347000	3.738078000
	C	1.830919000	-1.482974000	1.924993000
	C	1.092407000	-2.095377000	4.594772000
	H	-0.845255000	-1.289334000	4.092536000
	C	2.740065000	-2.099906000	2.812674000
	C	2.376113000	-2.405132000	4.121860000
	H	0.814011000	-2.331764000	5.624249000

	H	3.746013000	-2.336773000	2.457115000
	H	3.097604000	-2.889858000	4.784960000
	C	-1.738432000	-0.281071000	1.605962000
	H	-2.258667000	-0.311670000	2.559329000
	P	-2.579077000	0.108915000	0.130775000
	C	-4.295676000	0.555249000	0.535485000
	C	-5.341626000	0.327269000	-0.372075000
	C	-4.557391000	1.233627000	1.736921000
	C	-6.636291000	0.754998000	-0.070473000
	H	-5.148519000	-0.191889000	-1.312188000
	C	-5.853555000	1.657826000	2.033669000
	H	-3.743997000	1.439394000	2.435095000
	C	-6.894807000	1.417437000	1.132426000
	H	-7.446209000	0.569004000	-0.779495000
	H	-6.049795000	2.181964000	2.971732000
	H	-7.908663000	1.750273000	1.366632000
	C	-2.609564000	-1.285146000	-1.043065000
	C	-1.630339000	-1.378734000	-2.043337000
	C	-3.488957000	-2.357610000	-0.823528000
	C	-1.543443000	-2.530068000	-2.827330000
	H	-0.912620000	-0.572034000	-2.184242000
	C	-3.408987000	-3.499012000	-1.622396000
	H	-4.228793000	-2.307450000	-0.021565000
	C	-2.434973000	-3.586523000	-2.623170000
	H	-0.753304000	-2.602383000	-3.577078000
	H	-4.099512000	-4.328612000	-1.453697000
	H	-2.363672000	-4.488407000	-3.236032000
	C	-1.896467000	1.536963000	-0.770192000
	C	-0.864469000	2.291604000	-0.200796000
	C	-2.417024000	1.882498000	-2.026514000
	C	-0.340869000	3.380909000	-0.901774000
	H	-0.453301000	2.019935000	0.772244000
	C	-1.895861000	2.977867000	-2.715922000
	H	-3.220031000	1.292562000	-2.474587000
	C	-0.853680000	3.724185000	-2.155299000
	H	0.484703000	3.948496000	-0.468852000
	H	-2.298763000	3.245006000	-3.695652000
	H	-0.436821000	4.573856000	-2.701447000
	C	4.583867000	3.078514000	-1.220848000
	C	3.919641000	2.044843000	-0.557228000
	C	3.062110000	2.335248000	0.515782000
	C	2.884024000	3.669054000	0.916757000
	C	3.550580000	4.698803000	0.255038000
	C	4.401229000	4.404523000	-0.817461000

	H	5.242819000	2.848164000	-2.061334000
	H	4.042056000	1.010910000	-0.885555000
	H	2.210698000	3.869648000	1.752260000
	H	3.410244000	5.734818000	0.573445000
	H	4.921807000	5.211304000	-1.339562000
	C	2.298121000	1.268977000	1.252044000
	O	1.364102000	1.570200000	1.981058000
	C	2.761872000	-0.124306000	0.995149000
	H	3.818240000	-0.296169000	1.269904000
INT-6A	Ru	-2.361065000	-0.707685000	0.240664000
	C	-3.413118000	-2.674232000	1.440374000
	O	-4.058611000	-1.931573000	0.634646000
	O	-2.193503000	-2.406416000	1.669588000
	C	-4.074445000	-3.870086000	2.068425000
	H	-3.676277000	-4.040604000	3.078105000
	H	-5.164205000	-3.739885000	2.094093000
	H	-3.844213000	-4.759008000	1.458202000
	C	-0.145418000	-0.496780000	-0.979391000
	O	-0.482312000	0.191710000	0.058658000
	C	-1.326488000	-1.176246000	-1.598460000
	C	-1.169188000	-2.511983000	-2.121985000
	C	-2.605613000	-0.486610000	-1.833125000
	C	-2.182150000	-3.149718000	-2.778800000
	H	-0.210272000	-3.009602000	-1.964345000
	C	-3.637166000	-1.221983000	-2.531059000
	C	-3.438815000	-2.493339000	-2.982735000
	H	-2.038924000	-4.166872000	-3.151278000
	H	-4.591072000	-0.716138000	-2.693805000
	H	-4.239793000	-3.018148000	-3.508960000
	C	1.177789000	-0.744723000	-1.310758000
	H	1.471474000	-1.269660000	-2.217292000
	P	2.408800000	-0.273002000	-0.177365000
	C	3.948003000	-1.115274000	-0.652903000
	C	4.501686000	-2.127180000	0.142340000
	C	4.564773000	-0.757841000	-1.863541000
	C	5.667059000	-2.778022000	-0.272513000
	H	4.022981000	-2.404983000	1.083034000
	C	5.725374000	-1.414097000	-2.273503000
	H	4.139525000	0.037925000	-2.479907000
	C	6.277804000	-2.423927000	-1.477844000
	H	6.097688000	-3.565461000	0.350150000
	H	6.202675000	-1.134941000	-3.215552000
	H	7.188555000	-2.934565000	-1.799390000
	C	1.995318000	-0.780943000	1.521256000

	C	2.580944000	-0.142081000	2.624487000
	C	1.078499000	-1.822586000	1.721391000
	C	2.258604000	-0.556281000	3.918098000
	H	3.271697000	0.689666000	2.479456000
	C	0.741924000	-2.216279000	3.016432000
	H	0.594306000	-2.301850000	0.870808000
	C	1.336893000	-1.589425000	4.114647000
	H	2.715139000	-0.056079000	4.775322000
	H	-0.017971000	-2.987013000	3.150957000
	H	1.069523000	-1.895134000	5.129133000
	C	2.810180000	1.501032000	-0.134848000
	C	1.825606000	2.436893000	-0.486195000
	C	4.080787000	1.931426000	0.283753000
	C	2.114927000	3.800517000	-0.391289000
	H	0.847192000	2.114108000	-0.845166000
	C	4.359041000	3.296135000	0.370613000
	H	4.855478000	1.202042000	0.531008000
	C	3.372633000	4.230914000	0.039101000
	H	1.343343000	4.525881000	-0.654533000
	H	5.347867000	3.630075000	0.693620000
	H	3.589952000	5.299530000	0.109589000
	C	-3.134808000	4.135864000	1.560767000
	C	-2.966335000	3.024139000	0.732367000
	C	-2.185396000	3.117420000	-0.429309000
	C	-1.583945000	4.343590000	-0.751981000
	C	-1.758732000	5.456823000	0.068763000
	C	-2.532791000	5.353624000	1.230464000
	H	-3.735319000	4.051198000	2.469752000
	H	-3.422705000	2.067561000	0.995196000
	H	-0.983026000	4.393659000	-1.662151000
	H	-1.293030000	6.410184000	-0.194167000
	H	-2.667076000	6.223655000	1.878188000
	C	-1.943173000	1.942423000	-1.334332000
	O	-1.010043000	1.975619000	-2.130417000
	C	-2.861137000	0.791198000	-1.214200000
	H	-3.917633000	1.078753000	-1.155634000
INT-6A-1	Ru	-2.016236000	-0.972913000	-0.031102000
	C	-2.904485000	-2.991771000	1.228653000
	O	-3.599215000	-2.302187000	0.419247000
	O	-1.687653000	-2.687801000	1.400140000
	C	-3.538032000	-4.123610000	1.995104000
	H	-3.863843000	-3.750949000	2.980134000
	H	-4.417536000	-4.505881000	1.460400000
	H	-2.806271000	-4.926490000	2.160473000

	C	0.235100000	-0.765888000	-1.208292000
	O	-0.237368000	0.093179000	-0.353465000
	C	-0.821680000	-1.620047000	-1.817052000
	C	-0.490926000	-2.977561000	-2.152305000
	C	-2.152339000	-1.122350000	-2.169821000
	C	-1.389446000	-3.814774000	-2.758339000
	H	0.500488000	-3.343970000	-1.880603000
	C	-3.049298000	-2.051827000	-2.804196000
	C	-2.690644000	-3.341864000	-3.088877000
	H	-1.113089000	-4.848549000	-2.978565000
	H	-4.047855000	-1.689316000	-3.056894000
	H	-3.405977000	-4.014142000	-3.568914000
	C	1.599615000	-0.968935000	-1.327074000
	H	2.041672000	-1.630494000	-2.068630000
	P	2.642840000	-0.175358000	-0.180382000
	C	4.270650000	-0.977896000	-0.252234000
	C	4.726413000	-1.786138000	0.797789000
	C	5.057034000	-0.813761000	-1.404763000
	C	5.964055000	-2.426948000	0.693999000
	H	4.115327000	-1.913454000	1.693184000
	C	6.289973000	-1.458935000	-1.502833000
	H	4.707453000	-0.175979000	-2.220205000
	C	6.744356000	-2.265145000	-0.453410000
	H	6.318199000	-3.055364000	1.514240000
	H	6.899867000	-1.330404000	-2.399849000
	H	7.711395000	-2.767499000	-0.531162000
	C	2.009999000	-0.313824000	1.521996000
	C	2.346227000	0.642186000	2.492396000
	C	1.141611000	-1.366522000	1.846207000
	C	1.821599000	0.535436000	3.781101000
	H	2.991072000	1.484534000	2.238867000
	C	0.601461000	-1.454269000	3.129466000
	H	0.836673000	-2.091015000	1.091631000
	C	0.945510000	-0.507520000	4.098265000
	H	2.080465000	1.284577000	4.532828000
	H	-0.120062000	-2.246148000	3.333656000
	H	0.518062000	-0.573404000	5.102056000
	C	2.933199000	1.590392000	-0.509382000
	C	1.954180000	2.331747000	-1.189513000
	C	4.109906000	2.212908000	-0.059060000
	C	2.157648000	3.698377000	-1.398003000
	H	1.042257000	1.861907000	-1.565680000
	C	4.301166000	3.578169000	-0.275425000
	H	4.881239000	1.630229000	0.449706000

	C	3.321868000	4.321977000	-0.942027000
	H	1.394825000	4.274286000	-1.925231000
	H	5.217739000	4.060761000	0.071753000
	H	3.472251000	5.390784000	-1.112512000
	C	-3.847768000	3.723062000	0.474177000
	C	-3.453895000	2.579038000	-0.224019000
	C	-2.409653000	2.644170000	-1.161334000
	C	-1.780996000	3.877283000	-1.390714000
	C	-2.170404000	5.019101000	-0.692197000
	C	-3.205112000	4.944240000	0.246688000
	H	-4.660592000	3.659891000	1.202381000
	H	-3.940511000	1.626111000	-0.017348000
	H	-0.980819000	3.905762000	-2.131947000
	H	-1.669405000	5.972659000	-0.878256000
	H	-3.513050000	5.837167000	0.796778000
	C	-1.903514000	1.438706000	-1.917179000
	O	-0.895579000	1.548405000	-2.613113000
	C	-2.631296000	0.178677000	-1.721377000
	H	-3.720616000	0.289483000	-1.759170000
	C	-1.912968000	1.049150000	2.204518000
	O	-2.555915000	0.117263000	1.708765000
	O	-0.774632000	1.498815000	1.743556000
	H	-0.470208000	0.998289000	0.934461000
	C	-2.407879000	1.779381000	3.416080000
	H	-1.636254000	1.755928000	4.200304000
	H	-2.577146000	2.833073000	3.145499000
	H	-3.338496000	1.328270000	3.777733000
TS-7A	Ru	-1.710878000	-1.383962000	0.075331000
	C	-2.088162000	-3.834842000	0.371882000
	O	-3.036007000	-3.063310000	0.043871000
	O	-0.930319000	-3.321641000	0.505592000
	C	-2.316867000	-5.296756000	0.629341000
	H	-2.409808000	-5.457750000	1.715672000
	H	-3.242715000	-5.630127000	0.143050000
	H	-1.458641000	-5.883012000	0.272239000
	C	0.294883000	-0.353796000	-1.104077000
	O	-0.139738000	-0.021266000	0.065738000
	C	-0.720278000	-1.093410000	-1.912392000
	C	-0.289208000	-2.157822000	-2.769618000
	C	-2.137479000	-0.748413000	-1.897662000
	C	-1.168372000	-2.855528000	-3.557310000
	H	0.770687000	-2.417914000	-2.762896000
	C	-3.008634000	-1.495280000	-2.761996000
	C	-2.548979000	-2.515031000	-3.553427000

	H	-0.811011000	-3.673766000	-4.186748000
	H	-4.068621000	-1.232336000	-2.764713000
	H	-3.246558000	-3.070142000	-4.185186000
	C	1.633421000	-0.217133000	-1.444053000
	H	2.021666000	-0.416064000	-2.440602000
	P	2.733091000	0.263559000	-0.185727000
	C	4.425094000	-0.094609000	-0.745356000
	C	5.193625000	-1.095662000	-0.136553000
	C	4.947098000	0.628230000	-1.830984000
	C	6.477975000	-1.372597000	-0.613457000
	H	4.787475000	-1.655951000	0.707619000
	C	6.228071000	0.344030000	-2.304532000
	H	4.352942000	1.416448000	-2.299739000
	C	6.994451000	-0.655934000	-1.695576000
	H	7.075951000	-2.152279000	-0.136218000
	H	6.631760000	0.906491000	-3.149474000
	H	7.998712000	-0.874707000	-2.066072000
	C	2.440122000	-0.649415000	1.360098000
	C	2.803487000	-0.102464000	2.599236000
	C	1.814321000	-1.902578000	1.302765000
	C	2.547777000	-0.814723000	3.772600000
	H	3.262301000	0.885759000	2.654636000
	C	1.538154000	-2.596925000	2.479431000
	H	1.495333000	-2.319166000	0.347863000
	C	1.910075000	-2.057835000	3.713835000
	H	2.828890000	-0.385812000	4.737283000
	H	0.994948000	-3.540540000	2.415584000
	H	1.686702000	-2.599538000	4.636188000
	C	2.710788000	2.040433000	0.211472000
	C	1.530988000	2.765040000	-0.014496000
	C	3.838739000	2.675452000	0.756326000
	C	1.480583000	4.118598000	0.327042000
	H	0.663459000	2.281972000	-0.464854000
	C	3.778972000	4.029583000	1.090912000
	H	4.765625000	2.117452000	0.908210000
	C	2.597898000	4.749535000	0.881405000
	H	0.557914000	4.677441000	0.155506000
	H	4.657165000	4.524869000	1.511795000
	H	2.552828000	5.809073000	1.145074000
	C	-4.898539000	3.937147000	0.283886000
	C	-4.201043000	2.788214000	-0.095194000
	C	-2.968418000	2.893814000	-0.758132000
	C	-2.454169000	4.173227000	-1.035215000
	C	-3.149115000	5.319026000	-0.656371000

	C	-4.377104000	5.202819000	0.005433000
	H	-5.853925000	3.841195000	0.805086000
	H	-4.611148000	1.813149000	0.159249000
	H	-1.500560000	4.235535000	-1.562008000
	H	-2.738618000	6.306923000	-0.879944000
	H	-4.926796000	6.099320000	0.303249000
	C	-2.156520000	1.714923000	-1.221609000
	O	-1.086511000	1.915468000	-1.777416000
	C	-2.700086000	0.323786000	-1.032220000
	H	-3.794852000	0.319929000	-1.097641000
	C	-2.681244000	-0.224292000	2.480947000
	O	-2.079063000	-1.273690000	2.119189000
	O	-3.075896000	0.663749000	1.669469000
	H	-2.731204000	0.264194000	0.439504000
	C	-2.932409000	-0.009013000	3.951403000
	H	-2.354405000	0.867141000	4.284424000
	H	-3.995806000	0.220325000	4.114342000
	H	-2.636515000	-0.890474000	4.532652000
INT-7A	Ru	2.916613000	0.265268000	0.272109000
	C	5.187115000	0.493811000	-0.703538000
	O	4.892048000	-0.476937000	0.055512000
	O	4.264426000	1.333551000	-0.951133000
	C	6.544087000	0.623692000	-1.329574000
	H	6.800935000	1.682642000	-1.468128000
	H	7.299569000	0.116349000	-0.715320000
	H	6.517477000	0.143261000	-2.321164000
	C	0.606204000	0.735030000	-0.914511000
	O	0.993531000	1.194282000	0.218534000
	C	1.709252000	-0.028662000	-1.580994000
	C	2.288377000	0.382350000	-2.819714000
	C	2.004438000	-1.331176000	-1.031585000
	C	3.226786000	-0.402641000	-3.441675000
	H	1.996615000	1.347168000	-3.238682000
	C	2.974492000	-2.116361000	-1.742025000
	C	3.589476000	-1.659015000	-2.881070000
	H	3.693908000	-0.067083000	-4.370561000
	H	3.213277000	-3.109751000	-1.353467000
	H	4.338389000	-2.278339000	-3.380568000
	C	-0.682779000	0.802388000	-1.416106000
	H	-0.907271000	0.316747000	-2.363091000
	P	-2.028073000	1.217049000	-0.399703000
	C	-3.522946000	0.938520000	-1.389661000
	C	-4.583364000	1.853949000	-1.395430000
	C	-3.615062000	-0.253057000	-2.131114000

	C	-5.733843000	1.579436000	-2.139767000
	H	-4.509729000	2.782179000	-0.825882000
	C	-4.768594000	-0.519242000	-2.869238000
	H	-2.788854000	-0.968256000	-2.132127000
	C	-5.828123000	0.394393000	-2.873819000
	H	-6.557326000	2.297021000	-2.147636000
	H	-4.838069000	-1.444300000	-3.445894000
	H	-6.728659000	0.182960000	-3.455185000
	C	-1.997102000	2.935551000	0.182317000
	C	-2.755125000	3.330560000	1.295447000
	C	-1.202501000	3.867124000	-0.498364000
	C	-2.729029000	4.661993000	1.712754000
	H	-3.351864000	2.595596000	1.840132000
	C	-1.175063000	5.195700000	-0.070598000
	H	-0.598721000	3.537570000	-1.346484000
	C	-1.939342000	5.593516000	1.030175000
	H	-3.317201000	4.971955000	2.579511000
	H	-0.546320000	5.920281000	-0.592576000
	H	-1.911853000	6.632974000	1.365580000
	C	-2.217236000	0.196106000	1.103940000
	C	-1.178554000	0.172166000	2.053045000
	C	-3.381066000	-0.559267000	1.322568000
	C	-1.312010000	-0.604354000	3.204212000
	H	-0.263461000	0.734332000	1.871563000
	C	-3.509990000	-1.317661000	2.487206000
	H	-4.186261000	-0.559557000	0.587412000
	C	-2.479307000	-1.338377000	3.429550000
	H	-0.486269000	-0.639388000	3.917533000
	H	-4.414308000	-1.907589000	2.648260000
	H	-2.579995000	-1.943380000	4.333644000
	C	-2.053637000	-4.342937000	1.940593000
	C	-1.083972000	-3.613874000	1.249694000
	C	-1.268089000	-3.299512000	-0.105548000
	C	-2.443572000	-3.720721000	-0.750301000
	C	-3.416497000	-4.436256000	-0.057090000
	C	-3.220162000	-4.753395000	1.292406000
	H	-1.900682000	-4.581794000	2.995377000
	H	-0.190535000	-3.280496000	1.777453000
	H	-2.562197000	-3.480681000	-1.808175000
	H	-4.327123000	-4.758462000	-0.568102000
	H	-3.978206000	-5.321510000	1.837422000
	C	-0.253298000	-2.531058000	-0.908963000
	O	-0.472398000	-2.269827000	-2.077830000
	C	0.993467000	-2.063091000	-0.176034000

	H	1.476587000	-2.926223000	0.307954000
	C	2.766140000	0.245949000	2.781795000
	O	3.484175000	1.079869000	2.154814000
	O	2.137142000	-0.629364000	2.109876000
	H	0.644865000	-1.445104000	0.662746000
	C	2.619800000	0.323185000	4.276853000
	H	2.437709000	-0.675985000	4.695625000
	H	3.513795000	0.774598000	4.726919000
	H	1.754298000	0.963554000	4.515439000
C	C	0.352301000	1.806523000	0.388861000
	O	0.595599000	1.123796000	1.411703000
	C	1.271485000	2.938755000	0.013503000
	C	0.747795000	4.177114000	-0.377818000
	C	2.671543000	2.757599000	0.056662000
	C	1.587265000	5.241842000	-0.714053000
	H	-0.336697000	4.306319000	-0.404390000
	C	3.499798000	3.827265000	-0.299095000
	C	2.970652000	5.065099000	-0.675686000
	H	1.160936000	6.204891000	-1.005848000
	H	4.584257000	3.686216000	-0.282107000
	H	3.640567000	5.886849000	-0.940656000
	C	-0.747861000	1.530499000	-0.451016000
	H	-0.945285000	2.046445000	-1.387749000
	P	-1.625519000	0.091611000	-0.075871000
	C	-2.867480000	-0.127080000	-1.392027000
	C	-4.185741000	-0.507757000	-1.106027000
	C	-2.477484000	0.071337000	-2.728250000
	C	-5.104323000	-0.686559000	-2.143762000
	H	-4.499424000	-0.656608000	-0.071824000
	C	-3.398779000	-0.110446000	-3.760601000
	H	-1.449945000	0.362018000	-2.957201000
	C	-4.713262000	-0.489706000	-3.470274000
	H	-6.131348000	-0.978172000	-1.912090000
	H	-3.088435000	0.045541000	-4.796348000
	H	-5.433494000	-0.629940000	-4.279830000
	C	-2.522614000	0.169298000	1.510542000
	C	-2.985920000	-0.991232000	2.147898000
	C	-2.767795000	1.425501000	2.078081000
	C	-3.701825000	-0.890285000	3.342657000
	H	-2.778667000	-1.972539000	1.715994000
	C	-3.478100000	1.521214000	3.275837000
	H	-2.383578000	2.316040000	1.575705000
	C	-3.947607000	0.365337000	3.907156000
	H	-4.061931000	-1.794749000	3.838632000

	H	-3.660162000	2.501517000	3.722418000
	H	-4.501085000	0.442198000	4.846201000
	C	-0.687392000	-1.480958000	0.002126000
	C	0.121817000	-1.754851000	1.119395000
	C	-0.761723000	-2.412693000	-1.044650000
	C	0.838487000	-2.949679000	1.181424000
	H	0.197338000	-1.013918000	1.913482000
	C	-0.039290000	-3.605848000	-0.973748000
	H	-1.390368000	-2.216420000	-1.913722000
	C	0.759918000	-3.876404000	0.139120000
	H	1.477763000	-3.150515000	2.043099000
	H	-0.104567000	-4.326147000	-1.792684000
	H	1.330440000	-4.805766000	0.191380000
	C	4.508981000	-2.690225000	1.284407000
	C	4.029885000	-1.400303000	1.046508000
	C	3.459835000	-1.073572000	-0.193550000
	C	3.377706000	-2.059462000	-1.188944000
	C	3.869285000	-3.341766000	-0.957587000
	C	4.435350000	-3.661056000	0.281476000
	H	4.944375000	-2.937837000	2.255501000
	H	4.089914000	-0.652141000	1.837527000
	H	2.914126000	-1.788542000	-2.138543000
	H	3.803961000	-4.100837000	-1.740852000
	H	4.817919000	-4.668019000	0.466521000
	C	2.929945000	0.294643000	-0.517389000
	O	2.283047000	0.475572000	-1.529202000
	C	3.269807000	1.431316000	0.444455000
	H	4.368879000	1.504094000	0.503623000
	H	2.900175000	1.140146000	1.438027000
D	C	-3.868317000	-1.805611000	-0.233773000
	C	-2.499385000	-1.971129000	-0.248591000
	C	-1.624966000	-0.857885000	-0.106256000
	C	-2.205001000	0.442185000	0.046297000
	C	-3.615836000	0.587984000	0.060176000
	C	-4.432812000	-0.515748000	-0.077066000
	H	0.209523000	-2.000065000	-0.261887000
	H	-4.524032000	-2.672921000	-0.343044000
	H	-2.063441000	-2.966183000	-0.369012000
	C	-0.212259000	-1.003871000	-0.116689000
	C	-1.324508000	1.560206000	0.183198000
	H	-4.036613000	1.586716000	0.180497000
	H	-5.518832000	-0.397391000	-0.066446000
	C	0.044595000	1.385457000	0.165192000
	C	0.623989000	0.092667000	0.015952000

	H	0.696003000	2.253049000	0.300263000
	O	-1.908160000	2.776181000	0.339495000
	H	-1.222571000	3.450944000	0.428030000
	C	2.100034000	-0.060279000	0.001387000
	C	2.715643000	-1.176148000	0.597297000
	C	2.923572000	0.902324000	-0.610326000
	C	4.102652000	-1.326426000	0.578148000
	H	2.097450000	-1.921101000	1.102384000
	C	4.311058000	0.752482000	-0.630553000
	H	2.468537000	1.765516000	-1.100734000
	C	4.907672000	-0.362709000	-0.036050000
	H	4.558773000	-2.197728000	1.054629000
	H	4.929825000	1.508248000	-1.120699000
	H	5.993861000	-0.479508000	-0.049988000
INT-4AA-1	Ru	0.570829000	-1.046727000	-0.364975000
	C	1.044258000	-3.309679000	-1.487752000
	O	1.982623000	-2.548407000	-1.073313000
	O	-0.149668000	-2.926500000	-1.387998000
	C	1.383184000	-4.668342000	-2.046071000
	H	0.609848000	-4.997645000	-2.752846000
	H	2.369520000	-4.654710000	-2.530140000
	H	1.418317000	-5.390866000	-1.214032000
	C	-1.647485000	-0.650585000	1.437812000
	O	-1.160616000	-0.141638000	0.357594000
	C	-0.775497000	-1.601133000	2.141704000
	C	-1.064417000	-2.169075000	3.393278000
	C	0.363048000	-1.999312000	1.379875000
	C	-0.239828000	-3.160600000	3.918709000
	H	-1.938944000	-1.832760000	3.957137000
	C	1.137117000	-3.049011000	1.914511000
	C	0.851672000	-3.610169000	3.159515000
	H	-0.451216000	-3.595611000	4.898542000
	H	1.974005000	-3.439019000	1.336945000
	H	1.486726000	-4.412575000	3.548705000
	C	-2.982697000	-0.401830000	1.780323000
	H	-3.448311000	-0.839772000	2.660606000
	P	-4.050850000	0.303628000	0.612716000
	C	-5.743406000	-0.008034000	1.203958000
	C	-6.625010000	-0.841008000	0.502728000
	C	-6.149231000	0.574557000	2.416640000
	C	-7.903846000	-1.087597000	1.010594000
	H	-6.311628000	-1.297162000	-0.437780000
	C	-7.426103000	0.323455000	2.919326000
	H	-5.464986000	1.227915000	2.963021000

	C	-8.305280000	-0.507131000	2.215960000
	H	-8.587481000	-1.738611000	0.460843000
	H	-7.737296000	0.778471000	3.862511000
	H	-9.305610000	-0.701692000	2.610037000
	C	-3.908639000	-0.416452000	-1.055782000
	C	-4.541913000	0.207656000	-2.142540000
	C	-3.177860000	-1.595048000	-1.254761000
	C	-4.461616000	-0.361662000	-3.413968000
	H	-5.089718000	1.141168000	-1.998826000
	C	-3.066442000	-2.136830000	-2.535824000
	H	-2.668296000	-2.079102000	-0.423585000
	C	-3.716917000	-1.529289000	-3.611560000
	H	-4.962548000	0.121049000	-4.256659000
	H	-2.437143000	-3.016135000	-2.677242000
	H	-3.628368000	-1.954909000	-4.614222000
	C	-3.932796000	2.108054000	0.378099000
	C	-2.655373000	2.686251000	0.366883000
	C	-5.068572000	2.903832000	0.157362000
	C	-2.515243000	4.052326000	0.117610000
	H	-1.767859000	2.074672000	0.525060000
	C	-4.920375000	4.272795000	-0.079522000
	H	-6.065926000	2.459338000	0.174162000
	C	-3.644541000	4.846100000	-0.104454000
	H	-1.508216000	4.472271000	0.086621000
	H	-5.804565000	4.892511000	-0.247778000
	H	-3.533253000	5.916039000	-0.298262000
	C	-1.122213000	0.786269000	-3.602580000
	C	-0.662679000	1.353722000	-2.433333000
	C	0.549473000	0.910134000	-1.830071000
	C	1.308467000	-0.090699000	-2.497706000
	C	0.812514000	-0.658156000	-3.694632000
	C	-0.389840000	-0.242906000	-4.235559000
	H	-2.067567000	1.122297000	-4.032032000
	H	-1.224565000	2.137960000	-1.928232000
	H	2.326973000	-0.323591000	-2.214717000
	H	1.409570000	-1.425245000	-4.193690000
	H	-0.769574000	-0.697817000	-5.153604000
	C	0.907324000	1.502727000	-0.505849000
	O	0.372050000	2.536036000	-0.098631000
	C	1.662092000	0.582258000	0.423319000
	H	1.367092000	0.822588000	1.459582000
	P	3.437688000	0.713249000	0.478370000
	C	3.944377000	2.329362000	1.157700000
	C	5.310081000	2.660954000	1.209217000

	C	2.981873000	3.250927000	1.593763000
	C	5.710595000	3.894389000	1.719127000
	H	6.058771000	1.952071000	0.847525000
	C	3.393608000	4.489816000	2.097815000
	H	1.917470000	3.027162000	1.494701000
	C	4.750366000	4.809275000	2.168703000
	H	6.772655000	4.146754000	1.762151000
	H	2.642012000	5.209701000	2.429525000
	H	5.064631000	5.777670000	2.565573000
	C	4.187527000	-0.562879000	1.523179000
	C	4.082508000	-1.909631000	1.137406000
	C	4.818650000	-0.228474000	2.733440000
	C	4.648768000	-2.901554000	1.939957000
	H	3.530803000	-2.190070000	0.237854000
	C	5.365054000	-1.231305000	3.535210000
	H	4.879873000	0.811153000	3.055358000
	C	5.290715000	-2.568133000	3.134407000
	H	4.564279000	-3.947361000	1.636146000
	H	5.848565000	-0.965292000	4.477910000
	H	5.722107000	-3.351709000	3.761891000
	C	4.210951000	0.697712000	-1.167442000
	C	4.913583000	-0.400435000	-1.679503000
	C	4.008365000	1.834197000	-1.970709000
	C	5.390978000	-0.368916000	-2.992177000
	H	5.079127000	-1.284965000	-1.066697000
	C	4.484150000	1.856722000	-3.279890000
	H	3.465319000	2.695535000	-1.576362000
	C	5.171465000	0.752755000	-3.794583000
	H	5.932435000	-1.230626000	-3.388631000
	H	4.311100000	2.736490000	-3.903251000
	H	5.538057000	0.769111000	-4.823586000
TS-5AA	Ru	0.373861000	0.842280000	0.444255000
	C	1.091570000	2.198241000	2.551261000
	O	1.882726000	1.668511000	1.689470000
	O	-0.145749000	2.056763000	2.432348000
	C	1.701834000	3.002845000	3.672239000
	H	0.964554000	3.183150000	4.464819000
	H	2.583764000	2.484192000	4.075867000
	H	2.039037000	3.972891000	3.272199000
	C	-2.019855000	1.448769000	-0.986421000
	O	-1.474964000	0.458407000	-0.342645000
	C	-1.115996000	2.556702000	-1.311464000
	C	-1.449651000	3.651196000	-2.123897000
	C	0.170412000	2.442425000	-0.703147000

	C	-0.509536000	4.650689000	-2.364150000
	H	-2.442859000	3.714515000	-2.576970000
	C	1.095141000	3.470544000	-0.959604000
	C	0.761816000	4.552095000	-1.777902000
	H	-0.758362000	5.500413000	-3.004271000
	H	2.087898000	3.422692000	-0.514291000
	H	1.504701000	5.333598000	-1.965396000
	C	-3.389761000	1.400954000	-1.230663000
	H	-3.937754000	2.197057000	-1.730016000
	P	-4.302266000	0.097538000	-0.525221000
	C	-6.059272000	0.528531000	-0.711274000
	C	-6.877395000	0.770758000	0.399833000
	C	-6.585915000	0.652846000	-2.008137000
	C	-8.214845000	1.132745000	0.214124000
	H	-6.469412000	0.677451000	1.407629000
	C	-7.920750000	1.015854000	-2.186915000
	H	-5.950458000	0.459360000	-2.875531000
	C	-8.736739000	1.254881000	-1.075608000
	H	-8.849519000	1.321464000	1.082929000
	H	-8.326937000	1.110977000	-3.196512000
	H	-9.782552000	1.537459000	-1.217438000
	C	-3.976058000	-0.152410000	1.249088000
	C	-4.430531000	-1.316769000	1.887237000
	C	-3.255954000	0.807623000	1.972301000
	C	-4.181677000	-1.504845000	3.247250000
	H	-4.965755000	-2.082317000	1.321885000
	C	-2.978060000	0.596759000	3.323182000
	H	-2.883522000	1.704774000	1.479532000
	C	-3.448391000	-0.552889000	3.962460000
	H	-4.540459000	-2.409546000	3.743807000
	H	-2.363161000	1.326154000	3.852278000
	H	-3.228435000	-0.717670000	5.020070000
	C	-4.101416000	-1.532406000	-1.319982000
	C	-2.827289000	-1.918788000	-1.765323000
	C	-5.188562000	-2.410806000	-1.460540000
	C	-2.643039000	-3.177770000	-2.339130000
	H	-1.976276000	-1.251306000	-1.640829000
	C	-4.996811000	-3.667668000	-2.039738000
	H	-6.185222000	-2.114106000	-1.127377000
	C	-3.725102000	-4.052343000	-2.476635000
	H	-1.636822000	-3.459842000	-2.655252000
	H	-5.844995000	-4.347593000	-2.150240000
	H	-3.579082000	-5.037916000	-2.925765000
	C	-1.155292000	-2.957099000	2.197594000

	C	-0.661562000	-2.702209000	0.926592000
	C	0.451040000	-1.851502000	0.744192000
	C	1.089546000	-1.316171000	1.885509000
	C	0.579827000	-1.580553000	3.166158000
	C	-0.543378000	-2.385202000	3.325143000
	H	-2.035885000	-3.591354000	2.320082000
	H	-1.135396000	-3.126222000	0.042568000
	H	2.008373000	-0.745866000	1.801141000
	H	1.084808000	-1.154289000	4.035797000
	H	-0.946200000	-2.577969000	4.321961000
	C	0.840843000	-1.492357000	-0.664444000
	O	0.472110000	-2.175901000	-1.622760000
	C	1.397494000	-0.128695000	-0.834227000
	H	1.602103000	0.162680000	-1.874275000
	P	3.754278000	-0.347553000	-0.521232000
	C	4.413951000	-1.567050000	-1.718825000
	C	5.761134000	-1.970650000	-1.661714000
	C	3.571931000	-2.108646000	-2.702139000
	C	6.261382000	-2.888154000	-2.584188000
	H	6.416042000	-1.562261000	-0.888919000
	C	4.080318000	-3.031497000	-3.623477000
	H	2.512685000	-1.847947000	-2.736443000
	C	5.420138000	-3.418089000	-3.570090000
	H	7.309378000	-3.193614000	-2.534051000
	H	3.416148000	-3.453892000	-4.380995000
	H	5.811643000	-4.139516000	-4.291608000
	C	4.628176000	1.212685000	-0.887010000
	C	4.353372000	2.342957000	-0.093899000
	C	5.491186000	1.343093000	-1.989278000
	C	4.970908000	3.562924000	-0.375597000
	H	3.629730000	2.270426000	0.721055000
	C	6.092580000	2.571454000	-2.271092000
	H	5.695888000	0.486021000	-2.631258000
	C	5.842214000	3.682625000	-1.461511000
	H	4.750879000	4.432072000	0.249313000
	H	6.761432000	2.658340000	-3.130881000
	H	6.315313000	4.641988000	-1.684436000
	C	4.377211000	-0.968947000	1.082392000
	C	4.798214000	-0.119140000	2.116950000
	C	4.271261000	-2.350397000	1.334099000
	C	5.108999000	-0.641719000	3.374225000
	H	4.877399000	0.953551000	1.947405000
	C	4.582030000	-2.865817000	2.591243000
	H	3.930594000	-3.023694000	0.544896000

	C	4.999315000	-2.013244000	3.618351000
	H	5.439245000	0.031841000	4.168875000
	H	4.490756000	-3.939490000	2.771432000
	H	5.237593000	-2.417181000	4.605076000

Table S3. Imaginary frequency of all the transition states in this work, calculated at the B3LYP+D3BJ/def2SVP level

**Names**	**I. F. (*i* cm^{-1})**	**Names**	**I. F. (*i* cm^{-1})**
**TS-1A**	-13.0	**TS-1B**	-36.2
**TS-2A**	-54.8	**TS-2B**	-65.1
**TS-3A**	-579.4	**TS-3B**	-491.3
**TS-4A**	-119.4	**TS-4B**	-121.7
**TS-5A**	-211.9	**TS-5AA**	-187.3
**TS-6A**	-298.1	**TS-7A**	-1131.7
**TS-1ASol**	-11.8	**TS-2ASol**	-56.8
**TS-3ASol**	-802.1	**TS-4ASol**	-111.0

Table S4. Electronic energis (***E***) and Gibbs free energies (***G***) calculated at the B3LYP+D3BJ/def2SVP level and single-point energies (***E'***) calculated at the B3LYP+D3BJ-PCM/def2TZVPP level

**Names**	***E* (a.u.)**	***G* (a.u.)**	***E'* (a.u.)**
**[Ru(*p*-cymene)Cl_2]$_2$**	-2808.962685	-2808.614461	-2810.518714
**NaOAc**	-390.63063	-390.621958	-391.003246
**Na_2Cl_2**	-1244.858477	-1244.897317	-1245.354148
**CAT**	-940.931113	-940.6848593	-941.9338353
**A**	-1419.239889	-1418.922035	-1420.550407
**TS-1A**	-2360.195492	-2359.594564	-2362.494033
**INT-1A**	-2360.215357	-2359.611345	-2362.507152
**TS-2A**	-2360.173696	-2359.572184	-2362.475346
**INT-2A**	-2360.177496	-2359.575573	-2362.480037
**TS-3A**	-2360.170287	-2359.571493	-2362.464482
**INT-3A**	-2131.255182	-2130.708603	-2133.28261
**HOAc**	-228.924281	-228.8976217	-229.2055412
**TS-4A**	-2131.230227	-2130.684216	-2133.253835
**INT-4A**	-2131.236467	-2130.691304	-2133.259646
**p-cymene**	-389.272904	-389.1121522	-389.7030918
**B**	-936.374969	-936.2330499	-937.1694523
**INT-4A-1**	-2678.37060	-2677.839861	-2680.748546
**TS-5A**	-2678.367408	-2677.839537	-2680.742855

**DMSO**	-552.969052	-552.9304478	-553.3336217
**INT-5A**	-2125.398985	-2124.942837	-2127.424501
**TS-6A**	-2125.359001	-2124.904364	-2127.384931
**INT-6A**	-2125.378794	-2124.92321	-2127.409294
**INT-6A-1**	-2354.370699	-2353.85801	-2356.658004
**TS-7A**	-2354.342054	-2353.835619	-2356.631073
**INT-7A**	-2354.366782	-2353.854485	-2356.661444
**C**	-1802.702955	-1802.278084	-1804.435227
**O=PPh$_3$**	-1110.994228	-1110.777728	-1111.98717
**D**	-691.743	-691.5651079	-692.504509
**TS-1B**	-1877.316548	-1876.89421	-1879.102777
**INT-1B**	-1877.329721	-1876.905203	-1879.112987
**TS-2B**	-1877.285326	-1876.862885	-1879.080625
**INT-2B**	-1877.296168	-1876.872953	-1879.085244
**TS-3B**	-1877.288613	-1876.868873	-1879.072497
**INT-3B**	-1648.376361	-1648.00682	-1649.888656
**TS-4B**	-1648.355605	-1647.987732	-1649.866285
**INT-4B**	-1648.362906	-1647.995669	-1649.873664
**INT-4AA-1**	-3161.231031	-3160.52284	-3164.124359
**TS-5AA**	-3161.208994	-3160.504152	-3164.099508
**PPh$_3$**	-1035.800556	-1035.587783	-1036.677191
**CAT-Sol**	-1250.844862	-1250.451458	-1252.209304
**TS-1ASol**	-2670.110866	-2669.364054	-2672.769613
**INT-1ASol**	-2670.133474	-2669.384019	-2672.788986
**TS-2ASol**	-2670.093151	-2669.345916	-2672.754429
**INT-2ASol**	-2670.098509	-2669.352734	-2672.757454
**TS-3ASol**	-2670.092417	-2669.348792	-2672.74441
**HOAc-Sol**	-383.883636	-383.7879456	-384.343351
**INT-3ASol**	-2286.209713	-2285.589021	-2288.417026
**TS-4ASol**	-2286.188576	-2285.570937	-2288.392455
**INT-4ASol**	-2286.193176	-2285.575093	-2288.397854

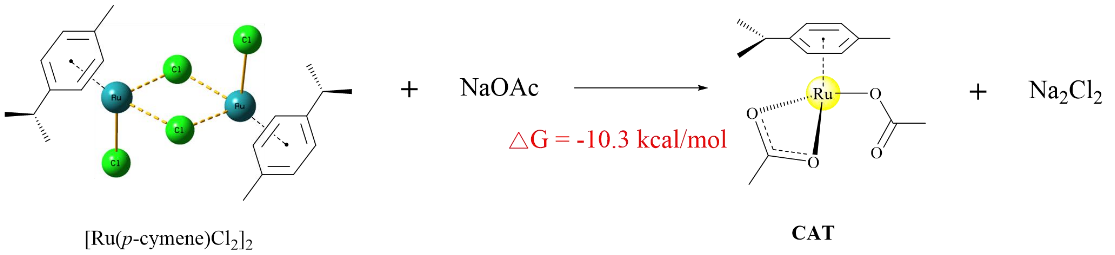

Figure S1. Energetics for the generation of the active catalyst, calculated at the B3LYP+D3BJ-PCM/def2TZVPP//B3LYP+D3BJ/def2SVP level

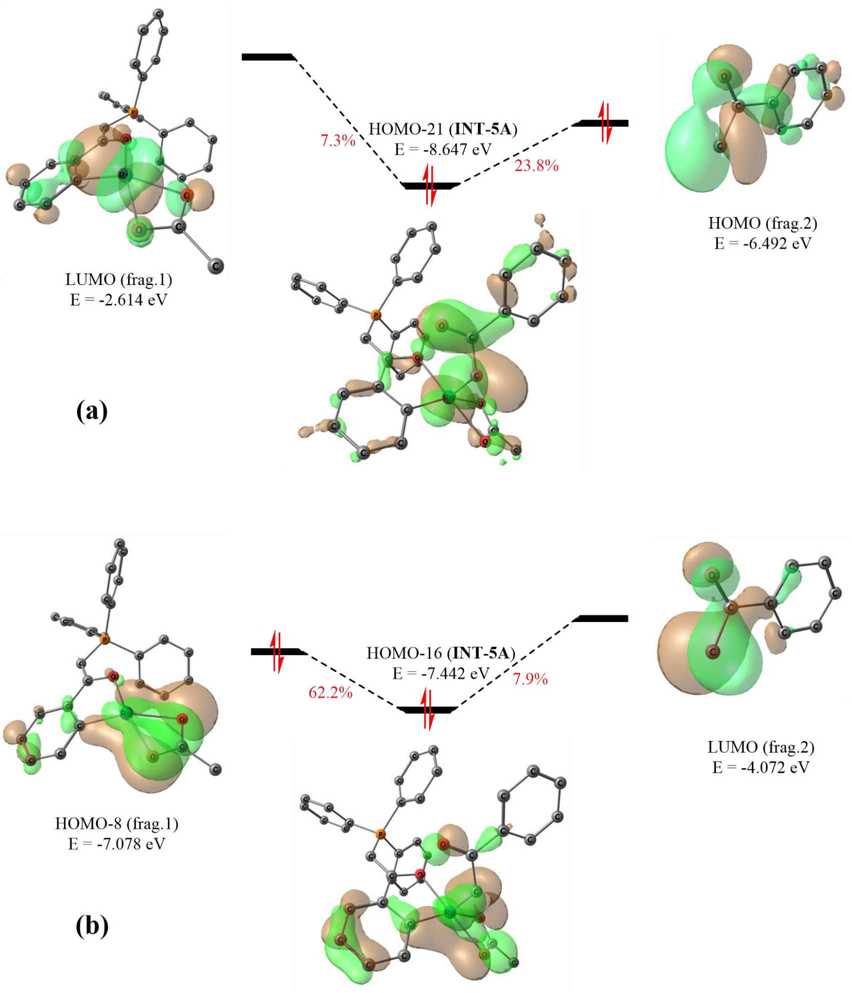

Figure S2. Orbital interation diagrams between two fragments in **INT-5A**, generated at the B3LYP+D3BJ/def2SVP level
